# Theory for Identification and Inference with Synthetic Controls: A Proximal Causal Inference Framework

**DOI:** 10.1080/01621459.2026.2639734

**Published:** 2026-06-04

**Authors:** Xu Shi, Kendrick Qijun Li, Myeonghun Yu, Wang Miao, Arun Kumar Kuchibhotla, Mengtong Hu, Eric Tchetgen Tchetgen

**Affiliations:** a Department of Biostatistics, University of Michigan, Ann Arbor, MI; b Department of Biostatistics, St. Jude Children’s Research Hospital, Memphis, TN; c Department of Probability and Statistics, Peking University, Beijing, China; d Department of Statistics and Data Science, Carnegie Mellon University, Pittsburgh, PA; e Statistics Department, The Wharton School, University of Pennsylvania, Philadelphia, PA

**Keywords:** Panel data, Proximal causal inference, Synthetic control, Time series

## Abstract

Synthetic control (SC) methods are commonly used to estimate the treatment effect on a single treated unit in panel data settings. An SC is a weighted average of control units built to match the treated unit, with weights typically estimated by regressing pretreatment outcomes and measured covariates of the treated unit to those of the control units. However, the classical SC method was primarily proposed for empirical settings where a good pretreatment fit is attainable. In this article, we introduce a proximal causal inference framework to formalize identification and inference for both the SC and ultimately the treatment effect on the treated, based on the observation that control units not contributing to the construction of an SC can be repurposed as proxies of latent confounders, thus, extending the applicability of SC methods to cases where the pretreatment fit is poor even with many pretreatment periods. We show that several existing uncertainty quantification methods of treatment effect for the classical SC methods can be adapted to the proximal inference approach. The proposed framework can accommodate nonlinear models, which allows for binary and count outcomes both of which remain understudied in the SC literature. We illustrate with comprehensive simulation studies and an application to the evaluation of the 1990 German Reunification. Supplementary materials for this article are available online, including a standardized description of the materials available for reproducing the work.

## 1 Introduction

Synthetic control (SC) methods are commonly used to estimate the impact of an intervention when one has observed time series data on a single treated unit and multiple untreated units in both pre- and post-treatment periods (Abadie and Gardeazabal 2003; Abadie, Diamond, and Hainmueller 2010). The treated unit is matched to a weighted average of control units, referred to as an “SC”, such that the SC’s post-treatment outcome predicts the treated unit’s unobserved potential outcome under no treatment. In practice, this is often operationalized by regressing the pretreatment outcome and covariates of the treated unit on those of the control units using ordinary or weighted least squares (OLS/WLS) under the constraints that the coefficients are nonnegative and sum to one, and by taking the estimated regression coefficients as SC weights. The treatment effect on the treated unit is then estimated as the difference in post-treatment outcomes between the treated unit and its SC forecast. The SC method has become increasingly popular in recent years with a surge of new methods including methods for multiple treated units (Abadie and L’hour 2021; Ben-Michael, Feller, and Rothstein 2022), matrix completion and matrix estimation (Athey et al. 2021; Bai and Ng 2021; Amjad, Shah, and Shen 2018), penalization (Doudchenko and Imbens 2016; Ben-Michael, Feller, and Rothstein 2021), and uncertainty quantification (Li 2020; Chernozhukov, Wüthrich, and Zhu 2021; Cattaneo, Feng, and Titiunik 2021; Cattaneo et al. 2025a).

The original SC method of Abadie, Diamond, and Hainmueller (2010) was shown to have good theoretical properties assuming a good pretreatment fit can be obtained in the observed data, that is, there exists a set of weights such that the pretreatment outcomes and covariates of the treated unit can be approximated well by a weighted average of those among the control units. In practice, Abadie, Diamond, and Hainmueller (2010) suggested empirically checking if the characteristics of the SC and the treated unit are sufficiently matched in the pretreatment period and recommended not to use an SC if the fit is poor. In case the pretreatment fit is poor, an alternative framework considered in the literature is to assume a perfect match of the underlying unobserved factor loadings rather than the observed data (Amjad, Shah, and Shen 2018; Powell 2018; Ferman 2021; Ferman and Pinto 2021). In this setting, Ferman and Pinto (2021) showed that the OLS estimates of the SC weights can be inconsistent even when the number of pretreatment periods is large. Intuitively, the pretreatment outcome trajectories of control units are proxies of time-varying latent factors but are measured with error. When such proxies of the latent factors are used as independent variables in OLS/WLS, estimated regression coefficients will generally be inconsistent due to the well-known errors-in-variables regression problem (Carroll et al. 2006). To address this problem, Ferman and Pinto (2021) proposed to use lagged control unit outcomes as instrumental variables to construct unbiased estimating equations. Amjad, Shah, and Shen (2018) proposed to de-noise the data via singular value thresholding. However, both required the idiosyncratic error terms to be independent across units and time.

In this article, focusing on settings where the factor loadings are well matched but the pretreatment fit of observed outcomes may be poor, we leverage a different perspective of control units embraced in recent SC literature (Abadie and Vives-i Bastida 2022): that the outcomes of control units are proxies for the unmeasured factors. Rather than directly regressing the outcome on all such proxies, one can split the set of proxies into two, thus, leveraging one set of proxies to assist the construction of a SC defined in terms of the other set of proxies. Generally, not all control units contribute to the construction of an SC in practice. The outcomes of control units not included in the SC can be repurposed to construct identifying moment equations of SC weights. Besides outcomes of control units, measured covariates contemporaneous with outcomes of the treated and control units can also serve as candidate proxies. As we show in this article, leveraging such proxies of latent factors can yield identification of SC weights under certain conditions, which is motivated by recent work on proximal causal inference (Miao, Geng, and Tchetgen Tchetgen 2018; Tchetgen Tchetgen et al. 2024). Under certain conditions, our methods produce consistent estimators of SC weights and the treatment effect allowing for serial correlation of error terms, as well as both stationary and nonstationary latent factors (Hsiao, Ching, and Wan 2012; Ferman and Pinto 2021). We also establish nonparametric identification which relaxes the linear factor model assumption. To accommodate inference on various treatment effects of interest, we adapt three different approaches to the proximal inference framework: the conformal permutation inference approach of Chernozhukov, Wüthrich, and Zhu (2021), the prediction intervals methods of (Cattaneo, Feng, and Titiunik 2021; Cattaneo et al. 2025a), and the GMM framework (Hansen 1982).

Our article is organized as follows. In Section 2, we review classical SC methods and discuss the potential failure of existing SC methods under a poor pretreatment fit of OLS/WLS estimation, even if a perfect match of latent factor loadings can reasonably be assumed. We then propose a proximal causal inference method for consistent estimation and formal statistical inference in Section3. Specifically, we first focus on estimation of the SC weights in Section 3.1. Then we introduce three possible approaches of inference for treatment effect trajectory or average post-treatment effect on the treated unit in Section c. We then show that the fixed effects model and the corresponding identification conditions are a special case of a nonparametric identification framework in Section 3.3, which accommodates nonlinear models and different types of outcomes such as binary or count outcomes. We illustrate our proposed methods with simulation studies in Section 4 and an application to the evaluation of the 1990 German reunification in Section 5. Throughout, notions of consistency and valid statistical inference about causal effects are studied under an asymptotic regime introduced in Ferman and Pinto (2021) with diverging time points but a fixed number of units. We close with a brief discussion in Section 6. Various extensions of interest are discussed in the supplementary material, including a proximal synthetic control approach without assuming a unique SC in Section E, and a ridge-regularized estimator tailored to cases with few pretreatment periods in Section G.

## 2. Review of Classical Synthetic Control Methods

Suppose N+1 units indexed by i=0,…,N are observed over T time periods indexed by t=1,…,T. Consider a binary treatment that impacts unit i=0 after a certain time point. Let $T_{0}$ and $T_{1}=T-T_{0}$ denote the number of pre- and post-treatment periods, respectively. Units i=1,…,N are untreated control units. The article primarily focuses on the setting where N is fixed and $T_{0}$ is large, with the exception of Section 3.2.3 where we further assume $T_{1}$ is also large and roughly of the same magnitude as $T_{0}$. The permutation inference approach in Section 3.2.1 accommodates a short post-treatment period. Let $\left(Y_{t}(1),Y_{t}(0)\right)$ and $\left(W_{it}(1),W_{it}(0)\right)$ denote the potential outcomes of the treated unit and the *i*th control unit, respectively, corresponding to whether the treated unit is, possibly contrary to fact, assigned or not assigned to treatment at time t. Note that the potential outcomes for control units are defined relative to the treatment status of the treated unit. We are interested in the treatment effect on the treated unit at time t post-treatment, that is, $\theta_{t}=Y_{t}(1)-Y_{t}(0)$ for any $t>T_{0}$. In panel data literature, the effect sequence $\theta_{t}$ is often considered fixed for each t (Bai 2009; Athey et al. 2021). In the SC literature, Chernozhukov, Wüthrich, and Zhu (2021) proposed uncertainty quantification methods in cases where $\theta_{t}$ is either fixed or random, while Cattaneo, Feng, and Titiunik (2021) and Cattaneo et al. (2025a) primarily focused on predictive inference for random $\theta_{t}$. In this article, unless otherwise specified, we will consider $\theta_{t}$ as a random treatment effect sequence and “predictand” of interest (Cattaneo et al. 2025a). A corresponding estimand of interest is the expected treatment effect on the treated unit (ETT) at time t, that is, $\tau_{t}=E\left(\theta_{t}\right)$, $t>T_{0}$, with $\tau_{t}=\theta_{t}$ if $\theta_{t}$ is considered fixed. Other predictands or estimands of potential interest include the average post-treatment effect $T_{0}$), a finite-dimensional parameter $\gamma \in R^{k}$ that indexes a parametric model for ETT $\tau_{t}=\tau (t/T;\gamma )$, or the limit of the expected average post-treatment effect $\bar{\tau }=\lim_{T\to \infty }\sum_{t=T_{0}+1}^{T}\tau_{t}/\left(T-T_{0}\right)=\lim_{T\to \infty }E(\bar{\theta })$, assuming the latter is well defined.

### Remark 1.

Fixed treatment effects $\theta_{t}$ may be implied by a structural equation $Y_{t}(x)=s_{t}(x)+e_{t}(x)$ for *x* = 0, 1 at each t, where $s_{t}(x)$ is a deterministic function and $e_{t}(x)$ is a random error, with the additional restriction that $e_{t}(0)=e_{t}(1)$ at each t. Under this setting, we have $\theta_{t}=s_{t}(1)-s_{t}(0)$.

Let $Y_{t}$ and $W_{it}$ denote the observed outcome of the treated unit and the *i*th control unit, respectively, at time period t. We assume that the observed outcome is a realization of the potential outcome under the assigned treatment value:

### Assumption 1 (Consistency).

For any unit i, $Y_{t}=Y_{t}(0)$ and $W_{it}=W_{it}(0)$ for $t\leq T_{0}$ and $Y_{t}=Y_{t}(1)$ and $W_{it}=W_{it}(1)$ for $t>T_{0}$.

A common SC model in the literature assumes that the potential outcomes follow the data generating mechanism (Bai 2009; Abadie, Diamond, and Hainmueller 2010; Chernozhukov, Wüthrich, and Zhu 2021) below:

### Assumption 2 (Interactive fixed effects model).

For any unit i and time t,

(1) $$Y_{t}(0)=\mu_{0}^{⊤}\lambda_{t}+\varepsilon_{0t},$$

(2) $$W_{it}(0)=W_{it}(1)=\mu_{i}^{⊤}\lambda_{t}+\varepsilon_{it}$$

for t=1,…,T and i=0,…,N, where $\lambda_{t}$ is an r×1 vector of random latent factors, $\mu_{i}\in R^{r}$ is an r×1 vector of unit-specific factor loadings (assumed to be fixed), and $\varepsilon_{it}$ is the error term with $E\left[\varepsilon_{it}|\lambda_{t}\right]=E\left[\varepsilon_{it}\right]=0$ for all i and t.

The equivalence $W_{it}(0)=W_{it}(1)$ in (2) implies no interference, that is, the treatment assigned to the treated unit does not impact the outcomes of the control units. Equation (2) additionally implies that the treatment status of the treated unit at each time period is not exogenous but rather depends on $\mu_{i}^{⊤}\lambda_{t}$,i=0,…,N. The interactive effect $\mu_{i}^{⊤}\lambda_{t}$ can be viewed as the main source of unmeasured confounding because it is both associated with the time-dependent treatment status and predictive of the outcome. Therefore, we also refer to $\lambda_{t}$ as the unmeasured confounder (Ferman and Pinto 2021).

### Remark 2.

Our proposed method can also accommodate additive fixed effects (a special case of $\mu_{i}^{⊤}\lambda_{t}$) with $Y_{t}(0)=\mu_{0}^{⊤}\lambda_{t}+\delta_{t}+\zeta_{0}+\varepsilon_{0t}$ and $W_{it}=\mu_{i}^{⊤}\lambda_{t}+\delta_{t}+\zeta_{i}+\varepsilon_{it}$, i=1,…,N, as considered in Ferman and Pinto (2021), where factor loadings of the unknown common factors $\delta_{t}$ remain constant across units and the unknown intercepts $\zeta_{i}$ are unit-specific.

Under Assumptions 1 and 2, we have

(3) $$Y_{t}=\left\{\begin{array}{ll} Y_{t}(0)=\mu_{0}^{⊤}\lambda_{t}+\varepsilon_{0t} & \text{when}\;t\leq T_{0},\\ Y_{t}(1)=Y_{t}(0)+\theta_{t} & \text{when}\;T_{0}<t<T,\;\text{(4)} \end{array}\right.$$

$W_{it}=W_{it}(1)=W_{it}(0)=\mu_{i}^{⊤}\lambda_{t}+\varepsilon_{it}$, for any i,t.

**Figure 1.**
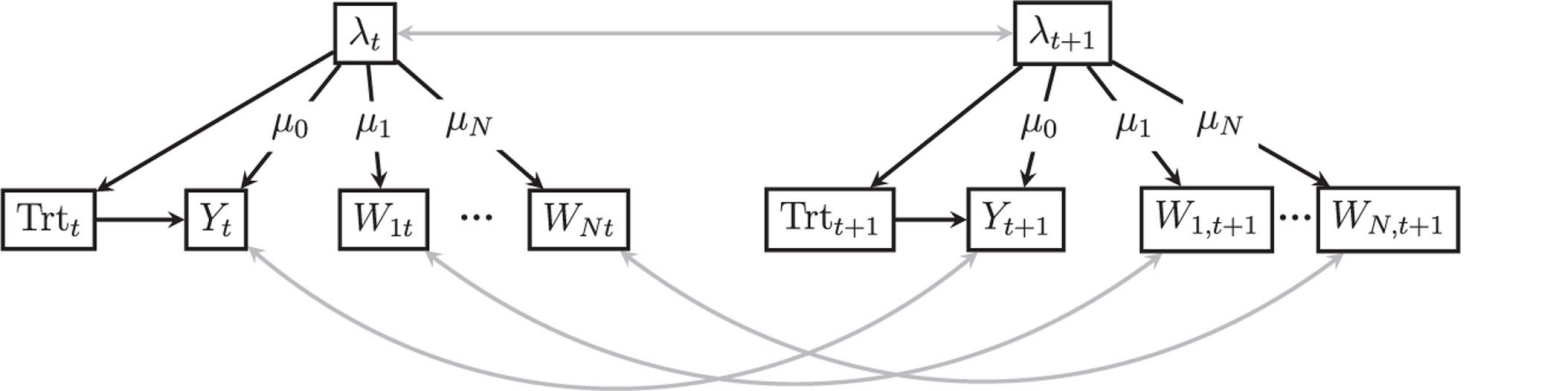
A graphical illustration of the relationships among the treatment status at each time point $\left(\text{Tr}{\text{t}}_{\text{t}}\right)$, the observed outcomes of the treated unit $Y_{t}$ and the control units $W_{it}$, i≠0, and the unobserved common factor $\lambda_{t}$ at each time point t. Measured covariates are suppressed for simplicity.

In the post-treatment period, $Y_{t}(1)=Y_{t}$ is always observed while $Y_{t}(0)$ is unobserved. Figure 1 presents a graphical illustration of the above model assumptions on the observed data. Our goal is to find an SC such that the post-treatment outcome of the SC can predict the unobserved $Y_{t}(0)$ of the treated unit.

A key assumption of the proposed SC methods is that the unmeasured confounding effect on the treated unit can be matched by a weighted average of the unmeasured confounding effect on a set of the control units, referred to as the “donor pool”. Let D index the control units selected into the “donor pool”, and let |D| denote the number of control units in the donor pool. For a sequence of unit-specific values $a_{i}\in R$, i=1,…,N, let $a_{D}={\left(a_{i,i\in D}\right)}^{⊤}$ denote the |D|-vector of $a_{i}$’s for i∈D, that is, units in the donor pool; if $a_{i}\in R^{d}$ is a *d*-dimensional vector, then $a_{D}$ is a d×|D| matrix. Similarly, let $W_{Dt}={\left(W_{it,i\in D}\right)}^{⊤}$ denote the |D|×1 vector of $W_{it}$’s for i∈D. The assumption below is key to the identification of an SC, an extension of which to nonlinear settings is provided in Section 3.3.

### Assumption 3 (Existence of synthetic control).

There exist a set of weights $\alpha_{D}$ with $\alpha_{i}\in R$, i∈D such that $\mu_{0}=\sum_{i\in D}\alpha_{i}\mu_{i}$.

As we formalize below in Theorem 1, this assumption identifies $E\left[Y_{t}(0)\right]$, the mean potential outcome of the treated unit under no treatment in the post-treatment period, proved in Section A of the supplementary material.

### Theorem 1.

Under Assumptions 1–3, we have $E\left[Y_{t}(0)\right]=E\left[\sum_{i\in D}\alpha_{i}W_{it}\right]$ for any t, and the ETT at time t for any $t>T_{0}$ is $\tau_{t}=E\left[Y_{t}-\sum_{i\in D}\alpha_{i}W_{it}\right]$.

### Remark 3.

Abadie, Diamond, and Hainmueller (2010) assume that the weights are nonnegative and sum to one, such that the SC lies in the convex hull of the donors’ characteristics. The sum-to-one condition is implied by Assumption 3 when there exist unknown common factors $\delta_{t}$ with constant factor loadings as discussed in Remark 2. The nonnegative condition is primarily useful for interpretation. Because $\alpha_{D}$ is estimated by regression, when the number of parameters, |D|, is larger than the sample size, the convex hull assumption can bypass the need for regularization. Relaxations of the convex hull restriction are discussed in Doudchenko and Imbens (2016). It is important to note that while $\alpha_{D}$ satisfying Assumption 3 may not be unique, that is, there may not be a unique SC, all SCs satisfying Assumption 3 lead to the same $E\left[Y_{t}(0)\right]$ using our proposed method, for any t as stated in Theorem 1. As such, the ETT at time t is identified as long as one can identify at least one set of SC weights $\alpha_{D}$ satisfying Assumption 3. See Section E of the supplementary material for more details. Our proposed method can further impose the restriction that SCs fall within the convex hull of donor characteristics, which, as argued by Abadie, Diamond, and Hainmueller (2010), can avoid extrapolation. See Sections 3.1–3.2 for more details.

Under Assumptions 2 and 3 we have that in the pretreatment period,

(5) $$Y_{t}=\sum_{i\in D}\alpha_{i}W_{it}+\left(\varepsilon_{0t}-\sum_{i\in D}\alpha_{i}\varepsilon_{it}\right),\text{for}\;\text{any}t\leq T_{0}.$$

Thus, a natural approach to estimate $\alpha_{D}$ is regression using pretreatment data, viewing the observed outcomes $W_{it}=\mu_{i}^{⊤}\lambda_{t}+\varepsilon_{it}$ as proxies of the unobserved latent factors $\lambda_{t}$ with measurement error $\varepsilon_{it}$. The following constrained OLS is widely used to construct and SC

(6) $${\hat{\alpha }}_{D}=\underset{\alpha_{i}\geq 0,\sum_{i\in D}\alpha_{i}=1}{\arg\min}\frac{1}{T_{0}}\sum_{t=1}^{T_{0}}{\left(Y_{t}-\sum_{i\in D}\alpha_{i}W_{it}\right)}^{2}.$$

However, the (constrained) OLS weights are generally inconsistent even when $T_{0}\to \infty$, and thus, treatment effect estimates will generally be biased (Ferman and Pinto 2021). This is because in (5), the residual $\varepsilon_{0t}-\sum_{i\in D}\alpha_{i}\varepsilon_{it}$ is correlated with the regressor $W_{Dt}$, therefore, the coefficients ${\hat{\alpha }}_{D}$ obtained from (constrained) OLS is inconsistent unless $\varepsilon_{it}$ is zero, that is, a noiseless setting. The inconsistency of ${\hat{\alpha }}_{D}$ in the large $T_{0}$ setting has been pointed out by Ferman and Pinto (2021) and Powell (2018). They showed that ${\hat{\alpha }}_{D}$ converges to a set of weights that not only attempt to match the latent factors as in Assumption 3, but also strain to minimize the noise in the synthetic outcome.

It is important to note that this result does not necessarily conflict with that of Abadie, Diamond, and Hainmueller (2010), because rather than assuming a perfect match of $\mu_{i}$, they require a good match of the observed outcomes and covariates with the chosen control units such that the right-hand side of (6) is approximately zero. Under certain regularity conditions, they established that the bias of the estimated treatment effect is bounded by a function that goes to zero as $T_{0}$ goes to infinity, or as the maximal variance of $\varepsilon_{it}$ goes to zero. This provides theoretical guarantees a user might expect in a setting where a good fit can be obtained in the pretreatment period. As suggested by Abadie, Diamond, and Hainmueller (2010), one should empirically verify whether there is a good match to the treated unit that can serve as an SC, and it is recommended not to use an OLS-based SC if the pretreatment characteristics of the treated unit and the SC are not adequately matched. Our approach introduced below complements traditional SC methods by focusing on an exact match of the underlying population parameters rather than a close fit to the observed data, which accommodates imperfect pretreatment fit and extends the applicability of SC.

## 3. A Proximal Causal Inference Approach to SC

In this section, we introduce a proximal causal inference framework for SC. Our proposal mitigates the potential of inconsistency of the estimated SC weights and treatment effects by leveraging proxies that are not included in the construction of an SC for identification of the SC. We also describe several approaches for inference about treatment effects under the proximal framework. Furthermore, we demonstrate that the SCs may be constructed by a general function of the donors’ outcomes and measured covariates, which goes beyond the linear combination of the control units and extends the linear interactive fixed effects model. For ease of exposition, we introduce our framework ignoring measured covariates unless stated explicitly. Technical details of our approach to incorporate measured covariates are relegated to Section C of the supplementary material.

### 3.1. Identification and Estimation of Synthetic Control Weights

Theorem 1 illustrates the pivotal role the SC weights play in the identification of treatment effects. As discussed in Section 2, in the presence of latent variables $\lambda_{t}$, direct regression adjustment of $W_{Dt}$ as valid proxies of $\lambda_{t}$ will generally fail to be consistent due to measurement errors $\varepsilon_{it}$. However, as we establish below, consistent estimation is possible if one has access to supplemental proxies which, although not used directly to form an SC, may be used to identify SC weights. Specifically, suppose that additional proxies of $\lambda_{t}$ are available, which are a priori known to be associated with the treated and donor units in the pretreatment period only through $\lambda_{t}$. Formally,

### Assumption 4 (Existence of proxy).

We have observed $Z_{t}$ such that $Z_{t}\;⊥\left\{Y_{t},W_{Dt}\right\}|\lambda_{t}$ for any $t\leq T_{0}$.

We argue that such proxy variables are often available in SC settings. Assuming independence of the error terms between the donors and the rest of the control units, that is, $\left\{\varepsilon_{0t},\varepsilon_{Dt}\right\}\;⊥\varepsilon_{\bar{D}t}$ where $\bar{D}=\{1,\ldots ,N\}\backslash D$, then as mentioned in Section 1, a reasonable candidate for proxy variables is the outcome of units excluded from the donor pool, that is, $Z_{t}=W_{{\bar{D}}_{t}}$. In fact, it is customary to restrict D to units with similar pretreatment outcome trajectories and covariates, as suggested by Abadie (2021). In addition, a control unit is often excluded from D if it might experience a spillover effect from the treatment on the treated unit or an intervention that conflicts with the control condition under consideration, both of which violate the no-interference assumption in (2), but do not invalidate such units as valid proxy $Z_{t}$. For instance, in Abadie, Diamond, and Hainmueller (2010), 38 out of 50 states were considered but 11 states introducing similar interventions were excluded from the donor pool, and only 5 states ended up forming an SC. Such data may be repurposed toward identification and thus continue to play an important role as valid proxy variables. Another example of candidate proxies is measured covariates of units in the donor pool that are contemporaneous with $\left\{Y_{t},W_{Dt}\right\}$, because they do not have a causal impact on the outcomes but are associated with $\lambda_{t}$ and $X_{t}$. We highlight that Assumption 4 requires pre-determined proxies or donor units based on domain knowledge rather than data-driven selection. Empirical selection of donors and post-selection inference in SC setting is discussed in Li and Bell (2017), Chernozhukov, Wüthrich, and Zhu (2021), and Abadie (2021), while Rakshit, Shi, and Tchetgen Tchetgen (2025) considers the use of adaptive LASSO for selecting valid proxies.

Theorem 2 presents our identification and estimation strategy for the SC weights leveraging proxy variable(s)$Z_{t}$, proved in SectionB of the supplementary material.

### Theorem 2 (Moment condition for $\alpha_{D}$).

Under Assumptions 1–4, the SC weights $\alpha_{D}$ satisfies the moment condition $E\left[Y_{t}-\sum_{i\in D}\alpha_{i}\left.W_{it}|Z_{t}\right]=0\;\right.$ for any $t\leq T_{0}$.

Theorems 1 and 2 establish a connection between SC methods and the recently proposed proximal causal inference (PI) framework (Miao, Geng, and Tchetgen Tchetgen 2018; Miao et al. 2024; Tchetgen Tchetgen et al. 2024; Cui et al. 2024). Acknowledging that measured covariates are often imperfect proxies of the underlying unobserved confounding factor in practice, the PI framework leverages such proxies to identify causal effects without necessarily invoking a no-unmeasured-confounding assumption. The proposed formulation of the SC framework can be viewed as a special case of the more general PI framework with $W_{it}$ and $Z_{t}$ serving as proxies of the latent factors $\lambda_{t}$. In this sense, our estimation approach may be viewed as a generalization of the instrumental variable methods of Freyberger (2018) and Ferman and Pinto (2021) where lagged outcomes were used as instruments to correct for the measurement error.

Based on the above identification results, we propose to estimate $\alpha_{D}$ using pretreatment data with the following estimating function

(7) $$U_{t}\left(\alpha_{D}\right)=Z_{t}\left(Y_{t}-\sum_{i\in D}\alpha_{i}W_{it}\right),t=1,\ldots ,T_{0},$$

provided that $Z_{t}\in R^{d}$ with d≥|D| . More generally, one may employ any user-specified transformation $g\left(Z_{t}\right)$ of dimension at least |D|, subject to standard regularity conditions. We show that

(8) $$E\left[U_{t}\left(\alpha_{D}\right)\right]=0$$

for any $t\leq T_{0}$ in Section D of the supplementary material. The above moment equation motivates using the generalized method of moments (GMM) with $U_{t}\left(\alpha_{D}\right)$ as the identifying moment restrictions (Hansen 1982). The (constrained) GMM solves

(9) $${\hat{\alpha }}_{D}=\underset{\alpha_{D}\in W}{\arg\min}m{\left(\alpha_{D}\right)}^{⊤}\hat{\Omega }m\left(\alpha_{D}\right),$$

where $m\left(\alpha_{D}\right)=1/T_{0}\sum_{t-1}^{T_{0}}U_{t}\left(\alpha_{D}\right)$ is the pretreatment sample moments evaluated at $\alpha_{D}$, $\hat{\Omega }$ is a d×d user-specified symmetric and positive-definite weight matrix, and $W\subseteq {\hat{R}}^{\left|D\right|}$ denotes the feasibility set of SC weights, which can incorporate user-specified restrictions such as the convex hull constraint; see Remark 5 for further details. Below we present the rank condition under which (8) has a unique solution:

### Assumption 5 (Rank condition).

The d×|D| matrix $E\left[Z_{t}{W_{Dt}}^{⊤}\right]$ is of full column rank for any $t\leq T_{0}$.

It is easy to show that Assumption 5 is violated if Assumption 4 holds and $Z_{t}\;⊥\lambda_{t}$. That is, Assumption 5 implies that the proxies $Z_{t}$ are associated with latent factors $\lambda_{t}$.

### Remark 4.

As the existence of multiple solutions may complicate inference, hereafter we assume the full-rank condition which guarantees unique identification of $\alpha_{D}$. We refer the readers to Shi et al. (2020) when multiple sets of SC weights may exist in the categorical data setting. More generally, one may define additional criteria for an “optimal” set of synthetic control weights. In Section E of the supplementary material, we adapt the recent work of Zhang et al. (2023) on such a strategy in PI setting to nonparametric treatment effect estimation for the SC setting.

### Remark 5.

In SC applications, the number of donors may be comparable to the number of pretreatment periods, making it common to restrict the weight space to prevent extrapolation and improve interpretability. A standard choice is the simplex,

(10) $$W=\left\{\alpha_{D}:\alpha_{i}\geq 0,\forall i\in D\;\text{and}\sum_{i\in D}\alpha_{i}=1\right\},$$

so that $\alpha_{D}$ acts as a weighting vector reflecting the relative importance of each donor unit (Abadie, Diamond, and Hainmueller 2010; Abadie 2021; Ferman and Pinto 2021).

However, if the true SC weights lie outside the imposed feasibility set, the estimator becomes inconsistent (see Section J.2 of the supplementary material). To mitigate this issue, recent work has considered larger sets such as $W=\left\{\alpha_{D}\in {\mathbb{R}}_{+}^{\left|D\right|}:\right.\left.\sum_{i\in D}\left|\alpha_{i}\right|\leq 1\right\}$ (Chernozhukov, Wuthrich, and Zhu 2025; Chernozhukov, Wüthrich, and Zhu 2021) or $W={\mathbb{R}}^{\left|D\right|}$ (Hsiao, Ching, and Wan 2012; Doudchenko and Imbens 2016) at the cost of allowing extrapolation.

In practice, the number of pretreatment periods $T_{0}$ can often be small compared to the number of control units. In fact, when |D| is of the same order as $T_{0}$, Assumption 5 implies that d, the number of treatment proxies $Z_{t}$, is also comparable to or larger than $T_{0}$. In this case, even with weight constraints, standard GMM estimators may perform poorly, a challenge that is not present in the conventional SC literature. To address this, we additionally consider a constrained ridge-regularized estimator that employs a ridge-type weight matrix for $\hat{\Omega }$ . For analytical convenience and to facilitate theoretical derivations, we adopt a jackknife instrumental variables (JIVE) approach following Hansen and Kozbur (2014). Full details and derivations are provided in Section G of the supplementary material.

### Example 1.

As discussed above, when available, the outcome of the supplemental control unit(s) not in the donor pool, $W_{\bar{D}t}$ may be used as proxy variables, assuming conditionally independent errors $\left\{\varepsilon_{0t},\varepsilon_{Dt}\right\}\;⊥\varepsilon_{\bar{D}t}|\lambda_{t}$ for all t. Then our proposed method entails estimating $\alpha_{D}$ based on the following estimating function

$$U_{\text{PI},t}\left(\alpha_{D}\right)=W_{\bar{D}t}\left(Y_{t}-\sum_{i\in D}\alpha_{i}W_{it}\right),t=1,\ldots ,T_{0}.$$

Importantly, the residual, $Y_{t}-\sum_{i\in D}\alpha_{i}W_{it}=\varepsilon_{0t}-\sum_{i\in D}\alpha_{i}\;\varepsilon_{it}$, is independent of $W_{\bar{D}t}$ given $\lambda_{t}$ in the pretreatment period, hence, one can show that $U_{\text{PI},t}\left(\alpha_{D}\right)$ is an unbiased estimating function with mean zero. In contrast, the moment function used in the classical OLS-based SC method is the standard normal equation

$$U_{\text{OLS},t}\left(\alpha_{D}\right)=W_{Dt}\left(Y_{t}-\sum_{i\in D}\alpha_{i}W_{it}\right),t=1,\ldots ,T_{0},$$

which does not have mean zero and therefore fails to be unbiased because $W_{Dt}$ is correlated with the residual.

### 3.2. Inference of the Causal Effect on the Treated

By Theorems 1 and 2, a natural estimator for $\tau_{t}$ (and therefore also a predictor for $\theta_{t}$) is ${\hat{\tau }}_{t}={\hat{\theta }}_{t}=Y_{t}-\sum_{i\in D}{\hat{\alpha }}_{i}W_{it}$. Below we introduce three methods of uncertainty quantification, each suitable for different estimands or predictands discussed in Section 2. In particular, we use prediction intervals for random parameters and confidence intervals for fixed parameters.

#### 3.2.1. Permutation Inference Approach: Inference for $\theta_{t}$

Under the linear interactive fixed effects model, Chernozhukov, Wüthrich, and Zhu (2021) proposed a conformal permutation inference method to construct pointwise confidence intervals for the treatment effects $\theta_{t}$ (assumed to be fixed) by inverting permutation tests. They proved the non-asymptotic coverage guarantee of their method. When $\theta_{t}$ is considered random, they showed that the same method produces predictive intervals for $\theta_{t}$ with non-asymptotic coverage guarantee. Their framework is general and can be adapted to methods that produce consistent estimators of what they referred to as “mean-unbiased proxies” of $Y_{t}(0)$, that is a function of the observed data $f\left(O_{t};\zeta \right)$ indexed by some unknown parameter ζ such that $E\left[f\left(O_{t};\zeta \right)\right]=E\left[Y_{t}(0)\right]$, where *O_t_* is a random vector of observed variables at time t. Recall that by Theorem 1, $\sum_{i\in D}\alpha_{i}W_{it}$ is a mean-unbiased proxy that satisfies $E\left[Y_{t}(0)\right]=E\left[\sum_{i\in D}\alpha_{i}W_{it}\right]$ for every t≥1. Therefore, valid prediction inference for $\theta_{t}$ is possible using the method in Chernozhukov, Wüthrich, and Zhu (2021) with minor modifications. Here we summarize the implementation of their permutation inference approach to construct pointwise prediction intervals for $\theta_{t}$, $t=T_{0}+1,\ldots ,T$, where SC weights are estimated using the proximal inference approach in Section 3.1. We refer the readers to Chernozhukov, Wüthrich, and Zhu (2021) for more theoretical details and possible extensions.

To introduce the construction of the permutation *p*-value, we fix $t=T_{0}+1$ for notational convenience and consider the null hypothesis $H_{0}:\theta_{t}=\theta_{0t}$. Under $H_{0}$, the potential outcome $Y_{t}(0)=Y_{t}-\theta_{0t}$, so the augmented sample $\left\{Y_{1},\ldots ,Y_{T_{0}},Y_{t}-\theta_{0t}\right\}$ can be used to estimate the synthetic control weights $\alpha_{D}$ using the methods in Section 3.1. Residuals are then computed as $\hat{e}={\left({\hat{e}}_{1},\ldots ,{\hat{e}}_{T_{0}},{\hat{e}}_{t}\right)}^{⊤}$ where ${\hat{e}}_{s}=Y_{s}-\sum_{i\in D}{\hat{\alpha }}_{i}W_{is}$ for $s=1,\ldots ,T_{0}$ and ${\hat{e}}_{t}=Y_{t}-\theta_{0t}-\sum_{i\in D}{\hat{\alpha }}_{i}W_{it}$. Then, the permutation *p*-value is defined as

$$\begin{array}{l} \hat{p}\left(\theta_{0t}\right)=1-\hat{F}\left(\left|{\hat{e}}_{T_{0}+1}\right|\right),\;\;\text{where}\\ \hat{F}(x)=\frac{1}{T_{0}+1}\sum_{s=1}^{T_{0}+1}1\left(\left|{\hat{e}}_{s}\right|<x\right). \end{array}$$

Let $r_{t}=\varepsilon_{0t}-\sum_{i\in D}\alpha_{i}\varepsilon_{it}$. In Section D of the supplementary material, we show the asymptotic validity of the proposed test with the constrained GMM estimator when either (i) ${\left\{r_{t}\right\}}_{t=1}^{T_{0}+1}$ are iid or (ii)${\left\{r_{t}\right\}}_{t=1}^{T_{0}+1}$ are stationary with some regularity conditions.

Notably, stationarity is required only of the noise process $r_{t}$, thereby allowing the overall data-generating process to be nonstationary and to exhibit general forms of dependence.

The 1−a pointwise prediction interval for $\theta_{t}$ is constructed by inverting the above permutation test, that is, $C_{1-a}(t)=\left\{\theta_{0t}:\hat{p}\left(\theta_{0t}\right)>a\right\}$. In practice, Chernozhukov, Wüthrich, and Zhu (2021) proposed to choose a fine grid of candidate values for $\theta_{0t}$.

#### 3.2.2. SCPI Approach: Inference for $\theta_{t}$, Viewed as Random

Cattaneo, Feng, and Titiunik (2021); Cattaneo et al. (2025a) developed alternative non-asymptotic prediction intervals for $\theta_{t}$, $t=T_{0}+1,\ldots ,T$, by using probability concentration bounds. Omitting measured covariates, their framework applies to the estimation methods in which the SC weights α can be expressed as

(11) $$\hat{\alpha }=\underset{\alpha \in W}{\mathrm{argmin}}{\left(A-B\alpha \right)}^{⊤}V(A-B\alpha ),$$

where A denotes the vector of pretreatment features for the treated unit, B is the matrix of corresponding features for the control units, V is a symmetric weighting matrix, and W denotes the feasibility set of synthetic control weights. This approach decomposes the residual error into in-sample error, that is, error due to SC weights estimation using pretreatment information, and out-of-sample error, that is, stochastic error in the post-treatment outcomes. Probability concentration bounds are then derived separately for each component, which further produces the prediction interval for the potential outcomes $Y_{t}(0)$,$t=T_{0}+1,\ldots ,T$. Cattaneo, Feng, and Titiunik (2021) and Cattaneo et al. (2025a) proposed several extensions of their approach, including inference for multiple treated units and staggered treatments. In particular, Cattaneo et al. (2025a) discussed prediction intervals for the average post-treatment effect ${\bar{\theta }}^{\text{r}}=\sum_{t=T_{0}+1}^{T}\theta_{t}/\left(T-T_{0}\right)$ and implemented their methods in the R package *scpi*.

Interestingly, the proposed PI estimator of SC weights in (9) is equivalent to (11) by setting $A={\left(Y_{1},\ldots ,Y_{T_{0}}\right)}^{⊤}$, $B={\left(W_{D1},\ldots ,W_{DT_{0}}\right)}^{⊤}$ and

$$V=\frac{1}{T_{0}}\left(Z_{1},\ldots ,Z_{T_{0}}\right)\hat{\text{Ω}}{\left(Z_{1},\ldots ,Z_{T_{0}}\right)}^{⊤},$$

which suggests that their predictive inference framework can provide uncertainty quantification of our proposed PI method with straightforward modification.

#### 3.2.3. Estimation and Inference for the Expected Effects $\tau_{t}$

Consider the setting where both numbers of pre- and post-treatment periods are large and diverge at the same rate, that is, $T_{0}$, $T_{1}\to \infty$ and $T_{0}/T_{1}\to \rho \in (0,\infty )$ as T→∞, where ρ is a fixed constant. A possible estimand of interest is the limit of the expected average post-treatment effect $\bar{\tau }=\lim_{T\to \infty }\sum_{t=T_{0}+1}^{T}\tau_{t}/\left(T-T_{0}\right)$. More generally, it is possible to pool information across time via some form of smoothing to infer a deterministic trend, for example, a parametric model encoding smooth dependence on t. For example, when appropriate, one may assume that $\tau_{t}=\tau (t/T;\gamma )$ is a function of time indexed by a finite-dimensional parameter $\gamma \in R^{k}$, such as $\tau_{t}=\gamma_{0}+\gamma_{1}t/T$. Going beyond parametric models, $\tau_{t}$ can even be estimated flexibly using standard time series analysis. To see this, we define the following post-treatment contrast for $t>T_{0}$ as $e_{t}=Y_{t}-\sum_{i\in D}\alpha_{i}W_{it}=\tau_{t}+r$, which may be viewed as a standard time series where $\tau_{t}$ captures deterministic trends over time, including potential secular and seasonal patterns, and $r_{t}=\varepsilon_{0t}-\sum_{i\in D}\alpha_{i}\varepsilon_{it}$ is a mean zero residual process.

Given a consistent estimator ${\hat{\alpha }}_{D}$ based on pretreatment data using the methods described in Section 3.1, one can estimate $e_{t}$ with ${\hat{e}}_{t}=Y_{t}-\sum_{i\in D}{\hat{\alpha }}_{i}W_{it}$ in the post-treatment period, which can in turn be analyzed as time series data. For example, one may first inspect the estimated time series ${\hat{e}}_{t}$ to determine an appropriate functional form for τ(t/T;γ), which may then be used to estimate γ by standard regression techniques. Alternatively, one may estimate $\tau_{t}$ by standard time series smoothing techniques such as moving averages. When both secular and seasonal patterns exist, one may estimate both components, say using an autoregressive–moving-average (ARMA) model or possible generalizations of the latter with the goal of producing stationary residuals, a process sometimes referred to as pre-whitening a time series. Nonparametric regression methods such as series estimation (e.g., smoothing splines, polynomials, wavelets) for estimation of $\tau_{t}$ have also been proposed (Dong and Linton 2018). We refer the readers to the monograph Hamilton (1994) for a more detailed exposition of such techniques. Effect estimation by viewing $e_{t}$ as a time series was also considered by Abadie and Gardeazabal (2003), where a smooth treatment effect curve was fit using moving averages of ${\hat{e}}_{t}$ to inspect the economic costs of terrorist activities for the Basque Country.

To illustrate, we focus on inference for the limiting expected average post-treatment effect $\bar{\tau }$, which may be estimated by

(12) $$\hat{\bar{\tau }}=\frac{1}{T-T_{0}}\sum_{t=T_{0}+1}^{T}\left(Y_{t}-\sum_{i\in D}{\hat{\alpha }}_{i}W_{it}\right)$$

We note that the two-stage estimation of $\alpha_{D}$ using pretreatment data followed by $\bar{\tau }$ using post-treatment data can be achieved by stacking (7) and (12) into a combined estimating function for simultaneous estimation of both parameters. Let $D_{t}={\left\{{W_{Dt}}^{⊤},1\left(t>T_{0}\right)\right\}}^{⊤}\in R^{|D|+1}$, $V_{t}=\left\{1\left(t\leq T_{0}\right)Z_{t}^{⊤},1(t>\right.{\left.\left.T_{0}\right)\right\}}^{⊤}\in R^{d+1}$, and $\xi ={\left({\alpha_{D}}^{⊤},\bar{\tau }\right)}^{⊤}$, then ${\tilde{U}}_{t}(\xi )=V_{t}^{⊤}\left(Y_{t}-D_{t}^{⊤}\xi \right)$.

That is,

(13) $$\begin{array}{l} {\tilde{U}}_{t}(\xi )=\left(\begin{array}{c} 1\left(t\leq T_{0}\right)U_{t}\left(\alpha_{D}\right)\\ 1\left(t>T_{0}\right)\left(Y_{t}-D_{t}^{⊤}\xi \right) \end{array}\right)\\ \;\;\;\;\;\;\;=\left(\begin{array}{c} 1\left(t\leq T_{0}\right)Z_{t}\\ 1\left(t>T_{0}\right) \end{array}\right)\left(Y_{t}-1\left(t>T_{0}\right)\bar{\tau }-\sum_{i\in D}\alpha_{i}W_{it}\right). \end{array}$$

We show that $E\left[{\tilde{U}}_{t}(\xi )\right]=0$ in Section D of the supplementary material. Then the two-stage estimation of $\alpha_{D}$ followed by $\bar{\tau }$ can be equivalently achieved as

$$\hat{\xi }=\arg\underset{\xi }{\min}\tilde{m}{\left(\xi \right)}^{⊤}\tilde{\Omega }\tilde{m}(\xi ),$$

We $\tilde{m}(\xi )=1/T\sum_{t=1}^{T}{\tilde{U}}_{t}(\xi )=1/T\sum_{t=1}^{T}V_{t}\left(Y_{t}-D_{t}^{⊤}\xi \right)\;$ and $\tilde{\text{Ω}}$ is a (d+1)×(d+1) weighting matrix whose upper-left submatrix equals $\hat{\text{Ω}}$. Using the results of Pötscher and Prucha (1997), and under Assumptions 1–5 together with regularity conditions D.2–D.10 listed in Section D.2 of the supplementary material, the resulting estimator $\hat{\xi }$ is consistent and asymptotically normal; that is, $\sqrt{T}(\hat{\xi }-\xi )\overset{d\text{​}}{\to }N(0,\text{Σ})$ as T→∞ for some positive definite matrix *:Σ*. In particular, we require only that the data be approximable by an *α*-mixing process (Doukhan 1994), which accommodates nonstationarity. Section D.2 of the supplementary material provides formal definitions of *α*-mixing and $L_{0}$-approximability, along with a detailed discussion of the corresponding regularity conditions, the explicit form of Σ, and a consistent estimator for it.

### 3.3. Nonparametric Identification and Estimation

The PI framework is far more general than the interactive fixed effects model thus far highlighted. It allows for nonparametric identification of causal effects in the sense that one can uniquely express the causal effect as a function of the observed data distribution without necessarily assuming a linear model (Miao et al. 2024). In this section, we establish conditions for such nonparametric identification for SC. Our framework allows for nonlinear models that can be applied to for example, binary or count outcomes, which are currently under-studied in the SC literature.

We first extend previous Assumptions 2–3. In order to establish nonparametric identification we require the no interference assumption previously discussed in (2):

### *Assumption 2*′ (No interference).

$W_{it}(1)=W_{it}(0)$ for any i and t.

We further extend Assumption 3 to the following assumption that the mechanism by which latent factors affect the potential outcome $Y_{t}(0)$ can be adequately captured by an unknown function of a sufficiently rich set of proxies $W_{Dt}(0)$ in the absence of treatment.

### *Assumption 3*′ (Existence of confounding bridge).

There exists a function $h\left(W_{Dt}\right)$ such that the outcome model for $Y_{t}(0)$ is equivalent to a model for $h\left(W_{Dt}(0)\right)$:

(14) $$E\left[Y_{t}(0)|\lambda_{t}\right]=E\left[h\left(W_{Dt}(0)\right)|\lambda_{t}\right],\;\forall t\geq 1.$$

Assumption 3′ states that in the absence of treatment, the effect of $\lambda_{t}$ on the treated unit can be uncovered by the effect of $\lambda_{t}$ on the donor units encoded by an unknown function h(⋅), referred to as a confounding bridge function in the PI literature (Miao et al. 2024; Tchetgen Tchetgen et al. 2024). We show the existence of h(⋅) under sufficient conditions in Section F of the supplementary material. Below we provide two examples of confounding bridge functions under linear and nonlinear models.

### Example 2 (Classical setting).

Assumption 31 is a generalization of Assumptions 2–3 to the nonparametric setting. Specifically, Assumptions 2–3 imply

$$E\left[Y_{t}(0)|\lambda_{t}\right]=E\left[\sum_{i\in D}\alpha_{i}W_{it}(0)|\lambda_{t}\right],$$

which is a special case of (14) with $h\left(W_{Dt}(0)\right)=\sum_{i\in D}\alpha_{i}W_{it}(0)$. In this case, identifying the confounding bridge function reduces to identifying the SC weights $\alpha_{D}$.

### Example 3 (Count outcome).

For count outcomes, it is more natural to assume a multiplicative model, for example, $E\left[Y_{t}(0)|\right.\left.\lambda_{t}\right]=\exp\left[\mu_{0}\lambda_{t}\right]$ for any t. Then Assumption 3′ holds with $h\left(W_{Dt}(0)\right)=\exp\left[f\left(W_{Dt}(0)\right)\right]$ if f satisfies $E\left[\exp\left[f\left(W_{Dt}(0)\right)\right]|\right.\left.\lambda_{t}\right]=\exp\left[\mu_{0}\lambda_{t}\right]$ for all t. In particular, if f(⋅) is linear in $W_{it}(0)$, then this condition involves the moment generating function and may hold for Normal and Poisson distributed outcomes.

Because $Y_{t}(1)$ is observed in the post-treatment period, one only needs to identify $E\left[Y_{t}(0)\right]$ to obtain the ETT at time $t>T_{0}$. We have the following nonparametric identification result, which is proved in Section H of the supplementary material.

### Theorem 3 (Nonparametric identification of ETT).

Under Assumptions 1, 2′, and 3′, we have $E\left[Y_{t}(0)\right]=E\left[h\left(W_{Dt}\right)\right]$ for any t, and therefore the ETT at time t for any $t>T_{0}$ is $\tau_{t}=E\left[Y_{t}-h\left(W_{Dt}\right)\right]$.

One cannot directly identify h(⋅) from (14) because $\lambda_{t}$ is unobserved. Nevertheless, when supplemental proxy variables $Z_{t}$ satisfying Assumption 4 are available, h(⋅) can be inferred from observed data, as stated in Theorem 4 below and proved in Section H of the supplementary material.

### Theorem 4 (Moment condition for h(⋅)).

Under Assumptions 1, 3′, and 4, for any $t\leq T_{0}$, the confounding bridge function h(⋅) satisfies the moment condition

(15) $$E\left[Y_{t}-h\left(W_{Dt}\right)|Z_{t}\right]=0.$$

Theorems 1 and 2 are a special case of Theorems 3 and 4 under more stringent model assumptions, that is, Assumptions 2-3. In general, there may exist multiple solutions to (15). In Section I of the supplementary material, we show under additional assumptions that (i) the distribution of $\lambda_{t}$ is complete with respect to $Z_{t}$ and that (ii) the conditional distribution $\left(Y_{t}(0),W_{t}(0)\right)\;|\;\lambda_{t}$ is stationary, any solution to (15) is a confounding bridge function that satisfies (14). As such, multiple confounding bridge functions might exist and lead to the same $E\left[Y_{t}(0)\right]$ . In the presence of multiple solutions of (15), we present a nonparametric series estimator of the confounding bridge function and the ETT in Section E of the supplementary material, based on recent work by a subset of the authors (Zhang et al. 2023). On the other hand, the confounding bridge function can be uniquely identified if the distribution of $W_{Dt}$ is complete with respect to $Z_{t}$, which we also establish in Section I of the supplementary material. Unique identification of h significantly simplifies estimation and inference. Suppose a suitable parametric model $h\left(W_{Dt};\alpha \right)$ is posited for the confounding bridge function, where α is a finite-dimensional indexing parameter. Then, identification of *α* can be expressed as the more standard condition: $E\left[h\left(W_{Dt};\alpha \right)-h\left(W_{Dt};\alpha^{\prime }\right)|Z_{t}\right]\neq 0$ with a positive probability for any $\alpha \neq \alpha^{\prime }$ and $t\leq T_{0}$.

Similar to Section 3.2, having identified $h\left(W_{Dt}\right)$, the residual process $e_{t}=Y_{t}-h\left(W_{Dt}\right)=\tau_{t}+r_{t}\left(t=T_{0}+1,\ldots ,T\right)$ may again be viewed as a standard time series where $\tau_{t}=\tau (t/T)$ is an unknown smooth function of t/T∈(0,1] that captures deterministic time trends, and $r_{t}=Y_{t}(0)-h\left(W_{Dt}\right)$ is a mean zero error term by Theorem 3. Methods in Section 3.2.3 can still be applied for effect estimation with simple modification. It is unclear how the conformal inference and prediction inference approaches in Sections 3.2.1 and 3.2.2 can be extended to the nonparametric setting.

## 4. Simulation

We investigate the finite sample performance of our proposed method under various conditions. We simulate time series data on *N* control units and one treated unit over $T_{0}=80$, 140, or 200 time period pretreatment and the same time length post-treatment, that is,$T_{0}=T_{1}$. The data generating mechanism follows Assumptions 1 and 2 with the random errors $\varepsilon_{it}\overset{\text{iid}}{\sim }N\left(0,1.5^{2}\right)$. We let $\theta_{t}=2$ for all t. We simulate a vector of latent factors $\lambda_{t}={\left(\lambda_{t1},\ldots ,\lambda_{tr}\right)}^{⊤}$, where $\lambda_{tk}$’s are stationary and independently sampled from $N\left(0.5,0.5^{2}\right)$ distribution, k=1,…,r, and r=2, 3, or 5. That is, we generate three settings with two, three, or five latent factors. For each setting, we assume the number of control units N=2r, and the first half of the control units (i=1,…,r) constitute the donor pool with |D|=r. We specify factor loadings $\mu_{i}$, i=0,…,N as follows. For the treated unit, the factor loading is $\mu_{0}={\left(1,\ldots ,1\right)}^{⊤}$. For the control units, the first half of the control units (i=1,…,r) have factor loadings $\mu_{i}$ and corresponding SC weights $\alpha_{D}$ satisfying Assumption 3 as follows......


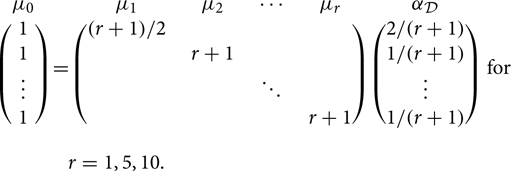



Notably, the SC weights $\alpha_{D}$ sum to one. The second half of the control units (i=r+1,…,N) have identical factor loadings as the first half. That is, $\mu_{r+k}=\mu_{k}$, for k=1,…,r.

We first focus on the point estimators of $\bar{\tau }$ . We implement our proposed method taking the first half of control units as donors $W_{Dt}$ and second half of control units as supplemental proxies $W_{\bar{D}t}$. We consider both unconstrained proximal inference (PI) method, or the constrained proximal inference (cPI) method where $\alpha_{D}$’s are all positive and sum to one. For comparison, we implement both the constrained OLS proposed in Abadie, Diamond, and Hainmueller (2010) and the unconstrained OLS, taking all N control units as donors. For the unconstrained OLS method, we fit the following linear regression model to estimate the SC weights using pretreatment data:$Y_{t}=\sum_{i=1}^{N}{\tilde{\alpha }}_{i}W_{it}+v_{it}$, *$t=1,\ldots ,T_{0}$* where $v_{it}$ is some random error, and estimate $\bar{\tau }$ using the average post-treatment contrast in (12). This is equivalent to the classical SC method without the constraint that ${\tilde{\alpha }}_{i}\geq 0$, i∈D and $\sum_{i=1}^{N}{\tilde{\alpha }}_{i}=1\;$. For the constrained OLS method (referred to as SC), we use the scpi package (Catteneo et al. 2025b), which employs conic optimization to remarkably speed up the estimation (Boyd and Vandenberghe 2004). We extract the estimated SC weights to predict $Y_{t}(0)$ in the post-treatment period and then estimate $\bar{\tau }$ . Simulation results are summarized over 5000 Monte Carlo samples.

**Figure 2.**
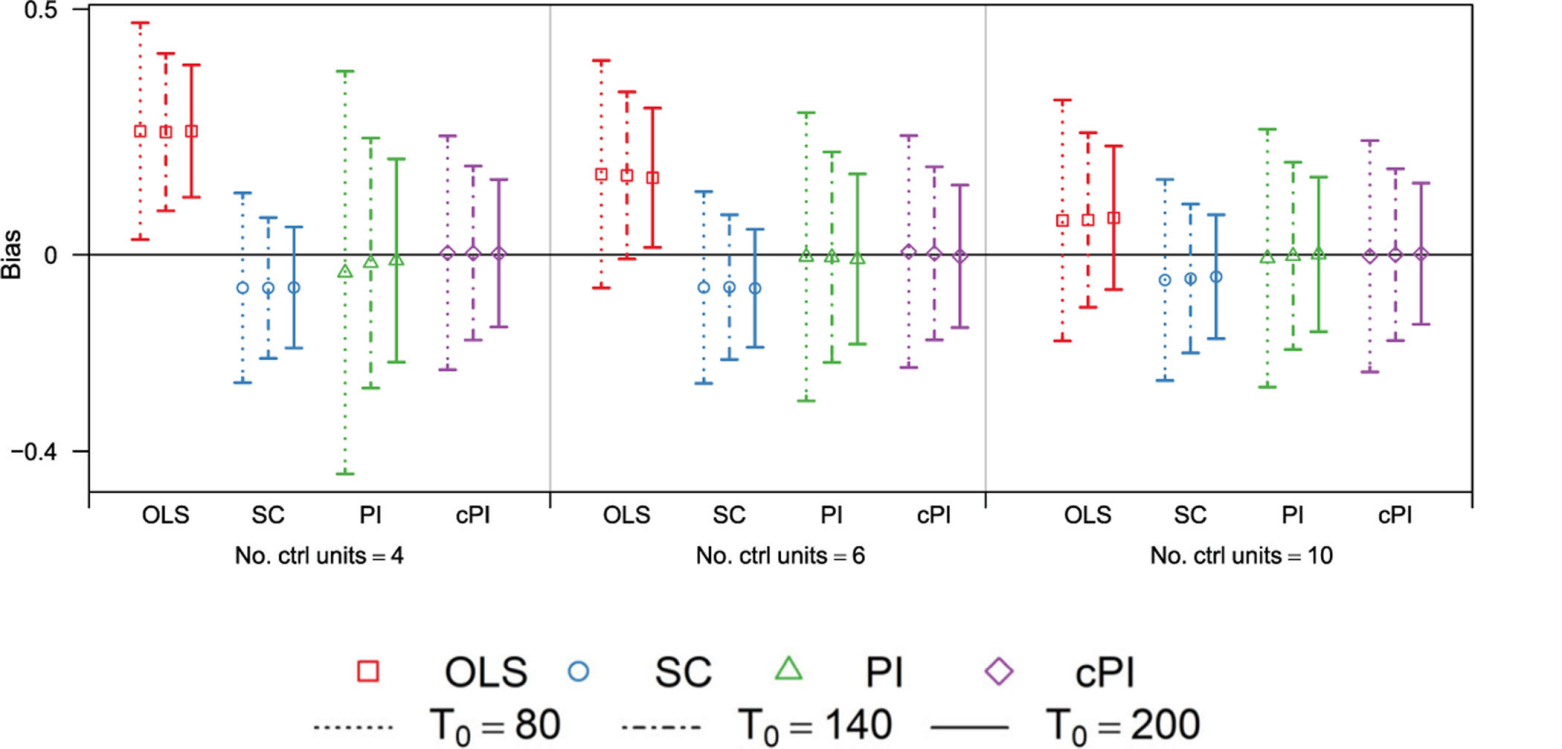
Bias ± standard deviation of τ estimates based on the unconstrained (OLS) and constrained (SC) regression methods, and our proposed unconstrained (PI) and constrained (cPI) proximal inference methods, with a range of number of control units N=4, 6, or 10 and pre- and post-treatment time period $T_{0}=T_{1}=80$, 140, or 200.

Figure 2 presents bias of the $\bar{\tau }$ estimates based on the four methods described above (OLS, SC, PI, and cPI). As expected from theory, the bias of PI and cPI approaches decreases with increasing $T_{0}\;$ and $T_{1}$. In contrast, the unconstrained OLS regression estimates were substantially biased. The SC estimates are subject to a smaller bias, which does not vanish with increased time periods. This is also consistent with our theoretical expectation, as pointed out in Section 2, that the regression based estimator solves an estimating equation that is biased, and thus does not consistently estimate $\bar{\tau }$. We also note that such a bias does not decrease as the number of control units increases. This is because, as discussed in Ferman (2021), OLS-based weight estimate ${\hat{\alpha }}_{D}$ converges to weights that attempt to minimize the variance of $\sum_{i\in D}\alpha_{i}\varepsilon_{it}$, which does not vanish in our setting as $\alpha_{D}$ does not decrease with increasing N.

We conduct additional simulation studies to study the performance of the proposed methods under various settings. As in Ferman and Pinto (2021), we consider a setting where the means of latent factors change over time in Section J.1 of the supplementary material. In this case, both OLS and SC point estimators are subject to a noticeable bias that does not vanish with increasing $T_{0}\;$, but PI and cPI still produce unbiased estimators, suggesting that the proposed methods are not impacted by the mean shift of the latent factors. When the latent factor loading is constructed in a way that the true SC weights do not sum up to one, only the unconstrained PI method produces unbiased point estimators (Supplementary Figure S.2). Under weakly dependent residual error settings where $\varepsilon_{it}$ is an AR(1) process, the results are similar to those reported above (Section J.3 of the supplementary material). We repeat the above simulation studies with $T_{0}=30$ in Section J.4 of the supplementary material. Although the median bias of PI and cPI methods remains small, the two methods may produce unstable estimates, resulting in large mean bias and standard deviation. To evaluate the impact of selection of the donor and proxy units, we compare the PI and cPI methods where the donor units are either pre-determined as described before or randomly selected among the control units. Both methods produce severely biased estimates if the donor units are randomly selected (Section J.5 of the supplementary material). We also considered a setting with a univariate measured covariate and implemented the extension of our methods described in Section C of the supplementary material. Our proposed method successfully adjusts for measured covariates with similar bias but higher efficiency as indicated by a narrower Monte Carlo CI compared to the PI method without covariate adjustment (Section J.6 of the supplementary material). We further study a time-varying treatment effect setting with $\theta_{t}=\gamma_{0}+\gamma_{1}t/T$ where $\gamma ={\left(1,1\right)}^{\text{T}}$. The results are similar to the above and are presented in Section J.7 of the supplementary material. Finally, we investigate a setting where outcomes are Poisson-distributed count variables, a setting which, to the best of our knowledge, has not been extensively studied in the SC literature. As shown in Section J.8 of the supplementary material, the bias of the PI approach again decreased with increasing sample size, whereas a standard log-linear generalized linear model produces biased effect estimates.

The reported bias of constrained OLS in our simulation study is in line with the warning given by Abadie, Diamond, and Hainmueller (2010), Abadie (2021), and Abadie and Vives-i Bastida (2022), who recommend the use of the constrained OLS estimator only when estimated weights can produce synthetic controls that are a good match for the treated unit. Note that we did not evaluate the fit in advance in our simulation and found that the results are a good illustration of their warning.

In addition, we perform simulation studies to evaluate different prediction intervals for $\theta_{T_{0}+1}$ using the setting described before, with stationary latent factors and positive SC weights that sum up to one. We construct 90% prediction intervals using the permutation inference approach in Section 3.2.1 or the SCPI approach in Section 3.2.2 for the OLS estimator or the proximal inference approach, with or without the simplex constraint of SC weights, which results in eight different types of prediction intervals. For the SCPI approach, sub-Gaussian bounds were used for the out-of-sample error. From Table 1, the SCPI approach produces prediction intervals with over-coverage, whereas the permutation inference approach produces calibrated prediction intervals, except for the ones based on the constrained OLS regression, which have slight under-coverage. The results are similar when the latent factors are nonstationary or the SC weights do not sum to one (Section J.9 of the supplementary material), which surprisingly also holds for prediction intervals based on constrained OLS or proximal inference, suggesting that both approaches of predictive inference are conservative. The over-coverage of the SCPI prediction intervals is partly because $\theta_{t}$ is a fixed constant in our simulation, different from the intended setting of the SCPI approach, where $\theta_{t}$ is random.

We further evaluate different methods for inference of post-treatment average effects: the SCPI methods for prediction inter-vals of $\bar{\theta }$ in Section 3.2.2 with subgaussian bounds for the out-of-sample error, and the GMM method for confidence intervals of $\bar{\tau }$, where the SC weights are estimated by unconstrained OLS or the proximal inference method. We present the results in Table 2. Similar to the case for pointwise prediction intervals, the SCPI approach is conservative for the average post-treatment effect for all methods. For the GMM approach, the confidence intervals of the OLS method have severe under-coverage, while those of the PI method are well-calibrated.

## 5. Application: the 1990 German reunification

We illustrate the use of our proposed PI method by applying it to a comparative case study of the 1990 German reunification. Abadie, Diamond, and Hainmueller (2015) studied the effect of the German reunification on per-capita GDP in West Germany using the regression-based SC method with the additional restriction that the weights are nonnegative and normalized to one. They collected annual country-level panel data in 1960–2003 for both West Germany, the treated unit, and 16 untreated Organisation for Economic Co-operation and Development (OECD) countries. Data and code to replicate results in Abadie, Diamond, and Hainmueller (2015) are available online (Hainmueller 2014). Cattaneo et al. (2025b) re-analyzed the data to demonstrate their proposed SCPI approach of predictive inference for treatment effects.

The outcome of West Germany, $Y_{t}$, and the control countries, *W_it_*, is the annual per-capita GDP measured in U.S. dollars (USD) in country i at time i=1,…,16,t=1,…,44. Among the 16 control countries, six (Austria, Italy, Japan, Netherlands, Switzerland, USA) were eventually given nonzero weights in Cattaneo et al. (2025b) to construct a synthetic West Germany, while the other 10 states were excluded with zero weights. We hence take outcome trajectories of the six countries as $W_{Dt}$ and leverage the rest of the countries as supplemental proxies, that is, $Z_{t}=W_{{\bar{D}}_{t}}$. For ease of demonstration, we did not include additional covariates as considered in Abadie, Diamond, and Hainmueller (2015). We used the four methods of estimating SC weights compared in Section 4 to compute the synthetic West Germany that aims to replicate the GDP that would have evolved without the reunification.

**Figure 3.**
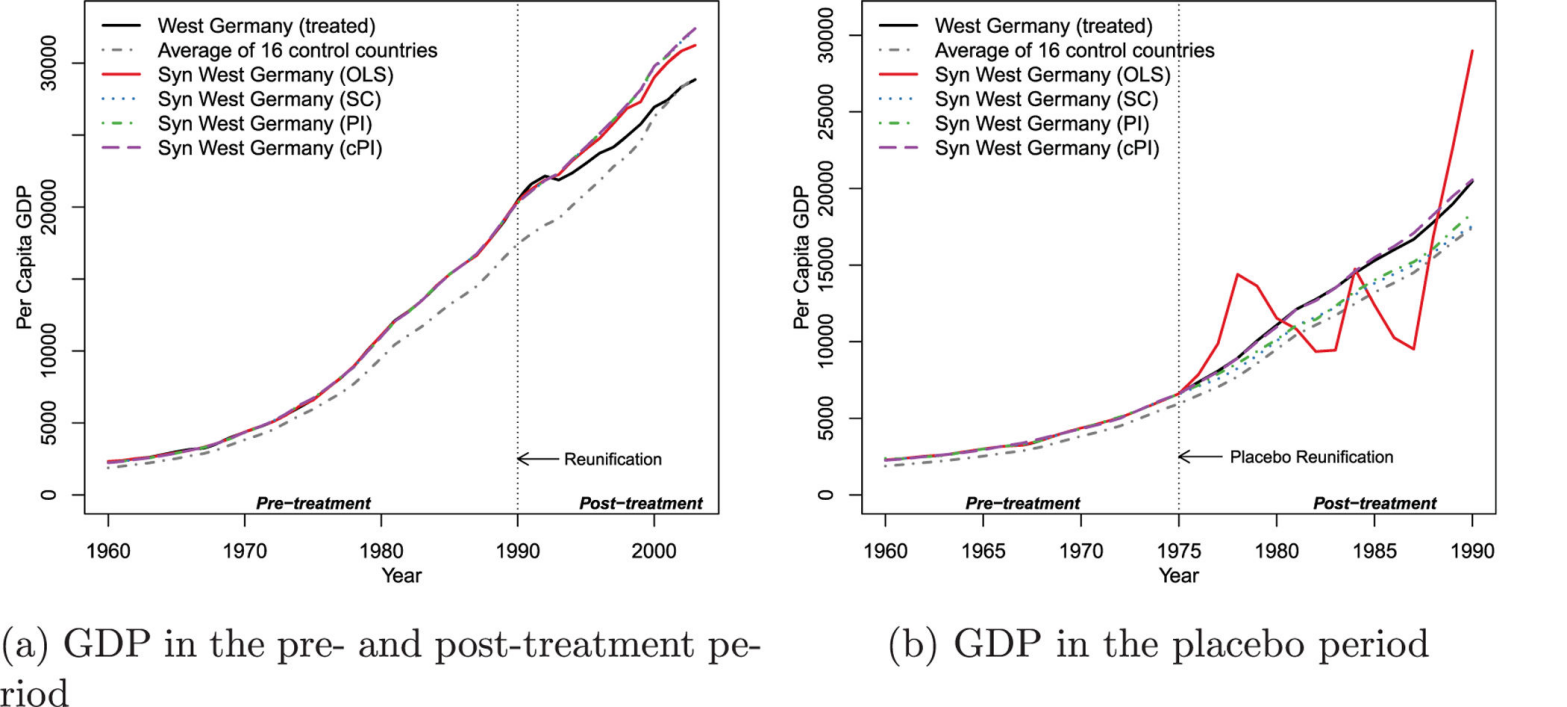
Trajectory in per-capita GDP in West Germany and synthetic West Germany. OLS: unconstrained OLS regression; SC: regression-based method with the additional simplex constraint of the SC weights; PI: proximal inference method; cPI: proximal inference method where the weights satisfy the simplex constraint.

Figure 3(a) presents the trends of per-capita GDP in West Germany and synthetic West Germany based on OLS regression or the proposed proximal inference method, with or without the restriction that the weights are nonnegative and sum to one. In the pretreatment period, the difference in per-capita GDP comparing West Germany and its synthetic counterpart is close to zero from all methods except for the simple average. After 1990 the trends of per-capita GDP diverged.

The OLS, SC, PI and cPI point estimates and 90% prediction interval by the SCPI approach of the average post-reunification treatment effect are −1323 USD (−6589 USD, 3029 USD), −1668 USD (−3462 USD, 16 USD), −1709 USD (the SCPI algorithm does not converge), and −1719 USD (−3669 USD, −610 USD). The GMM 90% confidence intervals for the expected average post-reunification treatment effect using HAC standard error estimates are (−2127 USD, −520 USD) for the OLS method, and (−2806 USD, −616 USD) for the PI method, suggesting a negative impact of reunification on West Germany’s GDP on average.

We also demonstrate the permutation inference approach to construct pointwise prediction intervals for $\theta_{t}=Y_{t}(1)-Y_{t}(0)$ using the SC method and cPI method, the two methods with the simplex constraint to avoid extrapolation. We did not show the results for the prediction intervals of the SCPI approach as they tend to be much wider than the permutation prediction intervals and suffer from numerical failure in the optimization. We present the results in Section K of the supplementary material.

To validate the results, we further conducted a falsification study by restricting estimation to the pretreatment period, during which the causal effect is expected to be null. We artificially define a placebo-reunification time of 1975, which falls in the middle of the pretreatment period. Figure 3(b) presents the per-capita GDP trajectories in West Germany and synthetic West Germany computed using the placebo period. Post-1975, the synthetic West Germany by unconstrained OLS notably deviated from the observed per-capita GDP of West Germany, while the synthetic West Germany by the constrained proximal inference method produced the best fit. The mean absolute prediction errors post 1975 for the four methods are 3316 USD for OLS, 1350 USD for SC, 1115 USD for PI, and 164 for cPI, respectively. Note that in this section, we only compare the unadjusted SC methods. Abadie, Diamond, and Hainmueller (2015) implemented an adjusted SC method for additional covariates, of which the post-1975 fit in the falsification test is comparable to the cPI method in Figure 3(b).

## 6. Discussion

In this article, we have proposed a new framework for evaluating the impact of an intervention when time series data on a single treated unit and multiple untreated units are observed, in pre- and post-treatment periods. Our proposal is motivated by recent work on proximal causal inference (Miao et al. 2024; Tchetgen Tchetgen et al. 2024; Cui et al. 2024), which is closely related to negative control methods recently developed for analysis of observational data subject to potential unmeasured confounding bias (Lipsitch, Tchetgen Tchetgen, and Cohen 2010; Shi et al. 2020; Shi, Miao and Tchetgen 2020). Our proposed framework complements traditional SC methods in settings with imperfect match of the treated units’ and donor units’ characteristics, and formalizes estimation and inference of the treatment effects on the treated unit. We further extend the traditional linear interactive effect model to more general cases such as nonlinear models allowing for binary and count outcomes which are rarely studied in the SC literature (Qiu et al. 2024).

Our work is related and complementary to the instrumental variable approach to correct for measurement error (Griliches 1977; Chamberlain and Griliches 1977; Schennach 2016). For example, Freyberger (2018) and Ferman and Pinto (2021) have considered leveraging lagged outcomes as instrumental variables. Under the linear model, our estimation approach is analogous to the above instrumental variable methods, and our nonparametric identification results generalize these previous methods. Interactive fixed effects model-based methods, such as Gobillon and Magnac (2016) presents another useful approach for latent bias adjustment in treatment effect estimation with many treated and untreated units. Compared with the above approaches, the framing, assumptions, and statistical methodology of our method have several key distinctions, which we detailed in Section L of the supplementary material.

Our methods can conveniently be extended to accommodate multiple treated units (Abadie and L’hour 2021; Ben-Michael, Feller, and Rothstein 2022). The simplest approach might be to do separate PI analyses for each treated unit, allowing for different confounding bridge functions and possibly different donor pools. Then unit-specific treatment effects could be aggregated in a meta-analytic fashion either by assuming, when appropriate, a shared common effect by inverse variance weighted estimation or by allowing for heterogeneous unit-specific effects pooled via the random effect approach.

There are several limitations. First, in our analysis of German Reunification data, we didn’t adjust for measured predictors of GDP as was done in Abadie, Diamond, and Hainmueller (2015). As these measured covariates may also be subject to the impact of reunification, adjusting for them may cause post-treatment adjustment bias and alter the interpretation of estimated effects. In such cases, methods in mediation analysis may be adapted to estimate an alternative causal effect of interest, such as direct effects (Acharya, Blackwell, and Sen 2016; Dukes, Shpitser, and Tchetgen Tchetgen 2023). Second, in Section 5, we selected proxy units as those control units with zero weights, which were estimated in a standard SC analysis as performed in Abadie, Diamond, and Hainmueller (2015) based on information on all units in the donor pool. However, such outcome-dependent preliminary proxy and control selection may introduce endogeneity, which may limit the interpretation of the treatment effects and invalidate consistency. Future research is needed to develop more formal proxy selection methods and post-selection inference for data-driven selection of either the donor pool or proxies (Park, Richardson, and Tchetgen Tchetgen 2024; Park and Tchetgen 2025; Rakshit, Shi, and Tchetgen Tchetgen 2025). Third, as demonstrated in Section J.4 of the supplementary material, when the number of pretreatment periods $T_{0}\;$ is small, the proposed methods may become unstable. In this scenario, we introduced a jackknife instrumental variables (JIVE) approach in Section G of the supplementary material for improved estimation. We leave the evaluation of these extensions to future research.

## Supplementary Material

Supp 1

Supplementary Materials

The Supplementary Material contains proofs and derivations of all technical results presented in the article and further discussions.

Supplementary materials for this article are available online. Please go to www.tandfonline.com/r/JASA.

## Figures and Tables

**Table 1.** Coverage (Average length) of 90% prediction intervals over 5000 Monte Carlo samples for $\theta T_{0+1}$ using the permutation inference approach or SCPI approach in Sections 3.2.1 and 3.2.2.

No. ctrl	$T_{0}$	Permutation Inference				SCPI			
		OLS	SC	PI	cPI	OLS	SC	PI	cPI
5	80	90.1% (5.5)	88.6% (5.5)	89.5% (7.3)	89.1% (6.3)	96.5% (7.7)	93.3% (6.5)	98.3% (11.7)	91.2% (6.4)
	140	89.6% (5.5)	89.2% (5.5)	90.5% (6.8)	90.0% (6.3)	95.4% (6.9)	92.8% (6.1)	97.8% (9.6)	91.8% (6.2)
	200	90.3% (5.4)	89.2% (5.5)	89.9% (6.6)	89.5% (6.2)	94.4% (6.5)	92.6% (6.0)	97.0% (8.7)	91.6% (6.0)
7	80	90.3% (5.6)	87.3% (5.3)	89.7% (6.5)	88.6% (6.0)	97.9% (8.8)	93.1% (6.6)	98.5% (11.6)	94.1% (7.2)
	140	89.9% (5.4)	88.5% (5.3)	90.5% (6.2)	89.9% (5.9)	97.4% (7.8)	93.8% (6.4)	98.2% (9.5)	94.3% (6.8)
	200	89.4% (5.4)	88.7% (5.3)	90.1% (6.0)	89.9% (5.8)	96.5% (7.3)	93.6% (6.3)	97.5% (8.6)	93.6% (6.6)
11	80	90.3% (5.6)	87.5% (5.0)	90.4% (5.9)	88.8% (5.5)	99.5% (10.9)	92.7% (6.6)	99.5% (12.3)	95.7% (8.0)
	140	89.6% (5.4)	87.5% (5.1)	90.4% (5.7)	89.1% (5.5)	98.9% (9.3)	94.2% (6.6)	99.1% (10.1)	96.4% (7.7)
	200	89.8% (5.3)	88.2% (5.1)	89.6% (5.6)	89.2% (5.5)	98.4% (8.5)	94.8% (6.6)	98.6% (9.1)	96.6% (7.4)

**Table 2.** Coverage (Average length) of 90% prediction intervals for $\bar{\theta }$ using the SCPI methods and 90% confidence intervals for $\bar{\tau }$ using the GMM approach over 5000 Monte Carlo samples.

90% prediction intervals for $\bar{\theta }$									
No. ctrl	4			6			10		
$T_{0}\;$	80	140	200	80	140	200	80	140	200
SCPI (OLS)	100% (2)	100% (1.5)	99.9% (1.2)	100% (2.3)	100% (1.7)	100% (1.4)	100% (2.8)	100% (2)	100% (1.7)
SCPI (SC)	96.2% (1.2)	97.2% (0.9)	97.7% (0.8)	96.1% (1.3)	97.8% (1)	98.8% (0.9)	94.7% (1.3)	97.7% (1.1)	98.9% (1)
SCPI (PI)	100% (3)	100% (2)	100% (1.7)	100% (2.7)	100% (1.9)	100% (1.6)	100% (2.7)	100% (1.9)	100% (1.5)
SCPI (cPI)	94.5% (1.3)	96.8% (1.1)	97.3% (0.9)	97% (1.6)	98.4% (1.2)	99.3% (1.1)	96.8% (1.7)	99.1% (1.4)	99.8% (1.2)
90% confidence intervals for $\bar{\tau }$									
No. ctrl	4			6			10		
$T_{0}\;$	80	140	200	80	140	200	80	140	200
GMM (OLS)	69.0% (0.7)	55.9% (0.6)	43.9% (0.5)	81.3% (0.8)	76.2% (0.6)	71.1% (0.5)	87.5% (0.8)	87.3% (0.6)	85.9% (0.5)
GMM (PIs)	92.7% (1.6)	92.3% (0.9)	91.5% (0.7)	91.4% (1.1)	91.0% (0.7)	90.8% (0.6)	90.3% (0.9)	90.5% (0.7)	90.5% (0.5)
