# Supplementary material for: Theory for Identification and Inference with Synthetic Controls: A Proximal Causal Inference Framework
Source: J Am Stat Assoc. Author manuscript; Available in PMC 2026 Jul 21. (PMC13384440; doi:10.1080/01621459.2026.2639734)
Supplement: Supp 1 [file NIHMS2185023-supplement-Supp_1.zip › uasa_a_2639734_sm4116.pdf]

Supplementary Material for "Theory for Identification and Inference With Synthetic Controls: A Proximal Causal Inference Framework"

February 25, 2026

This supplement includes extensions of the proximal inference approach to synthetic control, additional simulation studies, and proofs of all theorems.

Contents

A Proof of Theorem 1 **3**

B Proof of Theorem 2 **5**

C Adjustment for measured covariates **6**

D Theoretical guarantees of the methods in Section 3.2 **9**

D.1 Permutation inference approach for the (constrained) GMM 9

D.2 Proof of unbiasedness of Eqs. (8) and (12) and asymptotic normality of $\hat{\xi}$ . . 13

E A nonparametric proximal SC estimator **22**

E.1 Estimation of solution sets 23

E.2 A representer-based estimator 24

E.3 A debiased asymptotically normal estimator for γ 25

F Proof of existence of the confounding bridge function **27**

G Constrained regularized jackknife instrumental variable estimators **28**

H Proof of Theorems 3 and 4 **34**

I Additional discussion on the completeness condition and existence of multiple confounding bridge functions **36**

J Additional simulation studies **39**
J.1 Nonstationary latent factor 39
J.2 Unconstrained SC weights 40
J.3 Weakly dependent errors 41
J.4 Small number of pre-treatment periods 42
J.5 Randomly selected donor and proxy units 44
J.6 Covariate adjustment 45
J.7 Time-varying treatment effect 46
J.8 Nonlinear model 49
J.9 Prediction intervals for θ_{T_0+1}: nonstationary latent factors or unconstrained SC weights 52

K Additional results for the 1990 German reunification analysis **53**

L Additional discussions **54**
L.1 Comparison between our method and that of Freyberger (2018) 54
L.2 Comparison between our method and the interactive fixed effects model based approach 54

A Proof of Theorem 1

Let $A.k$ denote Assumption k for an integer k. We first show that

$$E[Y_t(a)] = E[\mathbb{1}(t > T_0)a\beta_t + \sum_{i\in\mathcal{D}} \alpha_i W_{it}]$$

for any $a = 0, 1$ and any t. Under Assumptions 1-3 we have

$$Y_t(a) \overset{A.1,A.2}{=} \mathbb{1}(t > T_0)a\beta_t + \mu_0^\intercal \lambda_t + \varepsilon_{0t} \tag{S.1}$$

$$\overset{A.3}{=} \mathbb{1}(t > T_0)a\beta_t + \sum_{i\in\mathcal{D}} \alpha_i W_{it} + \varepsilon_{0t} - \sum_{i\in\mathcal{D}} \alpha_i \varepsilon_{it}, \tag{S.2}$$

for $a = 0, 1$ and any t. By Assumption 2, $E[\varepsilon_{it}] = 0$ for any i, t, thus we have

$$E[Y_t(a)] \overset{\text{Eq. } (S.2), A.2}{=} E[\mathbb{1}(t > T_0)a\beta_t + \sum_{i\in\mathcal{D}} \alpha_i W_{it}] \tag{S.3}$$

for $a = 0, 1$ and any t. In Equation (S.3), setting $a = 0$ we have $E[Y_t(0)] = E[\sum_{i\in\mathcal{D}} \alpha_i W_{it}]$ for any t, that is, under Assumptions 1-2, any $\alpha_\mathcal{D}$ that satisfy Assumption 3 also satisfies $E[Y_t(0)] = E[\sum_{i\in\mathcal{D}} \alpha_i W_{it}]$. Therefore, for any $t > T_0$ we have

$$E[Y_t(1) - Y_t(0)] \overset{A.1}{=} E[Y_t - Y_t(0)] = E[Y_t - \sum_{i\in\mathcal{D}} \alpha_i W_{it}].$$

In Equation (S.3) setting $a = 1$ we have

$$E[Y_t(1)] = \beta_t + E[\sum_{i\in\mathcal{D}} \alpha_i W_{it}] = \tau_t + E[\sum_{i\in\mathcal{D}} \alpha_i W_{it}]$$

for any post-treatment period $t > T_0$. Therefore the ATT at time $t > T_0$ is

$$E[Y_t(1) - Y_t(0)] = \tau_t.$$

B Proof of Theorem 2

Under Assumptions 1-3 we have, in the pre-treatment period,

$$E[Y_t \mid \lambda_t] = E[\sum_{i \in \mathcal{D}} \alpha_i W_{it} \mid \lambda_t], \quad \forall t \leq T_0. \tag{S.4}$$

By Assumption 4 we have

$$E[Y_t \mid \lambda_t, Z_t] = E[\sum_{i \in \mathcal{D}} \alpha_i W_{it} \mid \lambda_t, Z_t], \quad \forall t \leq T_0. \tag{S.5}$$

Marginalizing over λ_t on each side of (S.5) with respect to $f(\lambda_t \mid Z_t)$ gives

$$E[Y_t \mid Z_t] = E[\sum_{i \in \mathcal{D}} \alpha_i W_{it} \mid Z_t], \quad \forall t \leq T_0. \tag{S.6}$$

Eq. (S.6) indicates that $\alpha_{\mathcal{D}}$ can in fact be identified upon re-framing the approach in terms of proxies.

C Adjustment for measured covariates

In practice, one may wish to incorporate available covariate data measured across units and over time, either to account for endogeneity or to improve efficiency. Thus we generalize Assumption 2 as follows

Assumption 2″. *For any unit i at time t,*

$$
\begin{aligned}
Y_t(0) &= \mu_0^{\intercal}\lambda_t + C_{0t}^{\intercal}\zeta_0 + \varepsilon_{0t} \\
W_{it}(0) = W_{it}(1) &= \mu_i^{\intercal}\lambda_t + C_{it}^{\intercal}\zeta_i + \varepsilon_{it},
\end{aligned}
\tag{S.7}
$$

where $C_{it} \in \mathcal{R}^p$ is a $p \times 1$ vector of measured covariates, $\zeta_i \in \mathcal{R}^p$ is a $p \times 1$ vector of coefficients, and the error terms satisfy $E[\varepsilon_{it} \mid \lambda_t, C_{it}] = E[\varepsilon_{it}] = 0$ for all $i = 0, \ldots, N$ and t.

In the special case where $\theta_t = \theta$ (fixed constant effect) and $\zeta_i = \zeta$ for all $t = T_0 + 1, \ldots, T$ and $i = 0, 1, \ldots, N$, Assumption 2″ is the same model considered by Xu (2017).

We modify Assumption 4 to further include C_t as potential common causes of treatment assignment and outcome.

Assumption 4″. *We have observed Z_t such that $Z_t \perp\!\!\!\perp (Y_t, W_{\mathcal{D}t}) \mid C_t, \lambda_t$ for any $t \leq T_0$.*

Let $\widetilde{Y}_t = Y_t - C_{0t}^{\intercal}\zeta_0$ and $\widetilde{W}_{it} = W_{it} - C_{it}^{\intercal}\zeta_i$, $i = 1, \ldots, N$. In Corollary 1 below, we state the identification results for τ_t in the presence of measured covariates corresponding to Theorems 1 and 2:

Corollary 1 (Identification in the presence of measured covariates)**.**

(a) Under Assumptions 1, 2″ and 3, we have $E[Y_t(0)] = E[C_{0t}^{\intercal}\zeta_0 + \sum_{i\in\mathcal{D}} \alpha_i \widetilde{W}_{it}]$ for any t,

and the ATT at time t for any $t \geq T_0$ is

$$\tau_t = E[\widetilde{Y}_t - \sum_{i \in \mathcal{D}} \alpha_i \widetilde{W}_{it}]. \tag{S.8}$$

(b) Under Assumptions 1, 2′, 3 and 4″, $\alpha_{\mathcal{D}}$ and ξ_i satisfy the moment condition

$$E[\widetilde{Y}_t - \sum_{i \in \mathcal{D}} \alpha_i \widetilde{W}_{it} \mid Z_t, C_t] = 0, \quad \forall t \leq T_0.$$

Example 1′. *Continuing with Example 1, where the outcomes of control unit(s) not included in the donor pool $W_{\overline{\mathcal{D}}t}$ are selected as supplemental proxy variable Z_t. We have the following estimating function*

$$U_{PI,t}(\alpha_{\mathcal{D}}, \zeta_0, \zeta_{\mathcal{D}}) = g(W_{\overline{\mathcal{D}}t}, C_t) \left(\widetilde{Y}_t - \sum_{i \in \mathcal{D}} \alpha_i \widetilde{W}_{it} \right), \ t = 1, \ldots, T_0, \tag{S.9}$$

where $g(\cdot)$ is a $[|\mathcal{D}| + (|\mathcal{D}| + 1)p]$-dimensional vector of user-specified functions. One can show that $E[U_{PI,t}(\alpha_{\mathcal{D}}, \zeta_0, \zeta_{i,i\in\mathcal{D}})] = 0$ for any $t \leq T_0$.

Furthermore, if the matrix $E[g(W_{\overline{\mathcal{D}}t}, C_t)(W_{\mathcal{D}t}, C_{0t}, C_{\mathcal{D}t})]$ is full row rank, then both the SC weights $\alpha_{\mathcal{D}}$ and the coefficients $(\zeta_{0t}, \zeta_{\mathcal{D}t})$ can be uniquely identified. When the number of measured covariates is large relative to T_0, the resulting estimator may have high variation or even multiple solutions. In such cases, one possible strategy is to assume that measured covariates have the same impact for every unit, i.e. $\zeta_i = \zeta$ for all $i = 1, \ldots, N$ (Xu 2017) and estimate $(\alpha_{\mathcal{D}}, \zeta)$ by using the following unbiased estimating function

$$U_{PI,t}(\alpha_{\mathcal{D}}, \zeta) = g(\widetilde{W}_{jt, j \in [N] \setminus \mathcal{D}}) \left(\widetilde{Y}_t - \sum_{i \in \mathcal{D}} \alpha_i \widetilde{W}_{it} \right), \ t = 1, \ldots, T_0, \tag{S.10}$$

Finally, we extend the above results to the general nonlinear setting, similar to Section 3.3. Below, we modify Assumption 3' to incorporate measured covariates:

Assumption 3'''. *There exist a function $h(W_{\mathcal{D}t}(0), C_t)$ such that the outcome model for $Y_t(0)$ is equivalent to a model for $h(W_{\mathcal{D}t}(0), C_t)$ had the treatment:*

$$E[Y_t(0) \mid \lambda_t, C_t] = E[h(W_{\mathcal{D}t}(0), C_t) \mid \lambda_t, C_t], \quad \forall t \leq T_0. \tag{S.11}$$

We have the following identification results:

Corollary 2 (Nonparametric dentification in the presence of measured covariates)**.**

(a) Under Assumptions 1, 2' and 3''', we have $E[Y_t(0)] = E[h(W_{\mathcal{D}t}, C_t)]$ for any t, and the ATT at time t for any $t \geq T_0$ is

$$\tau_t = E[Y_t - h(W_{\mathcal{D}t}, C_t)]. \tag{S.12}$$

(b) Under Assumptions 1, 2', 3''' and 4'', $h(\cdot)$ satisfy the moment condition

$$E[Y_t - h(W_{\mathcal{D}t}, C_t) \mid Z_t, C_t] = 0, \quad \forall t \leq T_0.$$

Under Model (S.7), it is easy to verify that

$$h(W_{\mathcal{D}t}, C_t) = \sum_{i \in \mathcal{D}} \alpha_i (W_{it} - C_{it}^{\intercal} \xi_i) + C_{0t}^{\intercal} \xi_0.$$

Estimation and inference of the confounding bridge function and treatment effects are similar to before.

D Theoretical guarantees of the methods in Section 3.2

D.1 Permutation inference approach for the (constrained) GMM

In this section, we show that the permutation inference constructed in Section 3.2 with the estimator defined in (9) is approximately valid under some regularity conditions. Specifically, we consider the following estimator of the SC weights vector with the identity weight matrix $\hat{\Omega}$:

$$\hat{\alpha}_{\mathcal{D}} \in \underset{\alpha_{\mathcal{D}} \in \mathcal{W}}{\operatorname{argmin}} \left\{ \frac{1}{T_0+1} \sum_{t=1}^{T_0+1} Z_t \left(Y_t - \sum_{i \in \mathcal{D}} \alpha_i W_{it} \right) \right\}^{\intercal} \left\{ \frac{1}{T_0+1} \sum_{t=1}^{T_0+1} Z_t \left(Y_t - \sum_{i \in \mathcal{D}} \alpha_i W_{it} \right) \right\}, \quad \text{(S.13)}$$

where $\mathcal{W}$ denotes the feasibility set of SC weights. For simplicity of exposition, we write Y_{T_0+1} in place of $Y_{T_0+1} - \theta_{0,T_0+1}$. Here, we choose $\mathcal{W}$ to be a subset of $\{\alpha_{\mathcal{D}} : \sum_{i,i \in \mathcal{D}} |\alpha_i| \leq 1\}$, and assume that the true SC weight $\alpha_{\mathcal{D}}$ belongs to $\mathcal{W}$.

To show the approximate validity of the permutation inference, in the following lemma, we establish a deterministic deviation bound of the constrained estimator $\hat{\alpha}_{\mathcal{D}}$. Denote

$$\widehat{\Sigma}_{ZW} = \frac{1}{T_0+1} \sum_{t=1}^{T_0+1} Z_t W_t^{\intercal} \in \mathcal{R}^{d \times |\mathcal{D}|}, \qquad \widehat{N} = \frac{1}{T_0+1} \sum_{t=1}^{T_0+1} Z_t r_t \in \mathcal{R}^{d},$$

and $\Sigma_{ZW} = E(\widehat{\Sigma}_{ZW})$. Recall that $r_t = \varepsilon_{0t} - \sum_{i \in \mathcal{D}} \alpha_i \varepsilon_{it}$.

Lemma 1. *Suppose that Assumptions 1-5 hold and the true SC weight satisfies $\alpha_{\mathcal{D}} \in \mathcal{W}$. Moreover, for some positive constant c, we assume that $\lambda_{\min}(\Sigma_{ZW}^{\intercal} \Sigma_{ZW}) > c$, where*

$\lambda_{\min}(\Sigma_{ZW}^{\intercal}\Sigma_{ZW})$ *is the smallest eigenvalue of* $\Sigma_{ZW}^{\intercal}\Sigma_{ZW}$. *Then, we have*

$$\|\hat{\alpha}_{\mathcal{D}} - \alpha_{\mathcal{D}}\|_2^2 \leq 4\frac{\|\widehat{\Sigma}_{ZW}^{\intercal}\widehat{N}\|_\infty + \|(\Sigma_{ZW} - \widehat{\Sigma}_{ZW})\Sigma_{ZW}^{\intercal}\|_{\max}}{c}.$$

Proof. Write $\widehat{\Delta} = \hat{\alpha}_{\mathcal{D}} - \alpha_{\mathcal{D}} \in \mathcal{R}^{|\mathcal{D}|}$. Since $\alpha_{\mathcal{D}} \in \mathcal{W}$, we have

$$\left\{\sum_{t=1}^{T_0+1} Z_t\Big(Y_t - \sum_{i,i\in\mathcal{D}} \hat{\alpha}_i W_{it}\Big)\right\}^{\intercal}\left\{\sum_{t=1}^{T_0+1} Z_t\Big(Y_t - \sum_{i,i\in\mathcal{D}} \hat{\alpha}_i W_{it}\Big)\right\} \leq \left\{\sum_{t=1}^{T_0+1} Z_t r_t\right\}^{\intercal}\left\{\sum_{t=1}^{T_0+1} Z_t r_t\right\},$$

which implies that

$$\widehat{\Delta}^{\intercal}\widehat{\Sigma}_{ZW}^{\intercal}\widehat{\Sigma}_{ZW}\widehat{\Delta} \leq 2\widehat{\Delta}^{\intercal}\widehat{\Sigma}_{ZW}^{\intercal}\widehat{N}.$$

Then,

$$\begin{aligned}\widehat{\Delta}^{\intercal}\Sigma_{ZW}^{\intercal}\Sigma_{ZW}\widehat{\Delta} - \widehat{\Delta}(\Sigma_{ZW}^{\intercal}\Sigma_{ZW} - \widehat{\Sigma}_{ZW}^{\intercal}\widehat{\Sigma}_{ZW})\widehat{\Delta} &\leq 2\widehat{\Delta}^{\intercal}\widehat{\Sigma}_{ZW}^{\intercal}\widehat{N} \\ &\leq 4\sup_{\|u\|_1\leq 1}\big(u^{\intercal}\widehat{\Sigma}_{ZW}^{\intercal}\widehat{N}\big) \\ &= 4\|\widehat{\Sigma}_{ZW}^{\intercal}\widehat{N}\|_\infty,\end{aligned}$$

where the second inequality follows from $\|\widehat{\Delta}\|_1 \leq 2$. Moreover, we have $\widehat{\Delta}^{\intercal}\Sigma_{ZW}^{\intercal}\Sigma_{ZW}\widehat{\Delta} \geq c\|\widehat{\Delta}\|_2^2$ and

$$\begin{aligned}&\widehat{\Delta}^{\intercal}(\Sigma_{ZW}^{\intercal}\Sigma_{ZW} - \widehat{\Sigma}_{ZW}^{\intercal}\widehat{\Sigma}_{ZW})\widehat{\Delta} \\ &= \widehat{\Delta}^{\intercal}\{-(\Sigma_{ZW} - \widehat{\Sigma}_{ZW})^{\intercal}(\Sigma_{ZW} - \widehat{\Sigma}_{ZW}) + (\Sigma_{ZW} - \hat{\Sigma}_{ZW})^{\intercal}\Sigma_{ZW} + \Sigma_{ZW}^{\intercal}(\Sigma_{ZW} - \widehat{\Sigma}_{ZW})\}\widehat{\Delta} \\ &\leq 4\sup_{\|u\|_1\leq 1}\big\{u^{\intercal}(\Sigma_{ZW} - \widehat{\Sigma}_{ZW})\big\}\big\{u^{\intercal}\Sigma_{ZW}\big\} \leq 4\|(\Sigma_{ZW} - \widehat{\Sigma}_{ZW})\Sigma_{ZW}^{\intercal}\|_{\max},\end{aligned}$$

where $\|\cdot\|_{\max}$ is the maximum norm of a matrix. Putting the pieces together, we have

$$\|\widehat{\Delta}\|_2^2 \leq 4\frac{\|\widehat{\Sigma}_{ZW}^{\intercal}\widehat{N}\|_\infty + \|(\Sigma_{ZW} - \widehat{\Sigma}_{ZW})\Sigma_{ZW}^{\intercal}\|_{\max}}{c}.$$

□

Lemma 1 provides a deterministic deviation bound for $\hat{\alpha}_{\mathcal{D}}$. In particular, $\hat{\alpha}_{\mathcal{D}}$ is consistent whenever the high-level conditions are satisfied, namely $\|\widehat{\Sigma}_{ZW}^{\intercal}\widehat{N}\|_\infty \to 0$ and $\|(\Sigma_{ZW} - \widehat{\Sigma}_{ZW})\Sigma_{ZW}^{\intercal}\|_{\max} \to 0$, without requiring the data to be covariance-stationary. Based on this lemma, we obtain the following corollary, which shows that our constrained GMM estimator satisfies Assumption 3 of Chernozhukov et al. (2021).

Corollary 3. *Under the same assumptions as in Lemma 1, we additionally assume that the largest eigenvalue of* $(T_0+1)^{-1}\sum_{t=1}^{T_0+1} W_{\mathcal{D}t}W_{\mathcal{D}t}^{\intercal}$ *is bounded in probability and* W_{T_0+1} *is a sub-Gaussian vector. Then, we have*

$$\frac{1}{T_0+1}\sum_{t=1}^{T_0+1}\left\{\sum_{i\in\mathcal{D}}(\hat{\alpha}_i - \alpha_i)W_{it}\right\}^2 = o_{\mathrm{P}}(1) \quad \text{and} \quad \sum_{i\in\mathcal{D}}(\hat{\alpha}_i - \alpha_i)W_{i(T_0+1)} = o_{\mathrm{P}}(1).$$

We remark that when the data is independent across time t, the largest eigenvalue of the covariance matrix of $W_{\mathcal{D}t}$ is bounded, and $W_{\mathcal{D}t}$ is sub-Gaussian, then $(T_0+1)^{-1}\sum_{t=1}^{T_0+1} W_{\mathcal{D}t}W_{\mathcal{D}t}^{\intercal}$ is bounded in probability provided that $|\mathcal{D}|/(T_0+1) = O(1)$. By this corollary, we can apply Theorem 1 of Chernozhukov et al. (2021) provided that $\{r_t\}_{t=1}^{T_0+1}$ are i.i.d. or stationary, strongly mixing with the sum of mixing coefficients bounded by some constant, where $r_t = \varepsilon_{0t} - \sum_{i\in\mathcal{D}}\alpha_i\varepsilon_{it}$, establishing that the permutation test based on the constrained GMM estimator is asymptotically valid as $T_0 \to \infty$.

As in Chernozhukov et al. (2018), if we restrict the feasibility set $\mathcal{W}$ as

$$\mathcal{W} = \mathcal{W}^{\mathrm{SC}} := \left\{ \alpha_{\mathcal{D}} : \sum_{i, i \in \mathcal{D}} \alpha_i = 1, \alpha_i \geq 0 \quad \forall i \in \mathcal{D} \right\},$$

then our estimator can effectively accommodate settings where the outcome and donor units share common but otherwise arbitrary nonstationary components. Specifically, consider the additive fixed effects model in Remark 2, a generalization of the factor model in Assumption 2:

$$Y_t(0) = \mu_0^\intercal \lambda_t + \delta_t + \varepsilon_{0t} \quad \text{and} \quad W_{it} = \mu_i^\intercal \lambda_t + \delta_t + \varepsilon_{it},$$

where $\delta_t \in \mathbb{R}$ is an unrestricted stochastic process that is common across units. Thus, our framework allows for arbitrary time-varying shocks that are shared among all units. This avoids the need to impose a specific structure on the nonstationarity, as is required in some of the SC literature, e.g., Cattaneo et al. (2021) and Masini & Medeiros (2021).

To analyze the asymptotic properties of our estimator under this setup, note that if the true synthetic control weights also lie in $\mathcal{W}^{\mathrm{SC}}$, then (S.13) can be written as

$$\hat{\alpha}_{\mathcal{D}} \in \underset{\alpha_{\mathcal{D}} \in \mathcal{W}}{\operatorname{argmin}} \left\{ \frac{1}{T_0 + 1} \sum_{t=1}^{T_0+1} Z_t \left(\tilde{Y}_t - \sum_{i \in \mathcal{D}} \alpha_i \tilde{W}_{it} \right) \right\}^\intercal \left\{ \frac{1}{T_0 + 1} \sum_{t=1}^{T_0+1} Z_t \left(\tilde{Y}_t - \sum_{i \in \mathcal{D}} \alpha_i \tilde{W}_{it} \right) \right\},$$

where

$$\tilde{Y}_t = \mu_0^\intercal \lambda_t + \varepsilon_{0t}, \quad \text{and} \quad \tilde{W}_{it} = \mu_i^\intercal \lambda_t + \varepsilon_{it}.$$

Hence, Lemma 1 can be applied with $(\tilde{Y}_t, \tilde{W}_{\mathcal{D}t})^\intercal$, thereby validating the proposed method under the common nonstationary components. We also note that the assumption of common nonstationarity across units can be relaxed by following arguments similar to those in

Chernozhukov et al. (2018); see Section 4.2 therein for details.

Suppose the data are independent across t, each row of Z and W is sub-Gaussian, and r_t is mean-zero sub-Gaussian conditional on Z_t. Then, $\|(\Sigma_{ZW} - \widehat{\Sigma}_{ZW})\Sigma_{ZW}^{\intercal}\|_{\max}$ converges to 0 provided that $\|\Sigma_{ZW}\|_1$ is bounded and $n \gtrsim \log(d) + \log(|\mathcal{D}|)$. Similarly, it can be shown that $\|\widehat{\Sigma}_{ZW}^{\intercal}\widehat{N}\|_2$ converges to 0 when $d = o(n)$. A similar conclusion holds when the data exhibit temporal dependence under weak dependence conditions, for example, when the process is β-mixing with exponentially decaying coefficients; for the definition of β-mixing, see Doukhan (1994) for properties and examples of β-mixing conditions.

D.2 Proof of unbiasedness of Eqs. (8) and (12) and asymptotic normality of $\hat{\xi}$

Let $A.k$ denote Assumption k and $T.k$ denote Theorem k for an integer k. We first show that $E[U_t(\alpha)] = 0,\ t \leq T_0$ and $E[\widetilde{U}_t(\theta)] = 0$ at the true value under Assumptions 1-4. Under these assumptions, $\alpha_{\mathcal{D}}$ satisfies $E[Y_t - \sum_{i\in\mathcal{D}} \alpha_i W_{it} \mid Z_{it}] = 0$ for any $t \leq T_0$ by Theorem 2. Therefore, for $t \leq T_0$ we have

$$\begin{aligned} E[U_t(\alpha_{\mathcal{D}})] =&\ E\left[Z_t\left(Y_t - \sum_{i\in\mathcal{D}} \alpha_i W_{it}\right)\right] \\ =&\ E\left[Z_t E[Y_t - \sum_{i\in\mathcal{D}} \alpha_i W_{it} \mid Z_t]\right] \\ \overset{T.2}{=}&\ 0 \end{aligned} \tag{S.14}$$

and

$$E[\widetilde{U}_t(\theta)] = \begin{pmatrix} E[U_t(\alpha_{\mathcal{D}})] \\ 0 \end{pmatrix} \tag{S.15}$$
$$\overset{\text{Eq. }(S.14)}{=} 0.$$

For $t > T_0$, we have

$$E[\widetilde{U}_t(\theta)] = \begin{pmatrix} 0 \\ E[Y_t - \tau_t - \sum_{i\in\mathcal{D}} \alpha_i W_{it}] \end{pmatrix} \tag{S.16}$$
$$\overset{T.1}{=} 0.$$

Now we focus on proving the asymptotic normality of the proposed estimator. Following the approach in Chapters 7 and 11 of Pötscher & Prucha (1997), we establish consistency and asymptotic normality under nonstationary data. Let $O_t = \{Y_t, Z_t^\intercal, W_{\mathcal{D}t}^\intercal\} \in \mathcal{R}^{1+d+|\mathcal{D}|}$ denote the observable vector of random variables, and define the parameter vector $\xi = (\alpha_{\mathcal{D}}^\intercal, \tau)^\intercal \in \mathcal{W}^{1+|\mathcal{D}|}$ and the true parameter ξ_0 with parameter space $\Theta \subseteq \mathcal{R}^{1+|\mathcal{D}|}$ and sample space $\mathcal{O} \subseteq \mathcal{R}^{1+d+|\mathcal{D}|}$. As shown previously, the population moment condition satisfies $\mathbb{E}\tilde{U}_t(\xi) = 0$, where the moment function is defined in Eq. (12). Note that $\tilde{U}_t = \tilde{U}_t(O_t; \xi)$ is a mapping from $\mathcal{O} \times \Theta$ to $\mathcal{R}^{1+d}$.

To introduce regularity assumptions, we first provide the definition of α-mixing (Doukhan 1994).

Definition 1. *For any stochastic process $\{\eta_t\}_{t=-\infty}^{\infty}$, the lag-$j$ α-mixing dependence coefficient is defined as*

$$\alpha(j) = \sup_{k\in\mathbb{Z}} \sup |P(A \cap B) - P(A)P(B)|,$$

where the supremum is taken over all events $A \in \sigma(\{\eta_t\}_{t=-\infty}^{k})$ *and* $B \in \sigma(\{\eta_t\}_{t=k+j}^{\infty})$*, and* $\sigma(\cdot)$ *is the sigma field generated by the process.*

The assumption that the data is α-mixing has been widely used in the econometrics literature, but it can be restrictive for dynamic models as discussed in Chapter 6 of Pötscher & Prucha (1997). Thus, the following approximation concepts are introduced.

Definition 2. *Let* $\{v_t\}_{t=-\infty}^{\infty}$ *and* $\{\eta_t\}_{t=-\infty}^{\infty}$ *be stochastic processes defined on the same probability space. Then, the process* $\{v_t\}_{t=-\infty}^{\infty}$ *is called* L_0*-approximable by the basis process* $\{\eta_t\}_{t=-\infty}^{\infty}$ *if there exist measurable functions* h_t^m *such that for every* $\delta > 0$*, we have*

$$\limsup_{T\to\infty} \frac{1}{T}\sum_{t=1}^{T} P(|v_t - h_t^m(\eta_{t+m}, \ldots, \eta_{t-m})| > \delta) \to 0 \quad \text{as } m \to \infty.$$

The concept of L_0-approximability coincides with the notion of stochastic stability introduced by Bierens (1981). As discussed in Chapter 17 of Davidson (1994), it provides a convenient framework for analyzing transformations of dependent variables. Examples of random processes satisfying this property are given in Andrews (1988) and Davidson (1994).

Now, we are ready to provide regularity conditions.

Assumption D.1. $\{O_t\}_{t=-\infty}^{\infty}$ *is* L_0*-approximable by some* α*-mixing basis process, say* $\{\eta_t\}_{t=-\infty}^{\infty}$.

We remark that this assumption encompasses the setting where the data is non-stationary, e.g., non-stationary unobserved common factors λ_t.

Assumption D.2. Θ *is a compact set.*

Assumption D.2 follows standard regularity conditions commonly imposed in the GMM literature (see, for example, Hall 2005).

Assumption D.3. *For some* $\gamma > 0$*, we have*

$$\sup_T \frac{1}{T}\sum_{t=1}^{T} E\Big\{ \max_{1\le j\le d}\Big(|Z_{tj}r_t|^{1+\gamma} + \|Z_{tj}W_{\mathcal{D}t}\|_2^{1+\gamma}\Big) + \|W_{\mathcal{D}t}\|_2^{1+\gamma} + |r_t|^{1+\gamma}\Big\} < \infty.$$

A straightforward sufficient condition for Assumption D.3 is that all variables and their products have uniformly bounded $(1+\gamma)$-moments over t.

Assumption D.4 (Properties of the Weighting Matrix)**.** *The user-specified weight matrix* $\widehat{\Omega}$ *is a positive semi-definite matrix, possibly depends on data, and converges in probability to the positive definite matrix* Ω*.*

We note that this assumption is trivially satisfied when the weight matrix is fixed, for example, as the identity matrix.

Assumption D.5. $\xi_0 = (\alpha_{\mathcal{D}}^{\intercal}, \bar{\tau})^{\intercal}$ *is the unique solution solving*

$$\lim_{T\to\infty} \frac{1}{\sqrt{T}} \sum_{t=1}^{T} E\{\tilde{U}_t(\xi)\} = 0.$$

A sufficient condition for Assumption D.5 is the full-rank condition in Assumption 5, although Assumption D.5 encompasses more general settings.

Under Assumptions 1–5 and Assumptions D.1–D.5, $\widehat{\xi}$ is consistent by Theorem 7.1 of Pötscher & Prucha (1997).

To establish the asymptotic normality, we need the following additional assumptions.

Assumption D.6. *The sequence* $\bar{H}_T^O : T \in \mathbb{N}$ *is tight on* O*, where* $\bar{H}_T^O = T^{-1}\sum_{t=1}^{T} H_t^O$ *and* H_t^O *is the distribution of* O_t*.*

A sufficient condition for Assumption D.6 is that O_t has finite moments. We refer readers to Section 5.1 of Pötscher & Prucha (1997) for further details.

Assumption D.7. *Assume that*

$$\liminf_{n\to\infty} \lambda_{\min}\left(E\left\{\frac{1}{T}\sum_{t=1}^{T}\frac{\partial}{\partial\xi}\tilde{U}_t(O_t;\xi_0)\right\}^{\intercal} E\left\{\frac{1}{T}\sum_{t=1}^{T}\frac{\partial}{\partial\xi}\tilde{U}_t(O_t;\xi_0)\right\}\right) > 0.$$

Moreover, there exists a positive definite matrix S such that

$$\frac{1}{T}E\left[\left\{\sum_{t=1}^{T}\frac{\partial}{\partial\xi}\tilde{U}_t(O_t;\xi_0)\right\}^{\intercal}\left\{\sum_{t=1}^{T}\frac{\partial}{\partial\xi}\tilde{U}_t(O_t;\xi_0)\right\}\right] \to S \quad \textit{as} \ \ T\to\infty.$$

This assumption necessitates the existence of some covariance matrices.

Assumption D.8. *Given the basis process $\{\eta_t\}_{t=-\infty}^{\infty}$, we assume that*

$$\sup_t \left\{\|Z_t r_t - E(Z_t r_t|\eta_{t+m},\ldots,\eta_{t-m})\|_2 + |r_t - E(r_t|\eta_{t+m},\ldots,\eta_{t-m})|\right\} = O(m^{-\lambda})$$

for some $\lambda > 1$. Furthermore, for some $\gamma > 0$, we have

$$\sup_T \sup_{1\le t\le T} E\left\{\max_{1\le j\le d}\left(|Z_{tj}r_t|^{2+\gamma} + \|Z_{tj}W_{\mathcal{D}t}\|_2^{2+\gamma}\right) + \|W_{\mathcal{D}t}\|_2^{2+\gamma} + |r_t|^{2+\gamma}\right\} < \infty.$$

and $\{\eta_t\}_{t=-\infty}^{\infty}$ is α-mixing with mixing coefficients of size $-2(\gamma+2)/\gamma$.

Assumption D.8 imposes a stronger condition than Assumption D.3. The α-mixing requirement can be relaxed when the random variables possess uniformly bounded higher-order moments.

Under Assumptions 1–5 and Assumptions D.1–D.8, Theorem 11.5 of Pötscher & Prucha (1997) implies that

$$\sqrt{T}(\widehat{\xi} - \xi_0) = C_T^{-1} D_T \zeta_T + o_{\mathrm{P}}(1),$$

where $\zeta_T \xrightarrow{d} N(0, S)$, and

$$C_T = E\left\{\frac{1}{T}\sum_{t=1}^{T}\frac{\partial}{\partial \xi}\tilde{U}_t(O_t;\xi_0)\right\}^{\intercal}\tilde{\Omega}E\left\{\frac{1}{T}\sum_{t=1}^{T}\frac{\partial}{\partial \xi}\tilde{U}_t(O_t;\xi_0)\right\},$$

and

$$D_T = E\left\{\frac{1}{T}\sum_{t=1}^{T}\frac{\partial}{\partial \xi_0}\tilde{U}_t(O_t;\xi)\right\}^{\intercal}\tilde{\Omega}.$$

In particular, if $E\{T^{-1}\sum_{t=1}^{T}\partial\tilde{U}_t(O_t;\xi_0)/\partial\xi\} \to \Sigma_0$ for some matrix Σ_0, then it follows that

$$\sqrt{T}(\widehat{\xi} - \xi_0) \xrightarrow{d} N(0, (\Sigma_0^{\intercal}\Omega\Sigma_0)^{-1}\Sigma_0^{\intercal}\Omega S\Omega\Sigma_0(\Sigma_0^{\intercal}\Omega\Sigma_0)^{-1}).$$

A consistent estimator of

$$\Sigma = (\Sigma_0^{\intercal}\Omega\Sigma_0)^{-1}\Sigma_0^{\intercal}\Omega S\Omega\Sigma_0(\Sigma_0^{\intercal}\Omega\Sigma_0)^{-1},$$

denoted by $\widehat{\Sigma}$, can be obtained by replacing Ω, S, and Σ_0 with their respective estimators $\tilde{\Omega}$, $\widehat{S}$, and $\widehat{\Sigma}_0$. Here, $\widehat{S}$ corresponds to the heteroskedasticity-consistent (HC) estimator of S when U_t is serially uncorrelated (White 1980), and to the heteroskedasticity- and autocorrelation-consistent (HAC) estimator of S when U_t exhibits serial correlation (Newey & West 1986). The matrix $\widehat{\Sigma}_0$ is the empirical counterpart of Σ_0 with the estimate of θ substituted in. Both estimation and inference can be conveniently implemented using standard R packages such as `gmm`.

If we choose $\widetilde{\Omega}$ such that $\widetilde{\Omega} \to S^{-1}$, then we have

$$\sqrt{T}(\widehat{\xi} - \xi_0) \xrightarrow{d} N(0, (\Sigma_0^{\intercal}S^{-1}\Sigma_0)^{-1}),$$

which is asymptotically efficient (Newey & McFadden 1994).

Finally, we consider a general user-specified parametric model for time-varying treatment effects $\tau_t = \tau(t/T;\gamma)$, indexed by a parameter γ with dimension d_γ. We consider estimating the parameters $\xi = (\alpha^\intercal, \gamma^\intercal)^\intercal$ by GMM with the estimating function

$$\dot{U}_t(\xi) = \begin{pmatrix} \mathbb{1}(t \le T_0) g_1(Z_t)(Y_t - \sum_{i\in\mathcal{D}} \alpha_i W_{it}) \\ \mathbb{1}(t > T_0) g_2(t)[Y_t - \tau(t/T;\gamma) - \sum_{i\in\mathcal{D}} \alpha_i W_{it}] \end{pmatrix}$$
$$= \begin{pmatrix} \mathbb{1}(t \le T_0) g_1(Z_t) \\ \mathbb{1}(t > T_0) g_2(t) \end{pmatrix} [Y_t - \tau(t/T;\gamma) - \sum_{i\in\mathcal{D}} \alpha_i W_{it}], \quad \text{(S.17)}$$

where g_1 and g_2 are real functions with dimensions d and d_γ, respectively.

By a similar argument as above, it can be shown that $E[\dot{U}_t(\xi)] = 0$. Furthermore, let $\dot{\mathcal{D}}_t = \{W_{\mathcal{D}t}{}^\intercal, \mathbb{1}(t > T_0)[\partial\tau(t/T;\gamma)/\partial\gamma]^\intercal\}^\intercal \in \mathcal{R}^{|\mathcal{D}|+d_\gamma}$, $\dot{\mathcal{V}}_t = \{\mathbb{1}(t \le T_0) g_1(Z_t)^\intercal, \mathbb{1}(t > T_0) g_2(t)\}^\intercal \in \mathcal{R}^{d+d_\gamma}$, and $\widetilde{\Omega}'$ is a $(d+d_\gamma)\times(d+d_\gamma)$ positive definite weighting matrix whose upper-left submatrix equals $\widehat{\Omega}$. Suppose that Assumptions 1-6 with $\tau_t = \tau(t/T;\gamma)$ and Assumptions D.1–D.8 hold, and the condition that the matrix $E[g_2(t)\{\partial\tau(t/T;\gamma)/\gamma\}^T]$ is full row rank for any $t > T_0$ and γ in a neighborhood of the true value. Then, provided that $\widehat{\Omega}' \to \dot{\Omega}$, the resulting estimator $\widehat{\xi}$ satisfies

$$\sqrt{T}(\widehat{\xi} - \xi) \xrightarrow{d} N(0, (\dot{\Sigma}_0^\intercal \dot{\Omega} \dot{\Sigma}_0)^{-1} \dot{\Sigma}_0^\intercal \dot{\Omega} \dot{S} \dot{\Omega} \dot{\Sigma}_0 (\dot{\Sigma}_0^\intercal \dot{\Omega} \dot{\Sigma}_0)^{-1}),$$

where

$$\dot{\Sigma}_0 = \lim_{T\to\infty} E\left\{ \frac{1}{T} \sum_{t=1}^{T} \frac{\partial}{\partial \xi} \dot{U}_t(O_t;\xi_0) \right\},$$

and

$$\dot{S} = \lim_{T\to\infty} \frac{1}{T} E\left[\left\{ \sum_{t=1}^{T} \frac{\partial}{\partial \xi} \dot{U}_t(O_t;\xi_0) \right\}^{\intercal} \left\{ \sum_{t=1}^{T} \frac{\partial}{\partial \xi} \dot{U}_t(O_t;\xi_0) \right\}\right].$$

We focus on the unconstrained GMM framework for hypothesis testing based on asymptotic distributions (Section 3.2.3) to emphasize the core identification and simplify the exposition, while constrained estimators are employed for the permutation-based inference procedures that admit theoretical guarantees. This choice reflects our intention to present the main identification argument as clearly as possible, without the additional technical complications introduced by feasibility constraints. While the constrained GMM estimator with the simplex restriction can, in principle, be analyzed using the general results of Andrews (1999), the resulting asymptotic distribution depends on whether constraints are binding, often becoming non-standard and invalidating conventional inference methods such as the bootstrap. Recent studies, including Ketz (2018) and Li (2024), develop valid inference procedures for constrained estimators, with Li (2024) providing explicit results for constrained GMM estimators that could, in principle, be applied to our setting to construct hypothesis tests. Nevertheless, one of the main motivations for imposing the simplex constraint is to mitigate extrapolation, which frequently arises when T_0 and $|\mathcal{D}|$ are of comparable magnitude. Under this regime, however, most existing theoretical results in the aforementioned literature no longer apply. Therefore, incorporating these technical results for constrained estimators would substantially complicate the exposition without fundamentally addressing the limitations inherent in the unconstrained GMM. We also note that, to avoid potential misspecification of the simplex feasibility set, recent work has considered broader sets such as $\mathcal{W} = \{\alpha_{\mathcal{D}} \in \mathbb{R}_+^{|\mathcal{D}|} : \sum_{i\in\mathcal{D}} |\alpha_i| \le 1\}$ (Chernozhukov et al. 2018, 2021) or $\mathcal{W} = \mathbb{R}^{|\mathcal{D}|}$ (Hsiao et al. 2012, Doudchenko & Imbens 2016), albeit at the cost of interpretability. In line with these

studies, our theoretical development for hypothesis testing based on asymptotic distributions focuses on the unconstrained GMM.

E A nonparametric proximal SC estimator

In Theorem 3, we stated that ETT can be identified even when there are multiple confounding bridge functions h. In the presence of multiple solutions, one strategy to proceed with inference is to select an optimal estimator that satisfies pre-specified criteria. In this section we present a nonparametric series estimator based on recent a work of nonparametric treatment effect estimation in proximal causal inference (Zhang et al. 2023), omitting technical proofs and regularity conditions. Although we introduce the estimator in the most general nonparametric setting, the method can incorporate parametric confounding bridge functions, as presented in Section 3.3, with simple modification.

Recall that estimation of ETT requires estimating confounding bridge functions as the solutions to

$$E[Y_t - h(W_{\mathcal{D}t}) \mid Z_t] = 0, \quad \forall t \leq T_0. \tag{S.18}$$

Therefore we first consider estimating the solution set of Equation (S.18). Let $\mathcal{H}$ be a prespeficied set of smooth functions. Define the solution sets of Equation (S.18) as

$$\mathcal{H}_0 = \{h \in \mathcal{H} : E[h(W_{\mathcal{D}t}) \mid Z_t] = E[Y_t \mid Z_t], \quad \forall t \leq T_0\}. \tag{S.19}$$

Under the assumptions from Theorem 3.3, the ATT can be identified as $\tau_t = E[Y_t - h(W_{\mathcal{D}t})]$ for any $t > T_0$. Under these assumptions, to estimate τ_t, we first construct a consistent estimator $\widehat{\mathcal{H}}_0$ for the set $\mathcal{H}_0$. Next, we select a specific $\hat{h}_0 \in \widehat{\mathcal{H}}_0$ so that it is a consistent estimator for a fixed $h_0 \in \mathcal{H}_0$.

E.1 Estimation of solution sets

Define a criterion function

$$C(h) = E[E[Y_t - h(W_{\mathcal{D}t}) \mid Z_t]^2], \quad t \leq T_0.$$

Note that $\mathcal{H}_0 = \{h \in \mathcal{H} : C(h) = 0\}$. We consider a two-stage approach for estimation. We let $\mathcal{H}_n$ be sieve for $\mathcal{H}$, that is, for a known seuence of approximating functions $\{\phi_m(w)\}_{m=1}^{\infty}$, let $\mathcal{H}_n = \{h \in \mathcal{H} : h(w) = \sum_{m=1}^{m_n} \tau_m \phi_m(w)\}$ for an unknown constant τ_m and a prespecified constant m_n. $\mathcal{H}_n$ may also be defined as a parametric model. To construct a sample analogue C_n of C, we let $\{\psi_k(z)\}_{k=1}^{\infty}$ be a known sequence of approximating functions. Denote

$$\psi(z) = \{\psi_1(z), \ldots, \psi_{k_n}(z)\}^\intercal$$

and let $\Psi = \{\psi(Z_1), \ldots, \psi(Z_{T_0})\}^\intercal$. We estimate $E[Y_t \mid Z_t = z_t]$ and $E[h(W_{\mathcal{D}t}) \mid Z_t = z_t]$ respectively with

$$\hat{E}[Y_t \mid Z_t = z_t] = \psi(z_t)(\Psi^\intercal \Psi)^{-1} \sum_{t'=1}^{T_0} \psi(Z_{t'}) Y_{t'}$$

and

$$\hat{E}[h(W_{\mathcal{D}t}) \mid Z_t = z_t] = \psi(z_t)(\Psi^\intercal \Psi)^{-1} \sum_{t'=1}^{T_0} \psi(Z_{t'}) h(W_{\mathcal{D}t'})$$

We then estimate $C(h)$ by

$$C_n(h) = \frac{1}{n} \sum_{t=1}^{T_0} \hat{e}^2(Z_t, h)$$

where $\hat{e}(Z_t, h) = \hat{E}[Y_t \mid Z_t] - \hat{E}[h(W_{\mathcal{D}t}) \mid Z_t]$.

E.2 A representer-based estimator

After obtaining $\widehat{\mathcal{H}_0}$, we select a specific estimator from $\widehat{\mathcal{H}_0}$ that converges to a unique element in $\mathcal{H}_0$. We let $M : \mathcal{H} \to \mathbb{R}$ be a population criterion function that attains a unique minimum h_0 on $\mathcal{H}_0$ and let $M_n(h)$ be its sample analogue. We then select $\hat{h}_0 \in \operatorname*{argmin}_{h\in\widehat{\mathcal{H}_0}} M_n(h)$. A possible choice for M and M_n are the squared norm $M(h) = E[h(W_{\mathcal{D}t})^2]$ and its sample analog $M_n(h) = \frac{1}{T_0}\sum_{t=1}^{T_0} h(W_{\mathcal{D}t})$. For a unique solution $\hat{h}_0$ to be attained, we make the following assumptions:

Assumption E.1. *The function set $\mathcal{H}$ is convex; the functional $M : \mathcal{H} \to \mathbb{R}$ is strictly convex and attains a unique minimum at h_0 on $\mathcal{H}_0$; its sample analogue $M_n : \mathcal{H} \to \mathbb{R}$ is continuous and $\sup_{h\in\mathcal{H}}|M_n(h) - M(h)| = o_p(1)$.*

With the estimated $\hat{h}_0$, a simple estimator for $\hat{\tau}_t$ is $Y_t - \hat{h}_0(W_{\mathcal{D}t})$. Inference may be attained by the conformal inference approach in Section 3.2.1. It is possible to use a parametric model for the treatment effect as in Section 3.2.3, and an asymptotically normal estimator of the treatment effect may be achieved by de-biasing the resulting estimators.

Similar to Section 3.2.3, we consider a parametric model for the treatment effect $\tau(t/T;\gamma)$. With a fixed function $\hat{h}_0$, the parameter γ may be estimated by solving the equation

$$\frac{1}{T - T_0}\sum_{t=T_0+1}^{T} [Y_t - \tau(t/T;\gamma) - \hat{h}_0(W_{\mathcal{D}t})] = 0.$$

We make the following representer assumption:

Assumption E.2. *There exists a function $g_0 \in \mathcal{H}$ such that $\langle g_0, h\rangle_w := E[E\{g_0(W_{\mathcal{D}t}) \mid Z_t\}E\{h(W_{\mathcal{D}t}) \mid Z_t\}]$, $t > T_0$, for all $h \in \bar{\mathcal{H}}$, where $\bar{\mathcal{H}}$ denotes the closure of $\mathcal{H}$.*

Without proof, we stated that assuming stationary, independent errors and under certain

regularity conditions, the resulting estimator γ satisfies

$$\sqrt{T_1}(\hat{\gamma}-\gamma_0) = \left(\frac{1}{\sqrt{T_1}}\sum_{t=T_0+1}^{T}\frac{\partial}{\partial\gamma}\tau(t/T;\gamma_0)\right)^{-1}\Bigg\{\frac{1}{\sqrt{T_1}}\sum_{t=T_0+1}^{T}\Big[Y_t - E[Y_t] - h_0(W_{\mathcal{D}t}) + E[Y_t(0)]$$
$$- E\{g_0(W_{\mathcal{D}t}) \mid Z_t\} \times (Y_t(0) - h_0(W_{\mathcal{D}t}))\Big] + \sqrt{T_1}r_n(\hat{h}_0)\Bigg\} + o_p(1)$$

where

$$r_n(\hat{h}_0) = \frac{1}{T_1}\sum_{t=T_0+1}^{T}\hat{E}\{\Pi_n g_0(W_{\mathcal{D}t}) \mid Z_t\}e'(Z_t,\hat{h}_0)$$

and

$$e'(Z_t,h) = E[Y_t(0) \mid Z_t] - E[h(W_{\mathcal{D}t}) \mid Z_t].$$

E.3 A debiased asymptotically normal estimator for γ

An asymptotic estimator for γ and thus $\tau(t/T;\gamma)$ requires estimating the term $r_n(\hat{h}_0)$. We define a new criterion function

$$R(h) = E[E\{h(W_{\mathcal{D}t}) \mid Z_t\}^2] - 2E[h(W_{\mathcal{D}t})], \quad h \in \mathcal{H}$$

and its sample analog

$$R_n(h) = \frac{1}{T_1}\sum_{t=T_0+1}^{T}\hat{E}[h(W_{\mathcal{D}t}) \mid Z_t]^2 - \frac{2}{T_1}\sum_{t=T_0+1}^{T}h(W_{\mathcal{D}t}), \quad h \in \mathcal{H}.$$

We an then estimate the term $\Pi_n g_0$ by

$$\hat{g} \in \underset{h\in\mathcal{H}_n}{\operatorname{argmin}} R_n(h).$$

We can then construct an estimator for $r_n(\hat{h}_0)$ as

$$\hat{r}_n(\hat{h}_0) = \frac{1}{T_1} \sum_{t=T_0+1}^{T} \hat{E}\{\hat{g}(W_{\mathcal{D}t}) \mid Z_t\} \hat{e}'(Z_t, \hat{h}_0).$$

Here $\hat{e}'(Z_t, h) = \hat{E}[Y_t(0) \mid Z_t] - \hat{E}[h(W_{\mathcal{D}t}) \mid Z_t]$, where $\hat{E}[Y_t(0) \mid Z_t]$ and $E[h(W_{\mathcal{D}t}) \mid Z_t]$ are consistent estimators for $E[Y_t(0) \mid Z_t]$ and $E[h(W_{\mathcal{D}t}) \mid Z_t]$ respectively.

Let $\{\psi'_k(z)\}_{k=1}^{\infty}$ be a known sequence of approximating functions. Denote

$$\psi'(z) = \{\psi'_1(z), \ldots, \psi'_{k_n}(z)\}^{\mathsf{T}}$$

and let $\Psi' = \{\psi'(Z_{T_0+1}), \ldots, \psi'(Z_T)\}^{\mathsf{T}}$. We may set

$$\hat{E}[\hat{g}(W_{\mathcal{D}t}) \mid Z_t = z_t] = \psi'(z_t)(\Psi'^{\mathsf{T}}\Psi')^{-1} \sum_{t'=T_0+1}^{T} \psi'(Z_{t'})\hat{g}(W_{\mathcal{D}t'}),$$

$$\hat{E}[Y_t(0) \mid Z_t = z_t] = \psi'(z_t)(\Psi'^{\mathsf{T}}\Psi')^{-1} \sum_{t'=1}^{T_0} \psi(Z_{t'})[Y_{t'} - \tau(t'/T, \hat{\gamma})]$$

and

$$\hat{E}[h(W_{\mathcal{D}t}) \mid Z_t = z_t] = \psi'(z_t)(\Psi'^{\mathsf{T}}\Psi')^{-1} \sum_{t'=T_0+1}^{T} \psi(Z_{t'})h(W_{\mathcal{D}t'})$$

An asymptotically normal debiased estimator for γ is

$$\hat{\gamma}_{db} = \hat{\gamma} - \left(\frac{1}{\sqrt{T}_1} \sum_{t=T_0+1}^{T} \frac{\partial}{\partial \gamma} \tau(t/T; \hat{\gamma}) \right)^{-1} \hat{r}_n(\hat{h}_0).$$

An asymptotically linear estimator for $\tau(\rho, \gamma)$ ($\rho \in (T_0/T, 1]$) is then $\tau(\rho, \hat{\gamma}_{db})$, of which the inference can be obtained via standard the delta-method.

F Proof of existence of the confounding bridge function

To simplify notation, for each $t \geq 1$, let $W = W_{\mathcal{D}t}(0)$, $Y = Y_t(0)$, and $\lambda = \lambda_t$. Then Eq. (13) holds if

$$f(y \mid \lambda) = \int h(w) f(w \mid \lambda) dw, \tag{S.20}$$

which is a Fredholm integral equation of the first kind. Conditions for existence of a solution has been considered in Miao et al. (2018) and Cui et al. (2024). Let $L^2[F(s)]$ denote the space of all square-integrable functions of s with respective to a cumulative distribution function $F(s)$, which is a Hilbert space with inner product $< g, h >= \int_{-\infty}^{\infty} g(s)h(s)dF(s)$. Let K denote the conditional expectation operator $L^2[F(w)] \to L^2[F(\lambda)]$, with $Kh = E[h(w) \mid \lambda]$ for $h \in L^2\{F(w)\}$, and let $(\tau_n, \phi_n, \psi_n)_{n=1}^{\infty}$ denote a singular value decomposition of K. We assume the following regularity conditions:

Condition F.1: $\int\int f(w \mid \lambda) f(\lambda \mid w) dw d\lambda < \infty$;

Condition F.2: $\int f^2(y \mid \lambda) f(\lambda) d\lambda < \infty$;

Condition F.3: $\sum_{n=1}^{\infty} | < f(y \mid \lambda), \psi_n > |^2 < \infty$.

Condition F.4: Let q be an square-integrable function. If $\int q(\lambda) f(\lambda \mid w) d\lambda = 0$ almost surely, then $q(\lambda) = 0$ almostly.

Then by Picard's theorem (Kress 1989), there exist a solution to Eq. (S.20), which then satisfies Eq. (13).

G Constrained regularized jackknife instrumental variable estimators

In Section D.1, we discuss the consistency of the constrained GMM estimator. We remark that when $d \asymp n$, this estimator may not be consistent. In detail, when $d \asymp n$, the term $\|\widehat{\Sigma}_{ZW}^{\intercal} \widehat{N}\|_{\max}$ may fail to converge to 0, thus the bound in Lemma 1 may not converge to 0. To see this, for $i \in \mathcal{D}$, the i-th component of $\widehat{\Sigma}_{ZW}^{\intercal} \widehat{N}$ can be written as

$$\frac{1}{(T_0+1)^2} \sum_{1 \le s,t \le T_0+1} W_{it}(Z_t^{\intercal} Z_s) r_s,$$

with its expectation

$$\frac{1}{(T_0+1)^2} \sum_{t=1}^{T_0+1} E(W_{it} \|Z_t\|_2^2 r_t).$$

Since W_{ti} and r_t are correlated, we generally have $\mathbb{E}(W_{ti} r_t \mid Z_t) \neq 0$. Consequently, without additional assumptions, the above expectation is bounded as

$$\frac{1}{(T_0+1)^2} \sum_{t=1}^{T_0+1} E(W_{it} \|Z_t\|_2^2 r_t) \lesssim \frac{1}{(T_0+1)^2} \sum_{t=1}^{T_0+1} E(\|Z_t\|_2^2) \lesssim \frac{d}{T_0}.$$

Thus, we require $d = o(T_0)$. Since we assume that Σ_{ZW} is full column rank, this explicitly requires $d \geq |\mathcal{D}|$. In summary, the constrained GMM estimator with the identity weight matrix can accommodate dependent data structures and does not rely on covariance stationarity. However, without stronger assumptions, the associated permutation test is valid only when T_0 is sufficiently large relative to both the number of donors and the number of proxies.

Since $|\mathcal{D}|$ and T_0 are frequently of comparable order in SC applications, this drawback can

be critical in practice. To address this, we can alternatively consider the ridge-regularized jackknife instrumental variables estimator (JIVE) proposed in Hansen & Kozbur (2014). In detail, denoting λ to be the regularization parameter, define

$$P = P_\lambda =: \frac{1}{T_0+1} Z\big(\widehat{\Sigma}_{ZZ} + \lambda I_d\big)^{-1} Z^\intercal,$$

where $\widehat{\Sigma}_{ZZ} = (T_0+1)^{-1}\sum_{t=1}^{T_0+1} Z_t Z_t^\intercal$ and I_d is the $d \times d$ identity matrix. Morevoer, denoting $\mathrm{diag}(P)$ to be the diagonal matrix whose t-th diagonal is P_{tt}, define $Q = \{P - \mathrm{diag}(P)\}$. Then, the estimator is defined as

$$\tilde{\alpha}_{\mathcal{D}} \in \operatorname*{argmin}_{\alpha_{\mathcal{D}} \in \mathcal{W}} \frac{1}{T_0+1}\big(Y - W\alpha_{\mathcal{D}}\big)^\intercal Q \big(Y - W\alpha_{\mathcal{D}}\big),$$

where $Y = (Y_1, \ldots, Y_{T_0+1})^\intercal \in \mathcal{R}^{T_0+1}$ and $W = (W_{\mathcal{D}1}^\intercal, \ldots, W_{\mathcal{D}(T_0+1)}^\intercal)^\intercal \in \mathcal{R}^{(T_0+1)\times|\mathcal{D}|}$. We remark that when $\mathcal{W} = \mathbb{R}^{|\mathcal{D}|}$ and Q is replaced by $\{I - \mathrm{diag}(P)\}^{-1}Q$, the minimizer corresponds to the ridge-regularized JIVE estimator. For simplicity, we work with the unadjusted form, but the subsequent results remain valid under the adjusted form by arguments analogous to those in Hansen & Kozbur (2014).

In the following, we establish the consistency of $\tilde{\alpha}_{\mathcal{D}}$ under regularity conditions that can hold even when $|\mathcal{D}|, d > T_0$. Suppose that $\mathcal{W}$ is a subset of $\{\alpha_{\mathcal{D}} : \sum_{i\in\mathcal{D}} |\alpha_i| \le 1\}$. We consider a similar setting as in Hansen & Kozbur (2014).

Assumption G.1. *There exists a constant matrix $\Gamma \in \mathcal{R}^{|\mathcal{D}|\times d}$ and a random vector $U_{it,i\in\mathcal{D}} \in \mathcal{R}^{|\mathcal{D}|}$ such that $W_{\mathcal{D}t} = \Gamma Z_t + U_t$ with $E(U_t|Z_t) = 0$. Moreover, the smallest eigenvalue of the matrix J defined as*

$$J = \frac{1}{T_0+1}\sum_{t\neq s} \Gamma Z_t P_{st} Z_s^\intercal \Gamma^\intercal$$

is positive with high probability.

For a concrete example where J has a strictly positive minimum eigenvalue, see Example 1 in Hansen & Kozbur (2014).

Lemma 2. *Suppose that Assumptions 1-5 and G.1 hold and the true SC weight satisfies* $\alpha_{\mathcal{D}} \in \mathcal{W}$. *Then, we have*

$$\|\tilde{\alpha}_{\mathcal{D}} - \alpha_{\mathcal{D}}\|_2^2 \leq \frac{4}{\lambda_{\min}(J)} \Bigg\{ \Bigg\| \frac{1}{T_0+1} \sum_{t \neq s} W_{\mathcal{D}t} P_{st} r_s \Bigg\|_\infty + 2 \Bigg\| \frac{1}{T_0+1} \sum_{t \neq s} U_t P_{st} Z_s^\intercal \Gamma^\intercal \Bigg\|_{\max} + \Bigg\| \frac{1}{T_0+1} \sum_{t \neq s} U_t P_{st} U_s \Bigg\|_{\max} \Bigg\}.$$

Proof. Write $\widetilde{\Delta} = \tilde{\alpha}_{\mathcal{D}} - \alpha_{\mathcal{D}}$. By employing a similar argument as in the proof of Lemma 1, we have the following basic inequality,

$$\frac{1}{T_0+1} \widetilde{\Delta}^\intercal W^\intercal Q W \widetilde{\Delta} \leq \frac{2}{T_0+1} \widetilde{\Delta}^\intercal W^\intercal Q r,$$

where $r = (r_1, \ldots, r_{T_0+1})^\intercal \in \mathcal{R}^{T_0+1}$. Then,

$$\begin{aligned} \widetilde{\Delta}^\intercal J \widetilde{\Delta} - \widetilde{\Delta}^\intercal \left(J - \frac{1}{T_0+1} W^\intercal Q W \right) \widetilde{\Delta} &\leq \frac{2}{T_0+1} \widetilde{\Delta}^\intercal W^\intercal Q r \\ &\leq \frac{4}{T_0+1} \sup_{\|u\|_1 \leq 1} (u^\intercal W^\intercal Q r) \\ &= \frac{4}{T_0+1} \|W^\intercal Q r\|_\infty. \end{aligned}$$

Moreover, we have $\widetilde{\Delta}^\intercal J\widetilde{\Delta} \geq \lambda_{\min}(J)\|\widetilde{\Delta}\|_2^2$, and

$$
\begin{aligned}
&\widetilde{\Delta}^\intercal\Big(J - \frac{1}{T_0+1}W^\intercal Q W\Big)\widetilde{\Delta} \\
&= \widetilde{\Delta}^\intercal\bigg\{\frac{1}{T_0+1}\sum_{t\neq s}\Gamma Z_t P_{st} Z_s^\intercal \Gamma^\intercal - \frac{1}{T_0+1}\sum_{t\neq s}(\Gamma Z_t + U_t)P_{st}(\Gamma Z_s + U_s)^\intercal\bigg\}\widetilde{\Delta} \\
&= \widetilde{\Delta}^\intercal\bigg\{-\frac{1}{T_0+1}\sum_{t\neq s}U_t P_{st} Z_s^\intercal \Gamma^\intercal - \frac{1}{T_0+1}\sum_{t\neq s}\Gamma Z_t P_{st} U_s^\intercal - \frac{1}{T_0+1}\sum_{t\neq s}U_t P_{st} U_s\bigg\}\widetilde{\Delta}.
\end{aligned}
$$

This completes the proof. □

Similar to Lemma 1, Lemma 2 provides a deterministic deviation bound for $\tilde{\alpha}_{\mathcal{D}}$. The lemma implies the consistency of $\tilde{\alpha}_{\mathcal{D}}$ whenever the relevant random quantities converge to 0, without requiring covariance-stationarity of the data. Moreover, in contrast to the naive constrained GMM, the ridge-regularized JIVE can effectively handle the case where $d, |\mathcal{D}| \geq n$. To see this, we first remark that $0 \preceq P \preceq I_{T_0+1}$ and $Q \preceq I_{T_0+1}$ since we have

$$
I_{T_0+1} - Q = I_{T_0+1} - P + \mathrm{diag}(P) \succeq 0.
$$

Now, to show that the infinity norm of the vector $(T_0+1)^{-1}\sum_{t\neq s} W_{\mathcal{D}t}P_{st}r_t$ converges to 0, we first remark that the i-th component of the vector is

$$
\frac{1}{T_0+1}\sum_{t\neq s} W_{it}P_{st}r_s = \frac{1}{T_0+1}\sum_{t\neq s}\Gamma_i^\intercal Z_t P_{st} r_s + \frac{1}{T_0+1}\sum_{t\neq s} U_{it}P_{st}r_s, \tag{S.21}
$$

where Γ_i is the i-th row of Γ and U_{it} is the i-th component of U_t. The first term in the right-hand side of (S.21) can be written as $(T_0+1)^{-1}\sum_{t=1}^{T_0+1} A_t r_t$, where $A_t = \sum_{s\neq t}\Gamma_i^\intercal Z_s P_{ts}$.

Conditioning on Z, A_t is a scalar and we have

$$\begin{aligned}
\frac{1}{T_0+1}\sum_{t=1}^{T_0+1} A_t^2 &= \frac{1}{T_0+1}\sum_{t=1}^{T_0+1}\sum_{s,l\neq t}\Gamma_i^{\intercal} Z_s P_{ts}\Gamma_i^{\intercal} Z_l P_{tl} \\
&= \frac{1}{T_0+1}\sum_{t=1}^{T_0+1}\sum_{s,l\neq t}\Gamma_i^{\intercal} Z_s P_{st} P_{tl} Z_l^{\intercal}\Gamma_i \\
&= \frac{1}{T_0+1}\sum_{t=1}^{T_0+1}\sum_{s,l}\Gamma_i^{\intercal} Z_s Q_{st} Q_{tl} Z_l^{\intercal}\Gamma_i \\
&= \frac{1}{T_0+1}\sum_{s,l}\Gamma_i^{\intercal} Z_s (Q^2)_{sl} Z_l^{\intercal}\Gamma_i \\
&\leq \|\Gamma_i\|_2^2 \left\|\frac{1}{T_0+1} Z^T Q^2 Z\right\|_{\text{op}} \leq \|\Gamma_i\|_2^2 \left\|\frac{1}{T_0+1} Z^T Z\right\|_{\text{op}},
\end{aligned}$$

where the second step follows from the definition of Q, and the last step uses the fact that $Q \preceq I$. Provided that $\|\Gamma\|_{\text{op}} = O(1)$, $d/n = O(1)$, each row of Z is sub-Gaussian, and $\lambda_{\max}(\Sigma_{ZZ}) = O(1)$, where $\Sigma_{ZZ} = E(\hat{\Sigma}_{ZZ})$, the last term is bounded with high probability. Therefore, when r_t is sub-Gaussian, it follows that

$$\max_{i\in\mathcal{D}}\left|\frac{1}{T_0+1}\sum_{t\neq s}\Gamma_i^{\intercal} Z_t P_{st} r_s\right| = O_{\text{P}}\left(\sqrt{\frac{\log(|\mathcal{D}|)}{T_0}}\right).$$

For the second term in the right-hand side of (S.21), denote $U_i = (U_{i1}, \ldots, U_{i(T_0+1)})^{\intercal}$. Then, it follows that

$$\frac{1}{T_0+1}\sum_{t\neq s} U_{it} P_{st} r_s = \frac{1}{T_0+1} U_i^{\intercal} Q r = \frac{1}{4(T_0+1)}\{(U_i+r)^{\intercal} Q (U_i+r) - (U_i-r)^{\intercal} Q (U_i - r)\}.$$

Therefore, provided that U_{it} and r_t are sub-Gaussian conditional on Z, applying Hanson-

Wright inequality (see, e.g., Theorem 6.2.1 in Vershynin (2018)), we have

$$\max_{i\in\mathcal{D}} \left|\frac{1}{T_0+1}\sum_{t\neq s} U_{it}P_{st}r_s\right| = O_{\mathrm{P}}\left(\sqrt{\frac{\log(|\mathcal{D}|)}{T_0}}\right).$$

Combining this with the previous bound establishes that

$$\left\|\frac{1}{T_0+1}\sum_{t\neq s} W_{\mathcal{D}t}P_{st}r_s\right\|_\infty = o_{\mathrm{P}}(1),$$

provided that $d/T_0 = O(1)$ and $\log(|\mathcal{D}|) = o(T_0)$ with some regularity conditions. This condition allows both d and $|\mathcal{D}|$ to exceed T_0. Analogous bounds for the remaining terms in Lemma 2 can be established by similar arguments, establishing that $\tilde{\alpha}_{\mathcal{D}}$ is consistent. Then, as in Corollary 3, suppose that $\|(T_0+1)^{-1}\sum_t W_{\mathcal{D}t}W_{\mathcal{D}t}^\intercal\|_{\mathrm{op}}$ is bounded, which holds with high probability when $|\mathcal{D}|/T_0 = O(1)$ under mild regularity conditions on $W_{\mathcal{D}t}$. In this case, $\tilde{\alpha}_{\mathcal{D}}$ satisfies Assumption 3 of Chernozhukov et al. (2021), and the permutation test based on the constrained ridge-regularized JIVE estimator is asymptotically valid as $T_0 \to \infty$.

In sum, by employing the constrained ridge-regularized JIVE estimator, we can accommodate setups where $|\mathcal{D}|, d \leq CT_0$ for some constant $C > 0$, thereby allowing both $|\mathcal{D}|$ and d to be large. Notably, our framework does not require covariance-stationarity, and the argument extends to settings where the data are β-mixing. Since $|\mathcal{D}|$ and T_0 are often of comparable order in SC applications, this allowance is particularly advantageous in practice.

H Proof of Theorems 3 and 4

Proof of Theorem 3: Write f_{λ_t} as the density function for λ_t. We show that given the confounding bridge function, the mean potential outcome is $E[Y_t(0)] = E[h(W_{\mathcal{D}t})]$ $(t \geq 1)$. This is because, for any $t \geq 1$,

$$\begin{aligned} E[Y_t(0) - h(W_{\mathcal{D}t})] &\overset{A.1,A.2'}{=} E[Y_t(0) - h(W_{\mathcal{D}t}(0))] \\ &= E\{E[Y_t(0) - h(W_{\mathcal{D}t}(0)) \mid \lambda_t]\} \\ &\overset{A.3''}{=} 0. \end{aligned} \tag{S.22}$$

It is important to note that to identify the average treatment effect on the treated, one only needs to identify $h(W_{\mathcal{D}t})$, because $E[Y_t(1)] \overset{A.1}{=} E[Y_t]$ in the post-treatment period. In fact, given $h(W_{\mathcal{D}t})$, the average treatment effect on the treated unit in the post-treatment period is identified by

$$E[Y_t(1) - Y_t(0)] = E[Y_t - h(W_{\mathcal{D}t})]. \tag{S.23}$$

Proof of Theorem 4: We show that any h that satisfies Assumption 3" also satisfies $E(Y_t \mid Z_t) = E[h(W_{\mathcal{D}t}) \mid Z_t]$ for all $t \leq T_0$ as follows. For any $t \leq T_0$, we have

$$
\begin{aligned}
E[Y_t \mid Z_t] =& E\{E(Y_t \mid \lambda_t, Z_t) \mid Z_t\} \\
\overset{A.4}{=}& E\{E(Y_t \mid \lambda_t) \mid Z_t\} \\
\overset{A.1}{=}& E\{E(Y_t(0) \mid \lambda_t) \mid Z_t\} \\
\overset{A.3''}{=}& E[E\{h(W_{\mathcal{D}t}(0)) \mid \lambda_t\} \mid Z_t] \\
\overset{A.1}{=}& E[E\{h(W_{\mathcal{D}t}) \mid \lambda_t\} \mid Z_t] \\
\overset{A.4}{=}& E[E\{h(W_{\mathcal{D}t}) \mid \lambda_t, Z_t\} \mid Z_t] \\
=& E[h(W_{\mathcal{D}t}) \mid Z_t]
\end{aligned}
$$

Therefore

$$
E[Y_t - h(W_{\mathcal{D}t}) \mid Z_t] = 0, \quad \forall t \le T_0. \tag{S.24}
$$

I Additional discussion on the completeness condition and existence of multiple confounding bridge functions

In Theorem 4, we showed that every function h satisfying Equation (13) also satisfies Equation (14). We shall prove that the reverse is true given the following additional assumptions:

Assumption I.1 (Latent ignorability)**.** *The joint conditional distribution of* $(Y_t(0), W_{\mathcal{D}t}(0)) \mid \lambda_t$ *is identical for every* $t \geq 1$.

Intuitively, Assumption I.1 requires that λ_t accounts for all common causes of the treatment status at time t and $\{Y_t(0), W_{\mathcal{D}t}\}$ so that there is no remaining confounding.

Assumption I.2 (Completeness)**.**

(a) For any square integrable function q*, if* $E[q(\lambda_t) \mid Z_t] = 0$ *almost surely, then* $q(\lambda_t) = 0$ *almost surely for any* $t \leq T_0$.

(b) For any square integrable function q*, if* $E[q(W_{\mathcal{D}t}) \mid Z_t] = 0$ *almost surely, then* $q(W_{\mathcal{D}t}) = 0$ *almost surely for any* $t \leq T_0$.

Assumption I.2(a) formalizes the requirement that Z_t should be λ_t-relevant, in the sense that any infinitesimal variation in λ_t is captured by variation in Z_t such that no information has been lost through projection of λ_t on Z_t in the pre-treatment period, and the same goes for Assumption I.2(b). As noted in Section F of the Supplementary Materials, Assumption I.2(a) in addition to mild regularity conditions constitutes sufficient conditions for the existence of confounding bridge functions. Completeness is a fundamental statistical concept in minimum variance unbiased estimation and hypothesis testing (Lehmann & Scheffé 2012, Basu 2011)

and has recently been used to establish identification condition in a variety of problems such as nonparametric instrumental variable methods (Newey & Powell 2003, Ai & Chen 2003, Chernozhukov & Hansen 2005, Hall & Horowitz 2005, Darolles et al. 2011, D'Haultfoeuille 2011, Chen et al. 2014), measurement error (Hu & Schennach 2008, Carroll et al. 2010, An & Hu 2012), and missing data (Miao & Tchetgen Tchetgen 2016). It holds in a large class of commonly-used models such as exponential families (Newey & Powell 2003) and location-scale families (Mattner 1992, Hu & Shiu 2018), and nonparametric additive models (Darolles et al. 2011, D'Haultfoeuille 2011). Andrews (2017) introduced a broad nonparametric class of bivariate distributions that satisfy completeness. In practice, we recommend to measure a rich set of proxies to make the completeness assumption plausible. We refer the readers to Miao et al. (2020) for a thorough review and discussion on the completeness condition. Finally, Ghassami et al. (2023) proposed probability bounds of the causal effect estimates using proxies when completeness condition does not hold.

We have the following results, first for identification of the set of confounding bridge functions and second for unique identification of a confounding bridge function:

Corollary 4 (Identification of confounding bridge functions)**.**

(a) Under Assumptions 1, 4, I.1 and I.2(a), any function satisfying Equation (14) is a confounding bridge function that satisfies Equation (13);

(b) Under Assumptions 1, 4, and Assumption I.2(b), there is a unique confounding bridge function that is identified by solving Equation (14).

Proof. (a) Suppose a function h satisfies Equation (14), then for any $t \leq T_0$,

$$
\begin{aligned}
0 &= E[Y_t - h(W_{\mathcal{D}t}) \mid Z_t] \\
&= E[E\{Y_t - h(W_{\mathcal{D}t}) \mid Z_t, \lambda_t\} \mid Z_t] \\
&\overset{A.4}{=} E[E\{Y_t - h(W_{\mathcal{D}t}) \mid \lambda_t\} \mid Z_t]
\end{aligned}
$$

By Assumption I.2(a), we have $E[Y_t - h(W_{\mathcal{D}t}) \mid \lambda_t] = 0$ almost surely for any $t \leq T_0$. By Assumption 1, this is equivalent to $E[Y_t(0) - h(W_{\mathcal{D}t}(0)) \mid \lambda_t] = 0$ almost surely for any $t \leq T_0$.

By Assumption I.1, we have that $E[h(W_{\mathcal{D}t}(0)) \mid \lambda_t] = E[Y_t(0) \mid \lambda_t]$ almost surely for any $t \geq 1$, i.e., the function h satisfies Equation (13).

(b) Let h be a confounding bridge function. We first show the uniqueness of the solution to Equation (14). By Theorem 4, the function satisfies Equation (14). Suppose that both $h(W_{\mathcal{D}t})$ and $h'(W_{\mathcal{D}t})$ satisfy Equation (14) but $h \neq h'$, then we have that $E[h(W_{\mathcal{D}t}) - h'(W_{\mathcal{D}t}) \mid Z_t] = 0$ almost surely. By Assumption I.2(b), it must be that $h' = h$ almost surely, which leads to a contradiction. Next, we show the uniqueness of the confounding bridge function. Suppose there is another confounding bridge function h''. Then by Theorem 4, the function h'' also satisfies Equation (14), which leads to a contradiction. Therefore, the confounding bridge function is unique and is a unique solution to Equation (14).

□

J Additional simulation studies

J.1 Nonstationary latent factor

We generate the data similarly to Section 4, except that the latent factors are $\lambda_t = (\lambda_{t1}, \ldots, \lambda_{tr})^\top$, where λ_{tk}'s are independently sampled from $N(0.5\log(t), 0.5^2)$. That is, the distribution of the latent factors is nonstationary.

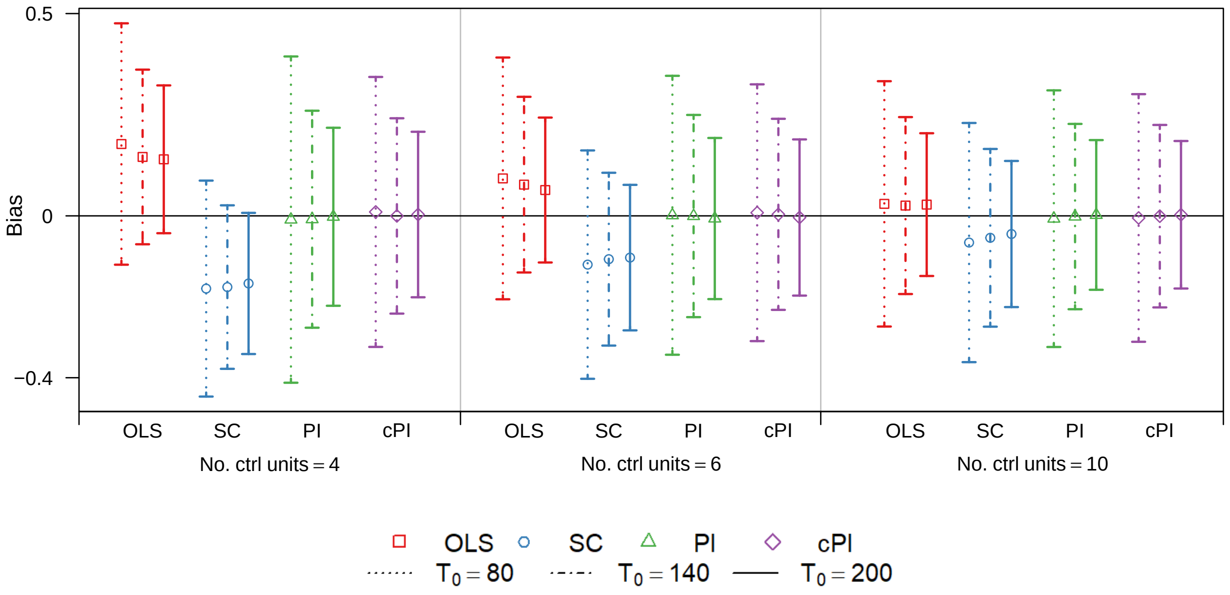

Figure S.1: Bias ± standard deviation of τ estimates based on the unconstrained (OLS) and constrained (SC) regression methods, and our proposed unconstrained (PI) and constrained (cPI) proximal inference methods, with a range of number of control units $N = 4, 6,$ or 10 and pre- and post-treatment time period $T_0 = T_1 = 80, 140,$ or 200.

J.2 Unconstrained SC weights

We generate the data similarly to Section 4, except that for the treated unit, the factor loading is $\mu_0 = (1.5, \ldots, 1.5)^\intercal$. Therefore, the SC weights $\alpha_{\mathcal{D}}$ sum to 1.5 instead of one.

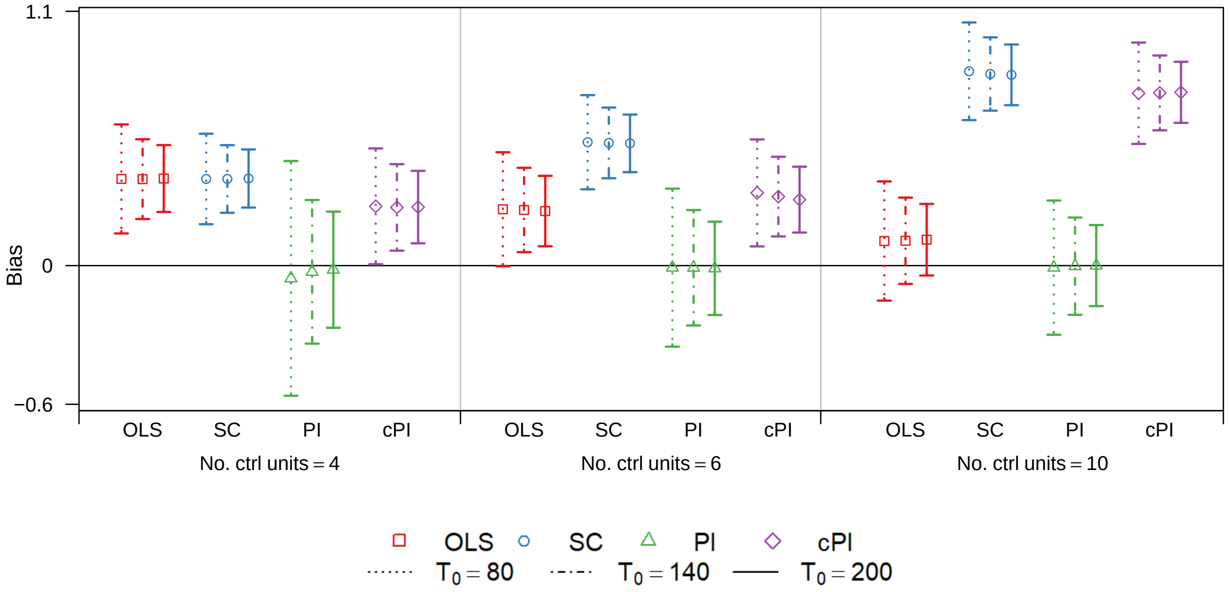

Figure S.2: Bias ± standard deviation of τ estimates based on the unconstrained (OLS) and constrained (SC) regression methods, and our proposed unconstrained (PI) and constrained (cPI) proximal inference methods, with a range of number of control units $N = 4, 6,$ or 10 and pre- and post-treatment time period $T_0 = T_1 = 80, 140,$ or 200.

J.3 Weakly dependent errors

We generate the data similarly to Section 4, except that ε_{it} is an AR(1) process with coefficient 0.1, i.e., $\varepsilon_{it} = 0.1\varepsilon_{i,t-1} + \nu_{it}$ with $\nu_{it} \overset{i.i.d}{\sim} N(0,1)$.

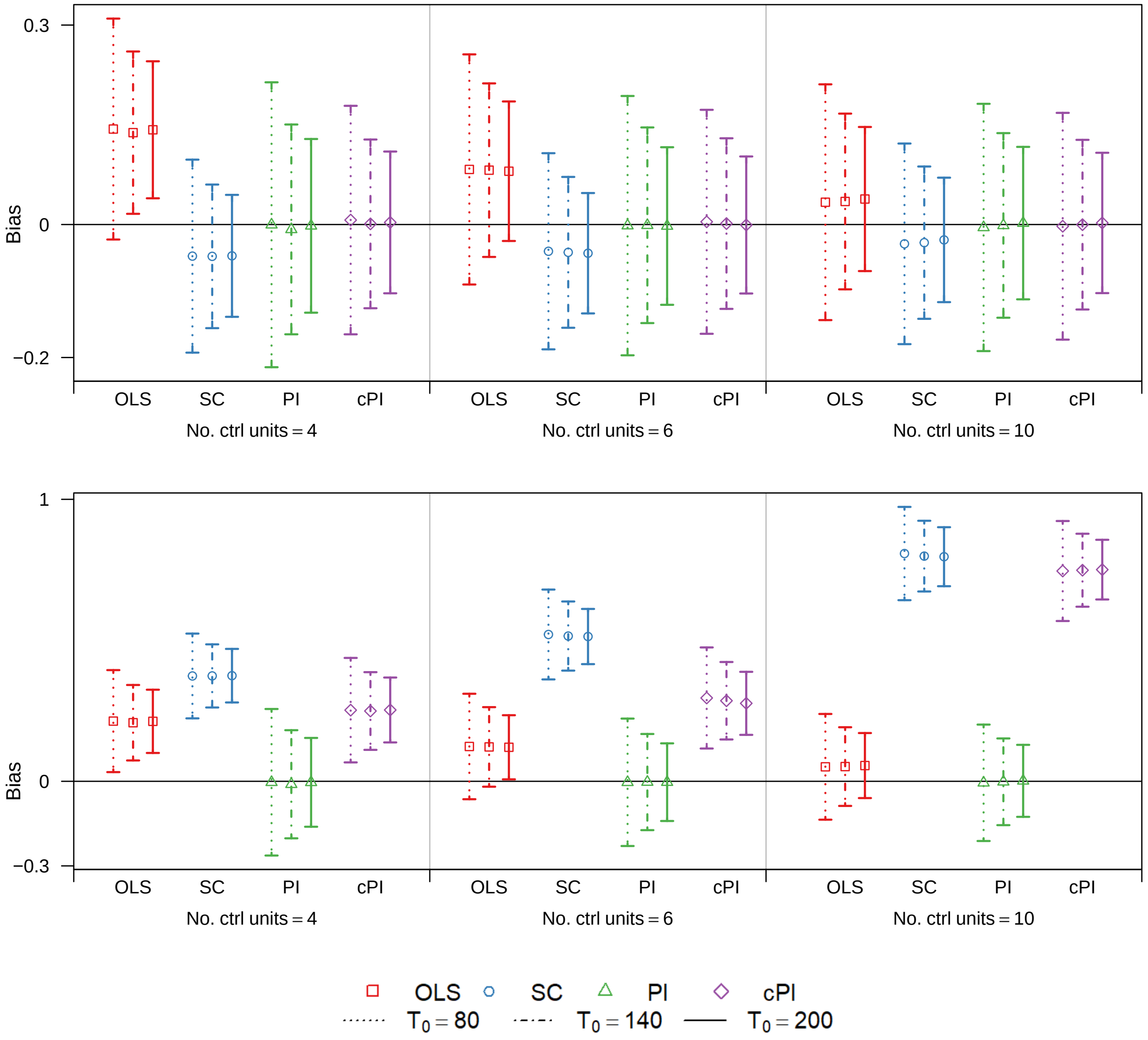

Figure S.3: Bias ± standard deviation of τ estimates based on the unconstrained (OLS) and constrained (SC) regression methods, and our proposed unconstrained (PI) and constrained (cPI) proximal inference methods, with a range of number of control units $N = 4, 6,$ or 10 and pre- and post-treatment time period $T_0 = T_1 = 80, 140,$ or 200.

J.4 Small number of pre-treatment periods

We repeat the simulation studies in Section 4 as well as Sections I.1-I.3 in the Supplementary Material, but set the number of the pre-treatment periods as $T_0 = 30$. We present the bias, median bias ($|\widehat{\tau} - \tau|$), and standard deviation of the estimators over 5,000 replications in Table S.1.

In the settings where T_0 is small, while PI has small median bias and so does cPI in settings where the true SC weights satisfy the simplex constraints, both estimators occasionally exhibit large mean bias and standard deviation. This raises caution that these two estimators may be unstable in the small T_0 setting.

We conclude that, in practice, when the number of pre-treatment periods is small, comparison of the proposed PI and cPI versus the standard SC method is critical for the method with the optimal bias/variance tradeoff. The placebo falsification test as described in Section 5 may be a useful tool assist with the method selection.

Table S.1: Bias, median bias (MB), and standard deviation of τ estimates based on the unconstrained (OLS) and constrained (SC) regression methods, and our proposed unconstrained (PI) and constrained (cPI) proximal inference methods, with a range of number of control units $N = 4, 6,$ or 10 and pre- and post-treatment time period $T_0 = T_1 = 50, 100,$ or 200.

λ	α	#. Ctrls	OLS			SC			PI			cPI		
			Bias	MB	SD	Bias	MB	SD	Bias	MB	SD	Bias	MB	SD
stat.	const.	4	0.19	0.19	0.35	-0.05	-0.04	0.30	0.18	0.02	17.05	0.22	-0.01	16.64
stat.	const.	6	0.12	0.12	0.37	-0.04	-0.04	0.30	1.44	0.00	139.57	0.00	0.00	0.36
stat.	const.	10	0.05	0.04	0.41	-0.04	-0.04	0.31	1.56	-0.01	157.04	0.00	-0.01	0.36
stat.	unconst.	4	0.29	0.29	0.38	0.37	0.38	0.31	0.22	0.03	21.05	0.54	0.27	20.49
stat.	unconst.	6	0.17	0.17	0.41	0.53	0.53	0.32	1.77	0.01	172.53	0.34	0.34	0.63
stat.	unconst.	10	0.08	0.07	0.43	0.85	0.84	0.33	2.20	0.00	221.56	0.76	0.75	0.37
nonstat.	const.	4	0.17	0.17	0.48	-0.10	-0.09	0.39	0.00	0.01	4.81	-0.02	0.00	1.71
nonstat.	const.	6	0.09	0.09	0.50	-0.08	-0.07	0.42	-0.18	0.00	26.90	-0.25	0.00	26.19
nonstat.	const.	10	0.03	0.03	0.52	-0.05	-0.05	0.43	-0.01	-0.01	0.58	0.00	0.00	0.49
nonstat.	unconst.	4	0.26	0.26	0.53	0.86	0.87	0.42	-0.01	0.01	6.43	0.27	0.29	2.18
nonstat.	unconst.	6	0.13	0.13	0.54	1.11	1.09	0.39	-0.21	0.01	28.20	0.68	0.95	27.47
nonstat.	unconst.	10	0.05	0.04	0.55	2.85	2.85	0.33	-0.01	0.00	0.65	2.85	2.85	0.56

J.5 Randomly selected donor and proxy units

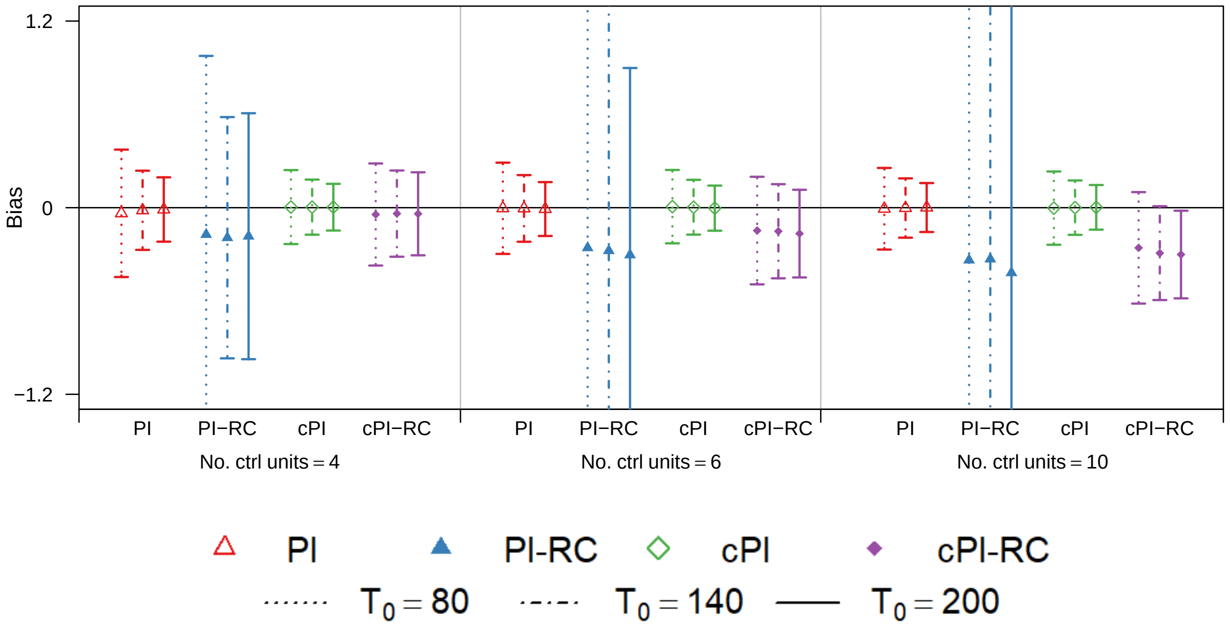

Figure S.4: Bias ± standard deviation of τ estimates based on the unconstrained (OLS) and constrained (SC) regression methods, and our proposed unconstrained (PI) and constrained (cPI) proximal inference methods, with a range of number of control units $N = 4, 6,$ or 10 and pre- and post-treatment time period $T_0 = T_1 = 80, 140,$ or 200.

J.6 Covariate adjustment

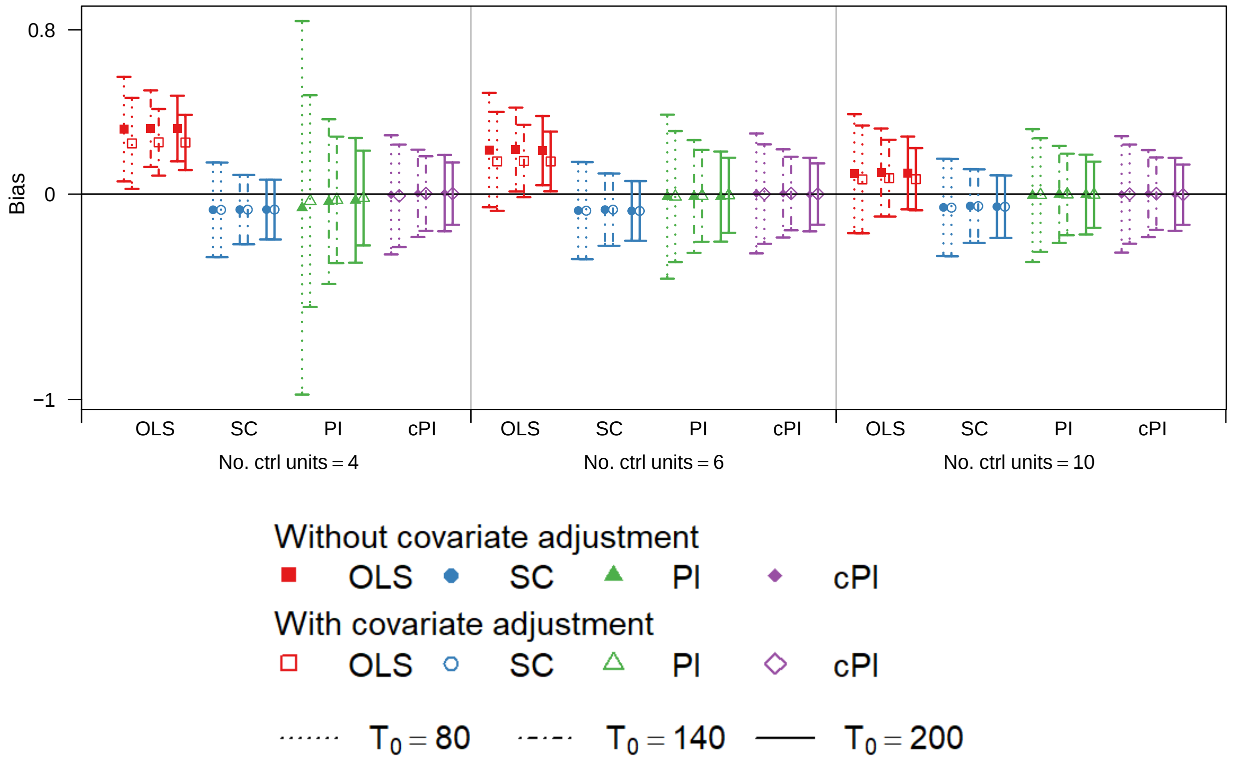

Figure S.5: Bias ± standard deviation of τ estimates based on the unconstrained (OLS) and constrained (SC) regression methods, and our proposed unconstrained (PI) and constrained (cPI) proximal inference methods, with or without covariate adjustment. We considered a range of number of control units $N = 4, 6,$ or 10 and pre- and post-treatment time period $T_0 = T_1 = 80, 140,$ or 200.

J.7 Time-varying treatment effect

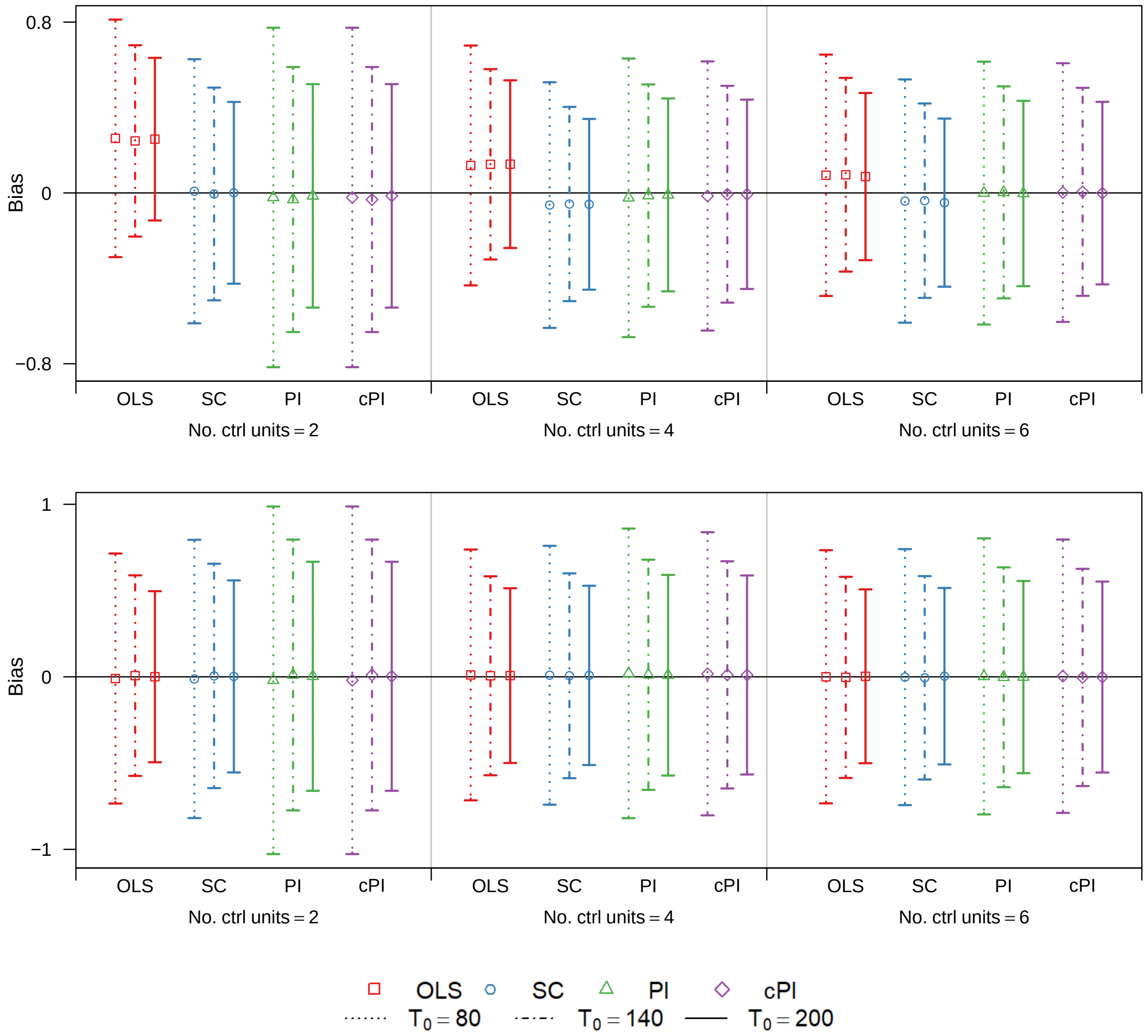

Figure S.6: Bias ± standard deviation of the γ_0 (first row) and γ_1 (second row) estimates based on the unconstrained (OLS) and constrained (SC) regression methods, and our proposed unconstrained (PI) and constrained (cPI) proximal inference methods, with a range of number of control units $N = 2, 4,$ or 6 and pre- and post-treatment time period $T_0 = T - T_0 = 80, 140,$ or 200.

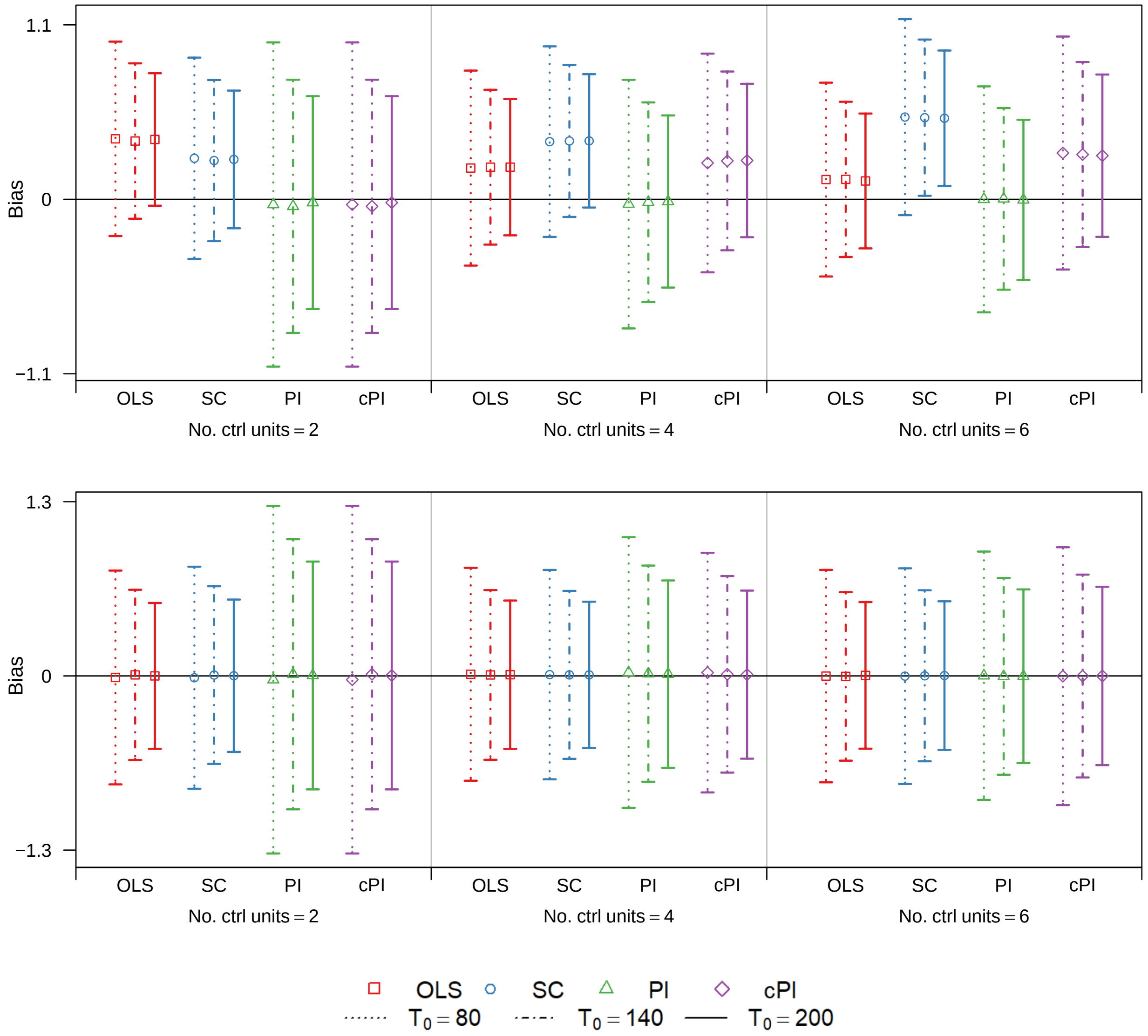

Figure S.7: Bias ± standard deviation of the γ_0 (first row) and γ_1 (second row) estimates based on the unconstrained (OLS) and constrained (SC) regression methods, and our proposed unconstrained (PI) and constrained (cPI) proximal inference methods, with a range of number of control units $N = 2, 4,$ or 6 and pre- and post-treatment time period $T_0 = T - T_0 = 80, 140,$ or 200.

We simulate time series data on N control units and one treated unit over $T_0 = 80, 140,$ or 200 time periods pre-treatment and the same time length post-treatment, i.e., $T = 2T_0$. We gen-

erate samples under the following data generating mechanism

$$Y_t = \begin{cases} \theta(t/T;\gamma) + \mu_0^\intercal \lambda_t + \varepsilon_{0t}, & t > T_0 \\ \mu_0^\intercal \lambda_t + \varepsilon_{0t}, & t \leq T_0 \end{cases}$$

$$W_{it} = \mu_i^\intercal \lambda_t + \varepsilon_{it},$$

where $\varepsilon_{it} \overset{i.i.d}{\sim} N(0,1)$ and $\tau(t/T;\gamma) = \gamma_0 + \gamma_1 t/T$ with $\gamma = (2, 0.4)^\intercal$. The rest of the data generating setting is the same as Section 4. We focus on estimation and inference of γ using the same approach as detailed in Section 4. Below we present the simulation results in Figures S.6 and S.7. For estimation of γ_0, the relative performance comparing the four methods are similar to Section 4; for estimation of γ_1, however, all four methods perform similarly and produce nearly unbiased point estimators, even for SC and cPI methods when the SC weights do not sum to one.

J.8 Nonlinear model

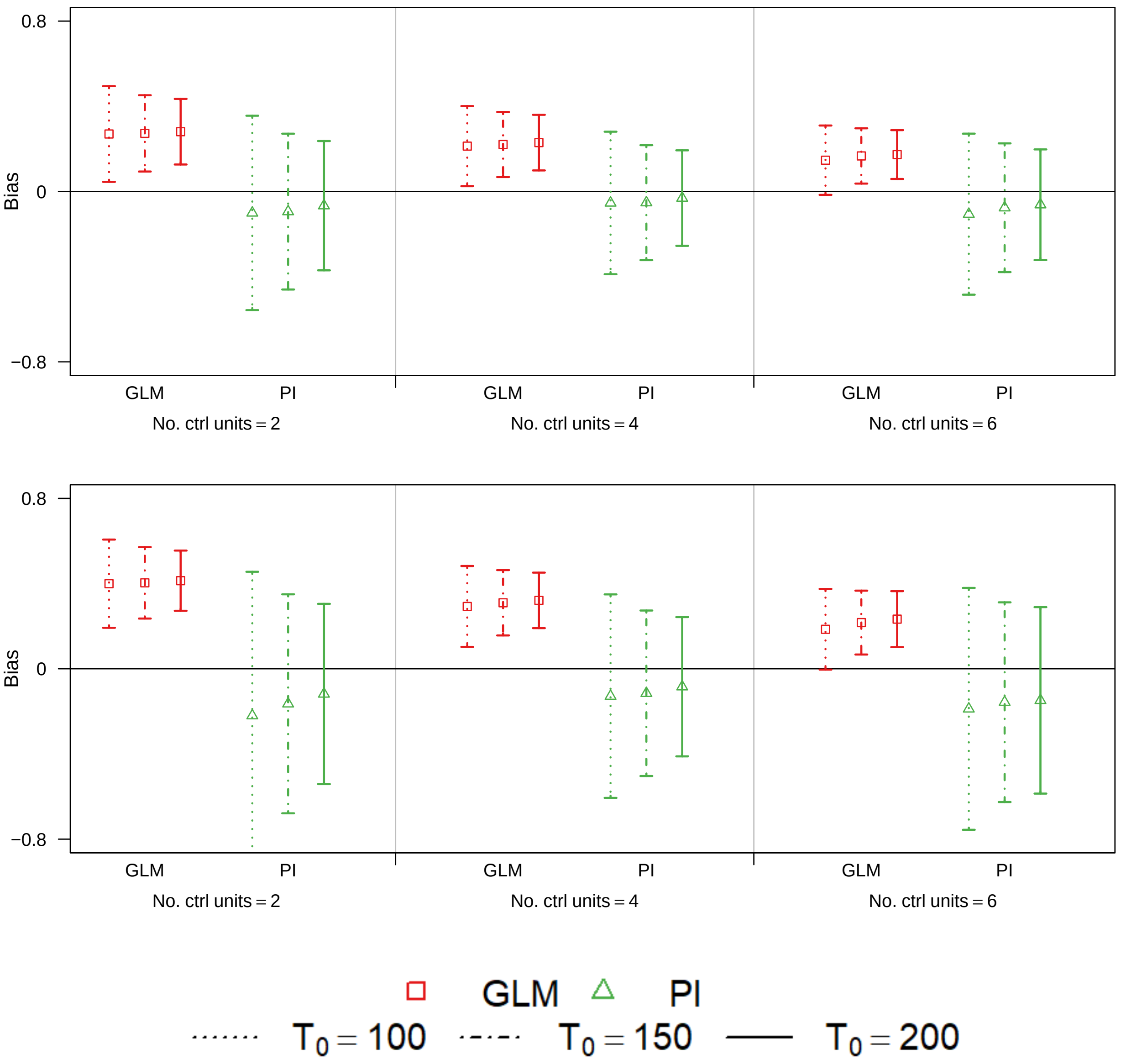

Figure S.8: Bias ± standard deviation of the β estimates based on the unconstrained log-linear regression method (GLM) and our proposed unconstrained proximal inference method (PI), with a range of number of control units $N = 2, 4,$ or 6 and pre- and post-treatment time period $T_0 = T - T_0 = 100, 150,$ or 200.

We simulate time series data on N control units and one treated unit over $T_0 = 100, 150,$ or 200 time period pre-treatment and the same time length post-treatment, i.e., $T = 2T_0$. We gen-

erate Y_t and W_{it} $i = 1, \ldots, N$ from Poisson distribution with

$$E[Y_t \mid X_t, \lambda_t] = \begin{cases} \exp(\beta + \mu_0^\intercal \lambda_t), & t > T_0 \\ \exp(\mu_0^\intercal \lambda_t), & t \leq T_0 \end{cases} \tag{S.25}$$

$$E[W_{it} \mid \lambda_t] = (\mu_i^\intercal \lambda_t)/(e-1),$$

where $X_t = 1$ if $t > T_0$, and $X_t = 0$ otherwise; $\beta = -2$; and $\zeta = 0.1$ or 0 corresponding to scenarios with and without measured covariates. We set β to -2 to avoid extremely large variance of the simulated outcomes due to the mean-variance relationship of Poisson distribution. We simulate a vector of latent factors from independent normal distribution $N(0.5, 0.1^2)$ truncated at $(0, 1)$. We generate three settings with one, two, or three latent factors. We reduce the number of latent factors compared to previous simulation studies to achieve a more stable result for illustration purpose. For each setting, we assume the number of control units is twice the number of latent factors, i.e. $N = 2r.$, and the first half of the control units $(i = 1, \ldots, r)$ constitute the donor pool with $|\mathcal{D}| = r$. We specify factor loadings in the same way in Section 4.

From the above model we know that $\beta = E[Y_t(1)]/E[Y_t(0)] = -2$. We focus on estimation of β in this setting for simplicity. We implement the PI method taking the first half of control units as donors $W_{\mathcal{D}t}$ and second half of control units as supplemental proxies $W_{\overline{\mathcal{D}}t}$. When there exists a measured covariate (i.e., $\xi = 0.1$) which is predictive of the outcome, we implement our method with and without covariate adjustment to investigate whether there is an efficiency gain from adjusting for such a predictor of the outcome. From Eq. (S.25),

we derived that the confounding bridge function satisfying Assumption 3' is given by

$$h(W_{\mathcal{D}t}; \alpha_{\mathcal{D}}, \xi) = \exp[\sum_{i \in \mathcal{D}} \alpha_i W_{it}].$$

We present the simulation results in terms of bias, variance, and coverage probability in Figure S.8.

J.9 Prediction intervals for θ_{T_0+1}: nonstationary latent factors or unconstrained SC weights

Table S.2: Coverage (Average length) of 90% prediction intervals over 5,000 Monte Carlo samples for θ_{T_0+1} using the permutation inference approach or SCPI approach in Sections 3.2.1 and 3.2.2 with stationary latent factors and unconstrained SC weights.

No. ctrl	T_0	Permutation Inference				SCPI			
		OLS	SC	PI	cPI	OLS	SC	PI	cPI
5	80	90.2% (5.7)	88.7% (5.5)	90.1% (7.2)	89.4% (6.2)	97.3% (8.1)	92.9% (6.7)	98.8% (15.9)	91.4% (6.5)
	140	89.8% (5.6)	89.1% (5.6)	90.5% (6.7)	90.4% (6.2)	95.8% (7.2)	92.4% (6.3)	98.3% (13.3)	90.7% (6.1)
	200	90.0% (5.5)	89.0% (5.5)	89.9% (6.5)	89.3% (6.2)	95.3% (6.8)	92.7% (6.2)	98.2% (12.1)	90.4% (5.8)
7	80	90.0% (5.7)	87.3% (5.3)	89.6% (6.5)	88.3% (5.9)	98.3% (9.2)	93.6% (7.0)	99.7% (19.7)	94.0% (7.6)
	140	90.0% (5.5)	88.6% (5.3)	90.5% (6.1)	89.6% (5.8)	97.6% (8.1)	94.2% (6.7)	99.6% (16.3)	94.2% (7.3)
	200	89.4% (5.4)	88.6% (5.3)	90.0% (6.0)	89.4% (5.8)	96.9% (7.5)	94.3% (6.6)	99.4% (14.9)	94.5% (7.2)
11	80	90.5% (5.7)	86.9% (5.0)	90.8% (6.0)	89.0% (5.5)	99.5% (11.3)	94.8% (7.4)	100% (24.9)	97.3% (9.7)
	140	90.0% (5.4)	87.3% (5.1)	90.5% (5.7)	89.0% (5.5)	99.2% (9.5)	95.9% (7.3)	100% (21.1)	98.3% (9.9)
	200	89.7% (5.4)	88.0% (5.1)	89.9% (5.6)	89.1% (5.5)	98.5% (8.7)	96.1% (7.1)	99.9% (19.1)	98.7% (9.9)

Table S.3: Coverage (Average length) of 90% prediction intervals over 5,000 Monte Carlo samples for θ_{T_0+1} using the permutation inference approach or SCPI approach in Sections 3.2.1 and 3.2.2 with stationary latent factors and unconstrained SC weights.

No. ctrl	T_0	Permutation Inference				SCPI			
		OLS	SC	PI	cPI	OLS	SC	PI	cPI
5	80	90.0% (6.0)	88.7% (5.8)	90.1% (8.8)	89.5% (6.7)	96.6% (8.1)	93.2% (6.8)	98.3% (12.5)	91.0% (6.8)
	140	89.4% (5.9)	88.5% (5.8)	90.8% (8.2)	89.3% (6.6)	95.2% (7.3)	92.6% (6.5)	97.8% (10.4)	91.7% (6.6)
	200	90.1% (5.9)	89.6% (5.8)	89.8% (7.9)	89.9% (6.5)	94.6% (6.9)	92.4% (6.4)	96.9% (9.3)	91.4% (6.4)
7	80	89.9% (6.0)	88.2% (5.7)	89.5% (7.6)	89.6% (6.6)	98.0% (9.4)	92.4% (7.0)	98.7% (12.6)	93.8% (7.8)
	140	89.8% (5.8)	89.0% (5.8)	90.3% (7.1)	90.0% (6.6)	97.5% (8.3)	93.1% (6.7)	98.1% (10.3)	93.3% (7.3)
	200	89.4% (5.8)	88.8% (5.8)	90.1% (7.0)	89.1% (6.6)	96.4% (7.7)	93.1% (6.7)	97.5% (9.3)	91.8% (6.9)
11	80	90.4% (5.9)	86.6% (6.1)	90.4% (6.6)	88.6% (7.0)	99.6% (11.5)	91.5% (7.3)	99.5% (13.2)	94.9% (9.0)
	140	90.0% (5.7)	88.3% (6.2)	90.0% (6.3)	88.9% (6.8)	99.1% (9.8)	93.9% (7.3)	99.2% (10.9)	95.1% (8.3)
	200	89.9% (5.6)	89.2% (6.2)	89.6% (6.2)	88.9% (6.8)	98.6% (9.0)	94.7% (7.2)	98.6% (9.8)	95.0% (7.9)

K Additional results for the 1990 German reunification analysis

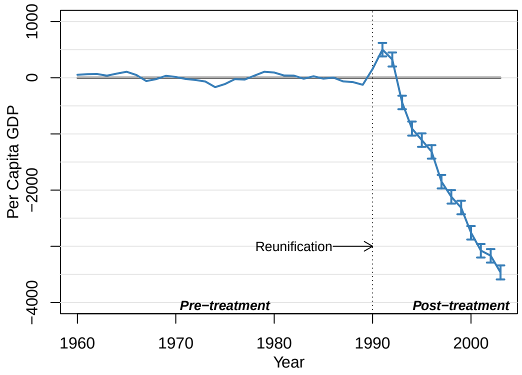

(a) Constrained OLS

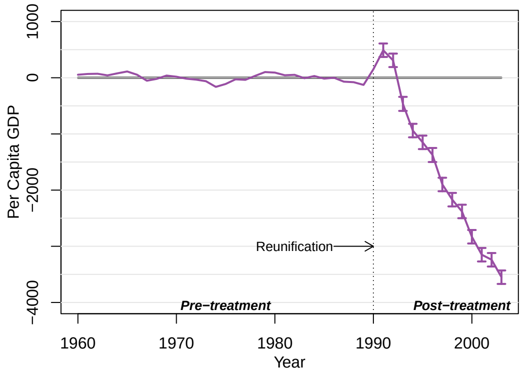

(b) Constrained proximal inference

Figure S.9: Effect trajectory in per-capita GDP in West Germany and 90% pointwise prediction intervals of intervention effects using the conformal permutation approach in Chernozhukov et al. (2021): (a) constrained OLS approach; (b) constrained proximal inference approach.

L Additional discussions

L.1 Comparison between our method and that of Freyberger (2018)

1. In the setting of Freyberger (2018), data are available on many units measured over a few time periods ($N \to \infty$, T fixed), while we consider the setting of a few units measured over a prolonged time (N fixed, $T \to \infty$). For example, Freyberger (2018) considered an application with $T = 6$ and $N = 1,739$.

2. Freyberger (2018) assumes the residual errors and latent factors are jointly independent and that residual errors given latent factors are independent across both time and units, in which case lagged time outcomes may serve as a valid instrumental variable. We allow for serially correlated residual errors and latent factors, and therefore lagged outcomes may not be valid instruments.

3. Because of their additional assumptions, Freyberger (2018) identifies both the distribution of latent factors and random error. We do not aim to estimate these nuisance parameters.

4. In their nonlinear setting, Freyberger (2018) still require that the latent factors and factor loading enter the model in a linear structure on a scale given by an unrestricted transformation. We allow the latent factor to enter the model through an arbitrary (nonlinear) confounding bridge function in Section 3.3.

L.2 Comparison between our method and the interactive fixed effects model based approach

Gobillon & Magnac (2016) develops estimators for treatment effects using panel data un-

der an interactive fixed effects model (Bai 2009). Their framework accommodates multiple treated and untreated units, in contrast to the traditional SC setting, the main focus of our paper, where only a single unit is treated. Formal inference methods under the factor model setting are proposed elsewhere (Li 2018); however, both the estimation and inference rely heavily on linear factor structure assumptions and, in particular, on correctly identifying or estimating the number of latent factors. Moreover, their frameworks cannot easily be extended to nonlinear settings. In contrast, our method does not require estimating the number of unknown factors and can be generalized to nonparametric identification, which represents one of the main contributions of our work, as discussed in Section 3.3. Finally, the asymptotic theory of the interactive fixed effects model based approach require both the number of units and time-periods to be large, suggesting that this approach is better suited for a different setting from our approach or the IV method.

References

Ai, C. & Chen, X. (2003), 'Efficient Estimation of Models with Conditional Moment Restrictions Containing Unknown Functions', *Econometrica* **71**(6), 1795–1843.

An, Y. & Hu, Y. (2012), 'Well-posedness of measurement error models for self-reported data', *Journal of Econometrics* **168**(2), 259–269.

Andrews, D. W. (1988), 'Laws of large numbers for dependent non-identically distributed random variables', *Econometric theory* **4**(3), 458–467.

Andrews, D. W. (1999), 'Estimation when a parameter is on a boundary', *Econometrica* **67**(6), 1341–1383.

Andrews, D. W. (2017), 'Examples of l2-complete and boundedly-complete distributions', *Journal of econometrics* **199**(2), 213–220.

Bai, J. (2009), 'Panel Data Models With Interactive Fixed Effects', *Econometrica* **77**(4), 1229–1279.

Basu, D. (2011), On Statistics Independent of a Complete Sufficient Statistic, *in* 'Selected Works of Debabrata Basu', Springer, pp. 61–64.

Bierens, H. J. (1981), *Robust Methods and Asymptotic Theory in Nonlinear Econometrics*, Springer-Verlag, New York.

Carroll, R. J., Chen, X. & Hu, Y. (2010), 'Identification and estimation of nonlinear models using two samples with nonclassical measurement errors', *Journal of Nonparametric Statistics* **22**(4), 379–399.

Cattaneo, M. D., Feng, Y. & Titiunik, R. (2021), 'Prediction intervals for synthetic control methods', *Journal of the American Statistical Association* **116**(536), 1865–1880.

Chen, X., Chernozhukov, V., Lee, S. & Newey, W. K. (2014), 'Local Identification of Nonparametric and Semiparametric Models', *Econometrica* **82**(2), 785–809.

Chernozhukov, V. & Hansen, C. (2005), 'An IV Model of Quantile Treatment Effects', *Econometrica* **73**(1), 245–261.

Chernozhukov, V., Wuthrich, K. & Zhu, Y. (2018), 'A t-test for synthetic controls', *arXiv preprint arXiv:1812.10820* .

Chernozhukov, V., Wüthrich, K. & Zhu, Y. (2021), 'An exact and robust conformal inference method for counterfactual and synthetic controls', *Journal of the American Statistical Association* **116**(536), 1849–1864.

Cui, Y., Pu, H., Shi, X., Miao, W. & Tchetgen Tchetgen, E. (2024), 'Semiparametric proximal causal inference', *Journal of the American Statistical Association* **119**(546), 1348–1359.

Darolles, S., Fan, Y., Florens, J. P. & Renault, E. (2011), 'Nonparametric Instrumental Regression', *Econometrica* **79**(5), 1541–1565.

Davidson, J. (1994), *Stochastic limit theory: An introduction for econometricians*, OUP Oxford.

Doudchenko, N. & Imbens, G. W. (2016), Balancing, regression, difference-in-differences and synthetic control methods: A synthesis, Technical report, National Bureau of Economic Research.

Doukhan, P. (1994), *Mixing: Properties and Examples*, Springer.

D'Haultfoeuille, X. (2011), 'On the Completeness Condition in Nonparametric Instrumental Problems', *Econometric Theory* **27**(3), 460–471.

Freyberger, J. (2018), 'Non-parametric panel data models with interactive fixed effects', *The Review of Economic Studies* **85**(3), 1824–1851.

Ghassami, A., Shpitser, I. & Tchetgen Tchetgen, E. (2023), 'Partial identification of causal effects using proxy variables', *arXiv preprint arXiv:2304.04374* .

Gobillon, L. & Magnac, T. (2016), 'Regional policy evaluation: Interactive fixed effects and synthetic controls', *The Review of Economics and Statistics* **98**(3), 535–551.

Hall, A. R. (2005), *Generalized Method of Moments*, Oxford University Press.

Hall, P. & Horowitz, J. L. (2005), ‘Nonparametric Methods for Inference in the Presence of Instrumental Variables’, *The Annals of Statistics* **33**(6), 2904–2929.

Hansen, C. & Kozbur, D. (2014), ‘Instrumental variables estimation with many weak instruments using regularized jive’, *Journal of Econometrics* **182**(2), 290–308.

Hsiao, C., Ching, S. H. & Wan, S. K. (2012), ‘A panel data approach for program evaluation: Measuring the benefits of political and economic integration of Hong Kong with mainland China’, *Journal of Applied Econometrics* **27**(5), 705–740.

Hu, Y. & Schennach, S. M. (2008), ‘Instrumental Variable Treatment of Nonclassical Measurement Error Models’, *Econometrica* **76**(1), 195–216.

Hu, Y. & Shiu, J.-L. (2018), ‘Nonparametric Identification Using Instrumental Variables: Sufficient Conditions for Completeness’, *Econometric Theory* **34**(3), 659–693.

Ketz, P. (2018), ‘Subvector inference when the true parameter vector may be near or at the boundary’, *Journal of Econometrics* **207**(2), 285–306.

Kress, R. (1989), *Linear Integral Equations*, Vol. 82, Springer.

Lehmann, E. L. & Scheffé, H. (2012), Completeness, Similar Regions, and Unbiased Estimation-Part I and Part II, *in* ‘Selected Works of E. L. Lehmann’, Springer, pp. 233–286.

Li, J. (2024), ‘Inference for constrained extremum estimators’, *Working Paper* .

Li, K. (2018), ‘Inference for factor model based average treatment effects’, *Available at SSRN 3112775* .

Masini, R. & Medeiros, M. C. (2021), ‘Counterfactual analysis with artificial controls: Inference, high dimensions, and nonstationarity’, *Journal of the American Statistical Association* **116**(536), 1773–1788.

Mattner, L. (1992), ‘Completeness of location families, translated moments, and uniqueness of charges’, *Probability Theory and Related Fields* **92**(2), 137–149.

Miao, W., Geng, Z. & Tchetgen Tchetgen, E. J. (2018), 'Identifying causal effects with proxy variables of an unmeasured confounder', *Biometrika* **105**(4), 987–993.

Miao, W., Hu, W., Ogburn, E. L. & Zhou, X. (2020), 'Identifying effects of multiple treatments in the presence of unmeasured confounding', *arXiv preprint arXiv:2011.04504* .

Miao, W. & Tchetgen Tchetgen, E. J. (2016), 'On varieties of doubly robust estimators under missingness not at random with a shadow variable', *Biometrika* **103**(2), 475–482.

Newey, W. K. & McFadden, D. (1994), 'Large sample estimation and hypothesis testing', *Handbook of Econometrics* **4**, 2111–2245.

Newey, W. K. & Powell, J. L. (2003), 'Instrumental Variable Estimation of Nonparametric Models', *Econometrica* **71**(5), 1565–1578.

Newey, W. K. & West, K. D. (1986), A Simple, Positive Semi-definite, Heteroskedasticity and Autocorrelation Consistent Covariance Matrix, Technical report, National Bureau of Economic Research.

Pötscher, B. M. & Prucha, I. (1997), *Dynamic Nonlinear Econometric Models: Asymptotic Theory*, Springer Science & Business Media.

Vershynin, R. (2018), *High-Dimensional Probability: An Introduction with Applications in Data Science*, Vol. 47 of *Cambridge Series in Statistical and Probabilistic Mathematics*, Cambridge University Press, Cambridge.

White, H. (1980), 'A Heteroskedasticity-consistent Covariance Matrix Estimator and a Direct Test for Heteroskedasticity', *Econometrica* pp. 817–838.

Xu, Y. (2017), 'Generalized Synthetic Control Method: Causal Inference With Interactive Fixed Effects Models', *Political Analysis* **25**(1), 57–76.

Zhang, J., Li, W., Miao, W. & Tchetgen, E. T. (2023), 'Proximal causal inference without uniqueness assumptions', *Statistics & Probability Letters* **198**, 109836.
