# Supplementary material for: Theory for Identification and Inference with Synthetic Controls: A Proximal Causal Inference Framework
Source: J Am Stat Assoc. Author manuscript; Available in PMC 2026 Jul 21. (PMC13384440; doi:10.1080/01621459.2026.2639734)
Supplement: Supp 1 [file NIHMS2185023-supplement-Supp_1.zip › uasa_a_2639734_sm4115.docx]

Author Contributions Checklist Form

This form documents the artifacts associated with the article (i.e., the data and code supporting the computational findings) and describes how to reproduce the findings.

# Part 1: Data

This paper **does not** involve analysis of external data (i.e., no data are used or the only data are generated by the authors via simulation in their code).

I certify that the author(s) of the manuscript have legitimate access to and permission to use the data used in this manuscript.

## Abstract

The German reunification dataset came from a comparative case study of the 1990 German reunification. Abadie et al. (2015) studied the effect of the German reunification on per-capita GDP in West Germany using the synthetic control approach. The dataset contains annual country-level panel data in 1960-2003 for both West Germany, the treated unit, and 16 untreated Organisation for Economic Co-operation and Development (OECD) countries.

## Availability

Data **are** publicly available

Data **cannot be made** publicly available

If the data are publicly available, see the *Publicly available data* section. Otherwise, see the *Non-publicly available dat*a section, below.

### Publicly available data

Data are available online at: The R package scpi or https://dataverse.harvard.edu/dataset.xhtml?persistentId=doi:10.7910/DVN/24714

Data are available as part of the paper’s supplementary material.

Data are publicly available by request, following the process described here:

Data are or will be made available through some other mechanism, described here:

### Non-publicly available data

Discussion of lack of publicly available data:

## Description

### File format(s)

CSV or other plain text: tab data from https://dataverse.harvard.edu/file.xhtml?persistentId=doi:10.7910/DVN/24714/4UPGON&version=2.1

Software-specific binary format (.Rda, Python pickle, etc.): the object scpi_germany from the R package scpi

Standardized binary format (e.g., netCDF, HDF5, etc.):

Other (described here):

### Data dictionary

Provided by the authors in the following file(s):

Data file(s) is (are) self-describiing (e.g., netCDF files)

Available at the following URL:

https://rdrr.io/cran/scpi/man/scpi_germany.html

### Additional information (optional)

# Part 2: Code

## Abstract

R code for performing all simulation studies and data analysis in the manuscript “Theory for Identification and Inference with Synthetic Controls: A Proximal Causal Inference Framework” is available on GitHub (https://github.com/KenLi93/proximal_sc_manuscript/). The simulation was performed on a high-performance computing system (HPC) using multi-core parallelization.

## Description

### Code format(s)

Script files

R  Python  Matlab

Other:

Package

R  Python  MATLAB toolbox

Other:

Reproducible report

R Markdown  Jupyter notebook

Other:

Shell script

Other (described here):

### **Supporting software requirements**

Version of primary software used

R 4.3.2

Libraries and dependencies used by the code

dplyr 1.1.4, foreign 0.8-86, tidyr 1.3.1 scpi 3.0.1, Rsolnp 2.0.1, RColorBrewer 1.1-3, sandwich 3.1-1

### Supporting system/hardware requirements (optional)

### Parallelization used

No parallel code used

Multi-core parallelization on a single machine/node

Number of cores used:

Multi-machine/multi-node parallelization

Number of nodes and cores used:

### License

MIT License (default)

BSD

GPL v3.0

Creative Commons

Other (described here):

### Additional information (optional)

# Part 3: Reproducibility workflow

## Scope

The provided workflow reproduces:

Any numbers provided in text in the paper

The computational method(s) presented in the paper (i.e., code is provided that implements the method(s))

All tables and figures in the paper

Selected tables and figures in the paper, as explained and justified here:

## Workflow details

### Format(s)

Single master code file

Wrapper (shell) script(s)

Self-contained R Markdown file, Jupyter notebook, or other literate programming approach

Text file (e.g., a readme-style file) that documents workflow

Makefile

Other (more detail in 'Instructions' below)

### Instructions

Expected run-time

Approximate time needed to reproduce the analyses on a standard desktop machine:

<1 minute

1-10 minutes

10-60 minutes

1-8 hours

>8 hours

Not feasible to run on a desktop machine, as described here:

### Additional documentation (optional)

# Notes (optional)
